# Data from a pre-publication independent replication initiative examining ten moral judgement effects

**DOI:** 10.1038/sdata.2016.82

**Published:** 2016-10-11

**Authors:** Warren Tierney, Martin Schweinsberg, Jennifer Jordan, Deanna M. Kennedy, Israr Qureshi, S. Amy Sommer, Nico Thornley, Nikhil Madan, Michelangelo Vianello, Eli Awtrey, Luke Lei Zhu, Daniel Diermeier, Justin E. Heinze, Malavika Srinivasan, David Tannenbaum, Eliza Bivolaru, Jason Dana, Clintin P. Davis-Stober, Christilene du Plessis, Quentin F. Gronau, Andrew C. Hafenbrack, Eko Yi Liao, Alexander Ly, Maarten Marsman, Toshio Murase, Michael Schaerer, Christina M. Tworek, Eric-Jan Wagenmakers, Lynn Wong, Tabitha Anderson, Christopher W. Bauman, Wendy L. Bedwell, Victoria Brescoll, Andrew Canavan, Jesse J. Chandler, Erik Cheries, Sapna Cheryan, Felix Cheung, Andrei Cimpian, Mark A. Clark, Diana Cordon, Fiery Cushman, Peter H. Ditto, Alice Amell, Sarah E. Frick, Monica Gamez-Djokic, Rebecca Hofstein Grady, Jesse Graham, Jun Gu, Adam Hahn, Brittany E. Hanson, Nicole J. Hartwich, Kristie Hein, Yoel Inbar, Lily Jiang, Tehlyr Kellogg, Nicole Legate, Timo P. Luoma, Heidi Maibeucher, Peter Meindl, Jennifer Miles, Alexandra Mislin, Daniel C. Molden, Matt Motyl, George Newman, Hoai Huong Ngo, Harvey Packham, P. Scott Ramsay, Jennifer L. Ray, Aaron M. Sackett, Anne-Laure Sellier, Tatiana Sokolova, Walter Sowden, Daniel Storage, Xiaomin Sun, Jay J. Van Bavel, Anthony N. Washburn, Cong Wei, Erik Wetter, Carlos T. Wilson, Sophie-Charlotte Darroux, Eric Luis Uhlmann

**Affiliations:** 1 INSEAD, Fontainebleau 77305, France and Singapore 138676, Singapore; 2 IMD, Lausanne, Lausanne 1001, Switzerland; 3 University of Washington Bothell, Bothell 98011, USA; 4 IE Business School, IE University, Madrid 28006, Spain; 5 HEC Paris, Jouy-en-Josas 78351, France; 6 University of Padova, Padova 35131, Italy; 7 University of Washington, Seattle 98195, USA; 8 University of Manitoba, Winnipeg R3T 5V4, Canada; 9 University of Chicago, Chicago 60637, USA; 10 University of Michigan, Ann Arbor 48109, USA; 11 Harvard University, Cambridge 2138, USA; 12 University of Utah, Salt Lake City 84112, USA; 13 Yale University, New Haven 6511, USA; 14 University of Missouri, Columbia 65211, USA; 15 Rotterdam School of Management, Erasmus University, Rotterdam 3000 DR, The Netherlands; 16 University of Amsterdam, Amsterdam 1001 NK, The Netherlands; 17 UCP—Católica Lisbon School of Business & Economics, Lisbon 1649-023, Portugal; 18 Hang Seng Management College, Hong Kong, Hong Kong; 19 Roosevelt University, Chicago 60605, USA; 20 University of Illinois at Urbana-Champaign, Champaign 61820, USA; 21 Illinois Institute of Technology, Chicago 60616, USA; 22 University of California, Irvine 92697, USA; 23 University of South Florida, Tampa 33620, USA; 24 Institute for Social Research, University of Michigan, Ann Arbor 48104, USA; 25 University of Massachusetts Amherst, Amherst 1003, USA; 26 Washington University in St Louis, St Louis 63130, USA; 27 University of Hong Kong, Hong Kong, Hong Kong; 28 Department of Psychology, New York University, New York 10003, USA; 29 American University, Washington 20016, USA; 30 Northwestern University, Evanston 60208, USA; 31 University of Southern California, Los Angeles 90089, USA; 32 Monash University, Melbourne 3145, Australia; 33 Social Cognition Center Cologne, University of Cologne, Koeln 50931, Germany; 34 University of Illinois at Chicago, Chicago 60607, USA; 35 University of Toronto, Toronto ON M5S, Canada; 36 University of Pennsylvania, Philadelphia 19104, USA; 37 Université Paris Ouest Nanterre La Défense, Nanterre 92000, France; 38 University of St Thomas, St Paul 55105, USA; 39 Centre for Psychiatry and Neuroscience, Walter Reed Army Institute of Research (WRAIR), Silver Spring 20910, USA; 40 Beijing Normal University, Beijing 100875, China; 41 Stockholm School of Economics, Stockholm 11383, Sweden

**Keywords:** Ethics, Decision making, Psychology, Research management, Ethics

## Abstract

We present the data from a crowdsourced project seeking to replicate findings in independent laboratories before (rather than after) they are published. In this Pre-Publication Independent Replication (PPIR) initiative, 25 research groups attempted to replicate 10 moral judgment effects from a single laboratory’s research pipeline of unpublished findings. The 10 effects were investigated using online/lab surveys containing psychological manipulations (vignettes) followed by questionnaires. Results revealed a mix of reliable, unreliable, and culturally moderated findings. Unlike any previous replication project, this dataset includes the data from not only the replications but also from the original studies, creating a unique corpus that researchers can use to better understand reproducibility and irreproducibility in science.

## Background & Summary

The replicability of findings from scientific research has garnered enormous popular and academic attention in recent years^1–3^. Results of replication initiatives attempting to reproduce previously published findings reveal that the majority of independent studies do not produce the same significant effects as the original investigation^1–5^.

There are many reasons why a scientific study may fail to replicate besides the original finding representing a false positive due to publication bias, questionable research practices, or error. These include meaningful population differences between the original and replication samples (e.g., cultural, subcultural, and demographic variability), overly optimistic estimates of study power based on initially published results, study materials that were carefully pre-tested in the original population but are not as well suited to the replication sample, a lack of replicator expertise, and errors in how the replication was carried out. Nonetheless, the low reproducibility rate has contributed to a crisis of confidence in science, in which the truth value of even many well-established findings has suddenly been called into question^6^.

The present line of research introduces a collaborative approach to increasing the robustness and reliability of scientific research, in which findings are replicated in independent laboratories *before*, rather than after, they are published^7,8^. In the Pre-Publication Independent Replication (PPIR) approach, original authors volunteer their own findings and select expert replication labs with subject populations they expect to show the effect. PPIR increases the informational value of unsuccessful replications, since common alternative explanations for failures to replicate such as a lack of replicator expertise and theoretically anticipated population differences are addressed. Sample sizes are also much larger than is common in the field, and the analysis plan is pre-registered^9^, allowing for more accurate effect size estimates and identification of unexpected population differences. An effect has been overestimated and is quite possibly a false positive if it consistently fails to replicate in PPIRs. Pre-publication independent replication also has the benefit of ensuring published findings are reliable before they are widely disseminated, rather than only checking after-the-fact.

In this first crowdsourced Pre-Publication Independent Replication (PPIR) initiative, 25 laboratories attempted to replicate 10 unpublished moral judgment findings in the research ‘pipeline’ of the last author and his collaborators (see Table 1). The original authors selected replication laboratories with directly relevant expertise (e.g., moral judgment researchers), and access to subject populations theoretically expected to show the effect. A pre-set list of replication criteria were applied^10^: whether the original and replication effect were in the same direction, whether the replication effect was statistically significant, whether the effect size was significant meta-analyzing the original and replication studies, whether the original effect size was within the confidence interval of the replication effect size, and finally the small telescopes criterion (a replication effect size large enough to be reliably captured by the original study^11^). Of the 10 original findings, 6 replicated according to all criteria, two studies failed to replicate entirely, one study replicated but with a smaller effect size than the original study, and one study replicated in United States samples but not outside the United States (see ref. 7 for a full empirical report).

Unique among the replication initiatives thus far, the pipeline project corpus includes the data from not only the replications but also all of the original studies targeted for replication. This creates a unique opportunity for future analysts to better understand reproducibility and irreproducibility in science, since the data from the original studies can be reanalyzed to better understand why a particular effect did or did not prove reliable. The dataset is complemented by both socioeconomic and demographic information on the research participants, and contains data from 6 countries (the United States, Canada, the Netherlands, France, Germany, and China) and replications in 4 languages (English, French, German, and Chinese). The Pre-Publication Independent Replication Project dataset is publicly available on the Open Science Framework (Data Citation 1) and is accompanied by SPSS syntax which can be used to reproduce the analyses. This array of data will serve as a resource for researchers interested in research reproducibility, statistics, population differences, culturally-moderated phenomena, meta-science, moral judgments, and the complexities of replicating studies. For example, the data can be re-analyzed using meta-regression techniques in order to better understand if certain study characteristics or demographics moderate effect sizes. A re-analyst could also try out different analytic techniques and see how robust certain effects are to different specifications.

## Methods

### Participants

The Pre-Publication Independent Replication Project corpus includes three datasets. The first dataset (PPIR 1.sav: Data Citation 1) contains data from 3 original studies and their replications, a second dataset (PPIR 2.sav: Data Citation 1) contains data from 3 original studies and their replications, and a third dataset (PPIR 3.sav: Data Citation 1) contains data from 4 original studies and their replications. In total data were collected from 11,805 participants. The first SPSS file (PPIR 1.sav: Data Citation 1) contains data from 3,944 participants (including 514 from the original studies), while the second SPSS file (PPIR 2.sav: Data Citation 1) contains data from 3,919 participants (including 351 from original studies) and the final SPSS file (PPIR 3.sav: Data Citation 1) contains data from 3,829 participants (including 582 from original studies). An additional replication dataset collected in France contained 113 participants. No participants were removed from either the original or replication studies. All participants agreed to the informed consent form and the studies were in accordance with ethics regulations of the respective universities.

### Testing procedure

The data were collected using both online and paper-pencil surveys from the respective laboratories and participants. The replications used the same materials and measurements as in the original studies, with the exception that the materials were translated into multiple languages. In the online version of the replications, Qualtrics was used to collect the data. This online platform allowed us to randomize the order by which the studies were presented. In order to prevent participant fatigue, studies were administered in one of three batches, each batch contained three to four studies, and study order was counterbalanced between subjects. Once subjects agreed to participate in the study, they read vignettes (see below for an example of the vignette from the Cold-Hearted Prosociality study) and completed survey questions assessing their reactions. Thereafter, the participants were thanked for their participation and debriefed.

*Karen works as an assistant in a medical center that does cancer research. The laboratory develops drugs that improve survival rates for people stricken with breast cancer. As part of Karen’s job, she places mice in a special cage, and then exposes them to radiation in order to give them tumors. Once the mice develop tumors, it is Karen’s job to give them injections of experimental cancer drugs.*

*Lisa works as an assistant at a store for expensive pets. The store sells pet gerbils to wealthy individuals and families. As part of Lisa’s job, she places gerbils in a special bathtub, and then exposes them to a grooming shampoo in order to make sure they look nice for the customers. Once the gerbils are groomed, it is Lisa’s job to tie a bow on them*.

Although the majority of the data were collected as described above, there were some exceptions. Specifically, as opposed to counterbalancing the order in which the study was presented, participants at Northwestern University were randomly allocated to a survey which either contained one longer study or three shorter studies that were presented in a fixed order. Participants at Yale University did not complete one study as the researchers felt that the participants may be offended by it. Also, there was a translation error in one study run at the INSEAD Paris laboratory which required that study to be re-run separately. Finally, study order for participants at HEC Paris was not counterbalanced but rather fixed. Table 2 includes an outline of the number of replications and conditions, a brief synopsis of study and instructions for creating variables. Detailed reports of each original study and the complete replication materials are available on the OSF in the Supplementary File 1 (00.Supplemental_Materials_Pipeline_Project_Final_10_24_2015.pdf: Data Citation 1). Supplementary File 2 outlines all the names and measurement details used in the study (PPIR _Codebook.xlsx: Data Citation 1).

## Data Records

All data records listed in this section are available from the Open Science Framework (Data Citation 1) and can be downloaded without an OSF account. The datasets were anonymized to remove any information that could identify the participant responses, such as identification numbers from Amazon’s Mechanical Turk. The analysis was conducted with SPSS version 20 and detailed SPSS syntax (including comments) are provided to help with data analysis. In total there are 3 datasets and 11 syntax files available. These datasets are also accompanied by a codebook which describes the variables, the coding transformations necessary to replicate the analyses, and a synopsis of the respective studies.

### First dataset

Location: (PPIR 1.sav: Data Citation 1)

File format: SPSS Statistic Data Document file (.sav)

This file contains basic demographic information and responses to the items measured in the Moral Inversion study (SPSS Syntax files/PPIR 1–2 moral inversion.sps: Data Citation 1), Intuitive Economics study (SPSS Syntax files/PPIR 1–4 intuitive economics.sps: Data Citation 1), and Burn in Hell study (SPSS Syntax files/PPIR 1–7 burn in hell.sps: Data Citation 1).

### Second dataset

Location: (PPIR 2.sav: Data Citation 1)

File format: SPSS Statistic Data Document file (.sav)

This file contains basic demographic information and responses to the items measured in the Presumption of Guilt study (SPSS Syntax files/PPIR 2—1 presumption of guilt.sps: Data Citation 1), The Moral Cliff study (SPSS Syntax files/PPIR 2–3 moral cliff.sps: Data Citation 1), and Bad Tipper study (SPSS Syntax files/PPIR 2–9 bad tipper.sps: Data Citation 1).

### Third dataset

Location: (PPIR 3.sav: Data Citation 1)

File format: SPSS Statistic Data Document file (.sav)

This file contains basic demographic information and responses to the items measured in the Higher Standard Effect study (SPSS Syntax files/PPIR 3–5 higher standard—Charity.sps: Data Citation 1; SPSS Syntax files/PPIR 3–5 higher standard—Company.sps: Data Citation 1), Cold Hearted Prosociality study (SPSS Syntax files/PPIR 3–6 cold-hearted.sps: Data Citation 1), Bigot-Misanthrope study (SPSS Syntax files/PPIR 3–8 bigot misanthrope.sps: Data Citation 1), and Belief-Act Inconsistency study (SPSS Syntax files/PPIR 3–10 belief-act inconsistency.sps: Data Citation 1).

### Codebook

Location: (Data descriptor—Codebook/PPIR _Codebook.xlsx: Data Citation 1)

File format: Microsoft Excel Worksheet (.xlsx)

Introduction to PPIR project, outline of transformations, descriptions and labels for variables from three datasets.

## Technical Validation

**Figure 1:**
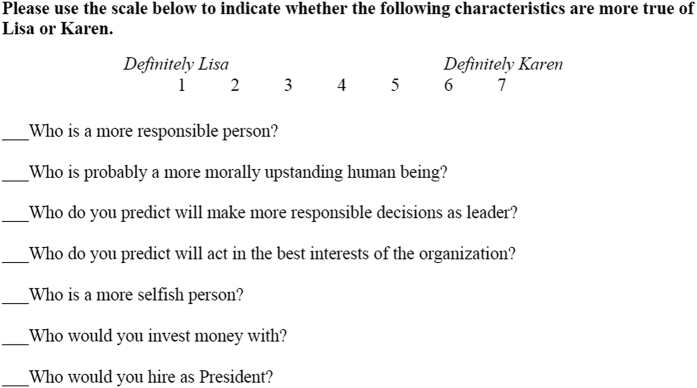
Example of the items measuring a typical moral judgement effect - in this instance candidate evaluations in the Higher Standards study. Figure 1 outlines a typical questionnaire that was administered to the subjects to assess their attitudes and beliefs toward the characters depicted in the vignettes. The subjects were required to write next to the statement the number that best indicated how much they believed the statement was representative of Lisa’s or Karen’s characteristics.

The studies include an array of original measurements which must be calculated to test the concepts of interest. These measures range from a single item to aggregated measures with multiple items, some of which must be reverse coded. (See Fig. 1, for an example of the items measuring candidate evaluations in the Higher Standards study; note that item 5 must be reverse coded prior to averaging the items into a composite). Instructions for how to create the study variables, the relevant conditions, and a synopsis of what concepts the variables measure, can be found in Table 2.

Figure 1 outlines a typical questionnaire that was administered to the subjects to assess their attitudes and beliefs toward the characters depicted in the vignettes. The subjects were required to write next to the statement the number that best indicated how much they believed the statement was representative of Lisa’s or Karen’s characteristics.

## Additional Information

**How to cite this article:** Tierney, W. *et al.* Data from a pre-publication independent replication initiative examining ten moral judgement effects. *Sci. Data* 3:160082 doi: 10.1038/sdata.2016.82 (2016).

## Supplementary Material

Supplementary File 1

Supplementary File 2

## Figures and Tables

**Table 1:** Overview of Replications.

**Data Citation**	**Study name**	**Number of replications**	**Participants**	
			**Original**	**Replication**
(PPIR 1.sav: Data Citation 1)	Moral Inversion	14	130 participants from Amazon's Mechanical Turk (MTurk)	3,133 participants
(PPIR 1.sav: Data Citation 1)	Intuitive Economics	16	226 students at Northwestern University	3,192 participants
(PPIR 1.sav: Data Citation 1)	Burn in Hell	16	158 students at Yale University (45%) and public campus areas at Northwestern University (55%)	3,430 participants
(PPIR 2.sav: Data Citation 1)	Presumption of Guilt	17	158 Northwestern undergraduates	3,820 participants
(PPIR 2.sav: Data Citation 1)	The Moral Cliff	15	114 participants from MTurk	3,592 participants
(PPIR 2.sav: Data Citation 1)	Bad Tipper	16	79 participants from MTurk	3,706 participants
(PPIR 3.sav: Data Citation 1)	Higher Standard Effect	11	265 participants from MTurk	2,888 participants
(PPIR 3.sav: Data Citation 1)	Cold Hearted Prosociality	12	79 participants from MTurk	3,016 participants
(PPIR 3.sav: Data Citation 1)	Bigot-Misanthrope	12	46 participants from MTurk	3,040 participants
(PPIR 3.sav: Data Citation 1)	Belief-Act Inconsistency	12	192 students at Northwestern University	3,708 participants

**Table 2:** Technical Validation and Study Synopsis.

**Data Citation**	**Study name**	**Synopsis of study**	**Study design**	**Number of conditions**	**Instructions for creating variables**
(PPIR 1.sav: Data Citation 1)	Moral Inversion	Moral Inversion Effect. A company that contributes to charity but then spends even more money promoting the contribution in advertisements not only nullifies its generous deed, but is perceived even more negatively than a company that makes no donation at all. Thus, even an objectively helpful act can provoke moral condemnation, so long as it suggests negative underlying traits such as insincerity.	Between Subjects	4	Evaluations: Participants reported on 9-point scales whether they viewed a company as untrustworthy-trustworthy and manipulative-not manipulative. They further provided their moral evaluations of the company on nine-point scales on the dimensions immoral-moral and bad-good.
(PPIR 1.sav: Data Citation 1)	Intuitive Economics	Intuitive Economics Effect. Economic variables that are widely regarded as unfair are perceived as especially bad for the economy. Such a correlation raises the possibility that moral concerns about fairness irrationally influence perceptions of economic processes. In other words, aspects of free markets that seem unfair on moral grounds (e.g., replacing hardworking factory workers with automated machinery that can do the job more cheaply) may be subject to distorted perceptions of their objective economic effects.	Between Subjects	2	Violations of fairness and economic consequences: Participants evaluated the 21 economic variables from the SAEE along two dimensions. Specifically, they indicated whether they viewed the economic variable as fair or unfair (1=very fair, 7=very unfair; *Note that in Condition 1 the scale endpoints are reversed, such that 1=very unfair and 7=very fair), and as good or bad for the economy (1=very bad for the economy, 7=very good for the economy).
(PPIR 1.sav: Data Citation 1)	Burn in Hell	Burn-in-Hell Effect. Participants perceive corporate executives as more likely to burn in hell than members of social categories defined by antisocial behavior, such as vandals. This reflects very negative assumptions about senior business leaders. ‘Vandals’ is a social category defined by bad behavior; ‘corporate executive’ is simply an organizational role. However, the assumed behaviors of a corporate executive appear negative enough to warrant moral censure.	Within Subjects	1	Participants estimated the percentage of individuals from a variety of social categories who would burn in hell (given that hell exists). The categories were: social workers, drug dealers, shoplifters, non-handicapped people who park in the handicapped spot, top executives at big corporations, people who sell prescription pain killers to addicts, people who kick their dog when they’ve had a bad day, car thieves, and vandals who spray graffiti on public property.
(PPIR 2.sav: Data Citation 1)	Presumption of Guilt	Presumption of Guilt Effect. For a company, failing to respond to accusations of misconduct leads to similar judgments as being investigated and found guilty. Companies accused of wrongdoing may be simply assumed to be guilty until proven otherwise. Inaction or ‘no comment’ responses to public accusations may be in effect an admission of guilt.	Between Subjects	4	Company evaluations: Participants evaluated the company on nine-point scales along the dimensions Bad-Good, Unethical-Ethical, Immoral-Moral, Irresponsible-Responsible, Deceitful-Honest, and Guilty-Innocent.
(PPIR 2.sav: Data Citation 1)	The Moral Cliff	Moral Cliff Effect. A company that airbrushes the model in their skin cream advertisement to make her skin look perfect is seen as more dishonest, ill-intentioned, and deserving of punishment than a company that hires a model whose skin already looks perfect. This reflects inferences about underlying intentions and traits. In both cases consumers have been equally misled by a perfect-looking model, but in the airbrushing case the deception seems more deliberate and explicitly dishonest.	Within and Between Subjects	2	Accuracy: Participants were asked how accurately the company's advertisement portrayed the effectiveness of their skin cream (1=extremely inaccurately 7=extremely accurately) and whether the ad created a correct impression regarding the product (1=extremely incorrect 7=extremely correct). Dishonesty. Three items asked whether the ad was dishonest (1=not at all dishonest, 7=extremely dishonest), fraudulent (1=not at all fraudulent, 7=extremely fraudulent), and a case of false advertising (1=definitely false advertising, 7=definitely truthful advertising; *Note that in all conditions this item is reverse scored). Punitiveness. Participants indicated whether the advertisement should be banned (1=definitely not, 7=definitely yes) and if the company should be fined for running the ad (1=definitely not, 7=definitely yes). Intentionality. An item asked if the company had intentionally misrepresented their product (1=definitely not, 7=definitely yes).
(PPIR 2.sav: Data Citation 1)	Bad Tipper Study	Bad Tipper Effect. A person who leaves the full tip entirely in pennies is judged more negatively than a person who leaves less money in bills, and tipping in pennies is seen as higher in informational value regarding character. This provides rare direct evidence of the role of perceived informational value regarding character in moral judgments. Moral reactions often track perceived character deficits rather than harmful consequences.	Between Subjects	2	Person judgments: To assess character-based judgments, participants were asked whether Jack was a disrespectful person, had a good moral conscience, was a good person, and was the type of person they would want as a friend (1=Not at all, 7=Definitely; *The items moral conscience, good person and close friend should be reverse scored such that higher scores indicate more negative person judgments).
(PPIR 3.sav: Data Citation 1)	Higher Standard Effect	Higher Standard Effect. It is perceived as acceptable for a private company to give small (but not large) perks to its top executive. But for the leader of a charitable organization, even a small perk is seen as moral transgression. Thus, under some conditions a praiseworthy reputation and laudable goals can actually hurt an organization, by leading it to be held to a higher moral standard.	Between Subjects	6	Candidate evaluations: After reading the scenario, participants were asked whether a series of characteristics was more true of Lisa or Karen (the two executives) on a scale ranging from 1 (definitely Lisa) to 7 (definitely Karen). Participants rated the candidates in terms of their responsibility, moral character, selfishness, and willingness to act in the best interests of the organization. In the company condition they further indicated who they would invest money with, and in the charity condition who they would donate money with. In all conditions they reported who they would prefer to see hired. Candidate evaluations along these dimensions were highly correlated and were averaged into a reliable composite (*Note that in all conditions the selfishness item is reverse scored).
(PPIR 3.sav: Data Citation 1)	Cold Hearted Prosociality	Cold-Hearted Prosociality Effect. A medical researcher who does experiments on animals is seen as engaging in more morally praiseworthy acts than a pet groomer, but also as a worse person. This effect emerges even in joint evaluation, with the two targets evaluated at the same time. Such act-person dissociations demonstrate that moral evaluations of acts and the agents who carry them out can diverge in systematic and predictable ways.	Between Subjects	2	Moral actions: Participants were asked ‘Whose actions make a greater moral contribution to the world?’, ‘Whose actions benefit society more?’, ‘Whose job is more morally praiseworthy?’, and ‘Whose job duties make a greater moral contribution to society?’ (1=definitely Karen, 7=definitely Lisa; *reverse coded in condition Lisa). Items were scored and aggregated so that lower numbers reflected viewing the medical research assistant’s actions as more praiseworthy. Moral traits. Participants also assessed who was more caring, cold-hearted, aggressive, and kind-hearted (1=definitely Karen, 7=definitely Lisa; *reverse coded in condition Karen—items 2 and 3). Items were scored and aggregated so that lower numbers reflected more positive trait attributions regarding the medical research assistant.
(PPIR 3.sav: Data Citation 1)	Bigot-Misanthrope	Bigot-Misanthrope Effect. Participants judge a manager who selectively mistreats racial minorities as a more blameworthy person than a manager who mistreats all of his employees. This supports the hypothesis that the informational value regarding character provided by patterns of behavior plays a more important role in moral judgments than aggregating harmful versus helpful acts.	Between Subjects	2	Person judgments: To assess character-based judgments, participants were asked whether John or Robert was the more immoral and blameworthy person on a single 7-point scale. Responses were coded so that lower numbers reflected relatively greater condemnation of the bigot’s moral character.
(PPIR 3.sav: Data Citation 1)	Belief-Act Inconsistency	Belief-Act Inconsistency Effect. An animal rights activist who is caught hunting is seen as an untrustworthy and bad person, even by participants who think hunting is morally acceptable. This reflects person centered morality: an act seen as morally permissible in-and-of itself nonetheless provokes moral opprobrium due to its inconsistency with the agent's stated beliefs.	Between Subjects	3	Moral blame: Participants were asked how morally blameworthy or morally praiseworthy they found Bob as a person on a Likert scale ranging from −5 (Extremely Blameworthy) to +5 (Extremely Praiseworthy).
