# Supplementary material for: Data from a pre-publication independent replication initiative examining ten moral judgement effects
Source: Sci Data. 2016 Oct 11;3:160082. doi: 10.1038/sdata.2016.82 (PMC5058337; doi:10.1038/sdata.2016.82)
Supplement: Supplementary File 1 [file sdata201682-s1.pdf]

ONLINE SUPPLEMENTS FOR THE PIPELINE PROJECT

Table of Contents:

Supplement 1: Details regarding replication samples **p. 2**

Supplement 2: Full reports of ten original studies targeted for replication **p. 8**

Supplement 3: Replication materials **p. 109**

Supplement 4: Pre-registered analysis plan **p. 146**

Supplement 5: Small telescopes figure **p. 150**

Supplement 6: Moderator analyses **p. 151**

SUPPLEMENT 1: DETAILS REGARDING REPLICATION SAMPLES

Table S1a

Replication Locations and Sample Sizes for Study Packet 1

Studies	University	Sample Size	Online/Lab	Type of subject population
Intuitive Economics, Burn in Hell, Moral Inversion	University of St. Thomas	131	Lab	Undergrads (Business)
	American University in Washington DC	111	Lab	Undergrads (Multiple majors)
	University of California Irvine	279	Lab	Undergrads (Psychology)
	Mechanical Turk sample	1038	Online	General population
	University of Illinois Urbana-Champaign	114	Online	Undergrads & Grad Students (Psychology)
	University of Cologne, Germany	305	Online	Undergrads & Gen. Pop
	Illinois Institute of Technology	127	Online	Undergrads (Psychology)
	INSEAD, France	237	Online	Undergrads & Grad Students (Multiple majors)
	University of Hong Kong, China	124	Online	Undergrads (Multiple majors)
	Harvard University	39	Online	General Population
	New York University	327	Lab	Undergrads (Multiple Majors)
	University of Michigan	100	Lab	Undergrads (Psychology)
	University of Southern California	251	Online	Gen. Pop (yourmorals.org)

Note. Study packet 1 included data from 3183 participants

Table S1b

Replication Locations and Sample Sizes for Study Packet 2

Studies	University	Sample Size	Online/Lab	Type of subject population
Moral Cliff, Bad Tipper, Presumption of Guilt	University of St. Thomas	131	Lab	Undergrads (Business)
	American University in Washington DC	108	Lab	Undergrads (Multiple majors)
	University of California Irvine	244	Lab	Undergrads & Grad students (Business)
	Mechanical Turk sample	1033	Online	General population
	University of Cologne, Germany	266	Online	Undergrads & Gen Pop
	Illinois Institute of Technology	123	Online	Undergrads (Psychology)
	INSEAD, France	236	Online	Undergrads & Grad students (Multiple majors)
	Harvard University	51	Online	General Population
	University of Washington (Foster)	115	Lab	Undergrads (Business)
	University of Groningen, the Netherlands	240	Lab	Undergrads & Grad students (Business)
	University of Washington	289	Lab	Undergrads & Grad students (Multiple Majors)
	Beijing Normal University, China	111	Lab	Undergrads (Psychology)
	University of Toronto, Canada	384	Lab	Undergrads (Psychology)
	University of South Florida	237	Online	Undergrads (Multiple Majors)

Note. Study packet 2 included data from 3568 participants

Table S1c

Replication Locations and Sample Sizes for Study Packet 3

Studies	University	Sample Size	Online/Lab	Type of subject population
Cold-Hearted Prosociality, Belief Act Inconsistency, Bigot-Misanthrope, Higher Standard	University of St. Thomas	131	Lab	Undergrads (Business)
	American University in Washington DC	108	Lab	Undergrads (Multiple majors)
	Mechanical Turk sample	1026	Online	Gen Pop
	University of Cologne, Germany	254	Online	Undergrads & Gen Pop
	INSEAD, France	243	Online	Undergrads & Grad students (Multiple majors)
	Harvard University	39	Online	Gen Pop
	University of Southern California	302	Online	Gen Pop (yourmorals.org)
	University of Washington Bothell	179	Online	Undergrads & Grad students (Business)
	University of Illinois at Chicago	605	Online	Undergrads & Grad students (Multiple Majors)
	University of Massachusetts Amherst	104	Lab	Undergrads (Multiple majors)
	INSEAD, France*	256	Lab	Undergrads & Grad students (Multiple majors)

Notes. Study packet 3 included data from 3247 participants. *Bigot-Misanthrope data was recollected due to an error in the French language version of the survey.

Table S1d

Unique Study Packet for HEC Paris

Studies	Sample Size	Online/Lab	Type of subject population
Bad Tipper, Burn in Hell, Belief Act Inconsistency, Bigot-Misanthrope, Cold-Hearted Prosociality, Presumption of Guilt	113	Online	Students (MBA)

Note. In the HEC Paris data collection studies were presented in fixed rather than counterbalanced order, in the order listed above.

Table S1e

Unique Study Packets for Yale University

Studies	Sample Size	Online/Lab	Type of subject population
Intuitive Economics, Moral Inversion	154	Online	General Population
Moral Cliff, Bad Tipper, Presumption of Guilt	158	Online	General Population
Cold-Hearted Prosociality, Belief Act Inconsistency, Bigot-Misanthrope, Higher Standard	161	Online	General Population

Table S1f

Unique Study Packets for Northwestern University

**Studies**	**Sample Size**	**Online/Lab**	**Type of subject population**
Intuitive Economics	93	Lab	Undergrads and Grad Students (multiple majors)
Presumption of Guilt, Belief Act Inconsistency, Burn in Hell	188	Lab	Undergrads and Grad Students (multiple majors)

Note. Presumption of Guilt, Belief Act Inconsistency and Burn in Hell appeared in fixed order as shown above.

SUPPLEMENT 2: FULL REPORTS OF TEN ORIGINAL STUDIES TARGETED FOR REPLICATION

Presumption of Guilt Study

(Heinze, Uhlmann, & Diermeier)

In this study, a company faced with accusations of manufacturing harmful products either 1) announced an outside investigation, 2) did not invite an independent investigation, 3) was found innocent, or 4) was found guilty. We hypothesized that inviting an outside investigation would signal good faith and thus evoke more positive company evaluations than no investigation (see Heinze, Uhlmann, & Diermeier, 2014), but less positive attitudes than a finding of innocence.

Company evaluations in response to no investigation vs. a finding of guilt were more difficult to anticipate. To the extent people are willing and able to withhold judgment of a company accused of misconduct, merely being accused should evoke more positive evaluations than a finding of guilt. However, to the extent perceptions of a company accused of misconduct are quite negative in nature, social perceivers may assume the accusations are valid and condemn the company equally in the no investigation condition and guilty condition.

Methods

Participants and Design

One hundred fifty eight Northwestern undergraduates (REPLICATION: 3820 participants) took part in the study, which used a 4 (independent investigation announced,

company found innocent, company found guilty, or no investigation) between-subjects design. Participants were recruited in a public area on campus and took part in the survey in return for a small cash payment ($2). Five participants were automatically excluded from the primary analyses because they did not complete the key dependent measure (company evaluations), leaving a useable sample of 153. Data were not analyzed until after data collection had terminated, and all conditions and measures are described below in full.

Materials and Procedure

Crisis scenario. Participants read an ostensive news story about the (fictitious) Locks Corporation, which was accused of using an unhealthy food additive called Gloactimate. The news story read as follows:

> Chicago, Ill., December 2, 2007 – The Locks Corporation, based in Rockford, Illinois, today was accused that several of their food products contain a substance known as Gloactimate, which may be harmful to people's health. Gloactimate is an additive in processed foods and is used to increase the shelf life of foods. A recent series of studies found that Gloactimate raises "bad" cholesterol, lowers "good" cholesterol, and increases risk for heart disease.

Company response. In the *independent investigation announced* condition, participants read the corporation had invited independent investigators into their nationwide locations to test their products. A bipartisan NGO, the Advanced Science Institute, had accepted the company's invitation. In the *company found innocent* and *company found guilty* conditions, the scientists from the Advanced Science Institute subsequently provided a finding of either

innocence or guilt. In the *no investigation* condition, no independent investigation was mentioned.

Company evaluations. First, participants evaluated the Locks corporation on nine-point scales along the dimensions Bad-Good, Unethical-Ethical, Immoral-Moral, Irresponsible-Responsible, Deceitful-Honest, and Guilty-Innocent ($\alpha = .93$) (REPLICATION: $\alpha = .96$).

Independent investigator evaluations. For exploratory purposes, participants were further asked about their perceptions of the independent investigators. On nine-point scales, they were asked whether when it came to detecting Gloactimate, an independent group of scientists from the Advanced Science Institute would be Untrustworthy- Trustworthy, Incompetent-Competent, Dishonest-Honest, Unskilled-Skilled, Unethical-Ethical, and Incapable-Capable. They further indicated their level of agreement (*1 = completely disagree, 9 = completely agree*), with the statements "I would trust an investigation done by an independent group of scientists from the Advanced Science Institute," "An independent group of scientists from the Advanced Science Institute would have the skills and knowledge necessary to conduct a competent investigation," "An independent group of scientists from the Advanced Science Institute would have the public interest at heart when investigating the Locks Corporation," "An independent group of scientists from the Advanced Science Institute would be corrupted by the Locks Corporation," and "The Locks Corporation would be able to hide evidence of Gloactimate in its products if a group of scientists conducted an independent investigation." (REPLICATION: these items were not included).

Comprehension check. To get a sense of whether participants understood the scenario properly, they were asked "Without looking back, what was the result of the investigation?" with

the options "company found innocent," "company found guilty," "independent investigation was announced but not yet executed," and "there were accusations but there had not yet been an independent investigation" provided. However, no subjects were removed from the analysis based on their response (REPLICATION: these items were not included).

Demographics. Finally, participants self-reported their gender, political orientation, and nation of origin. The complete study materials are provided at the end of this report.

Results and Discussion

There was a significant effect of experimental condition on company evaluations, $F(3, 149) = 24.40$, $p < .001$ (REPLICATION: $F(3, 3749) = 599.73$, $p < .001$, $\eta^2 = .32$). The company was viewed more positively when it announced an independent investigation than when there was no investigation (*M*s = 4.81 and 3.93, *SD*s = 1.39 and 1.27, respectively) (REPLICATION: investigation yes: $M = 5.29$; $SD = 1.85$ and investigation no: $M = 3.42$; $SD = 1.54$), $t(75) = 2.90$, $p = .005$ (REPLICATION: $t(3749) = 22.59$, $p < .001$), but less positively than when it was found innocent ($M = 6.36$, $SD = 1.52$), $t(77) = 4.75$, $p < .001$ (REPLICATION: investigation yes: $M = 5.29$; $SD = 1.85$ and innocent: $M = 6.44$; $SD = 1.94$, $t(3749)=13.85$, $p < .001$). Interestingly, the company was not evaluated any more positively in the no investigation condition ($M = 3.93$, $SD = 1.27$), than the guilty condition ($M = 3.97$, $SD = 1.42$), $t < 1$ (REPLICATION: the company was evaluated less positively in the no investigation condition than in the guilty condition; guilty condition values: $M = 3.70$; $SD = 1.80$, $t(3749) = 3.47$, $p = .001$).

In sum, inviting an independent investigation led to more positive attitudes toward the company than no investigation, but less positive attitudes than when the company was found innocent. Consistent with the idea that people's assumptions about companies accused of

misconduct are quite negative in nature, participants were equally likely to condemn the company in the no investigation condition and guilty condition. Participants may have simply assumed the accusations against the company that did not invite an investigation were valid.

References

Heinze, J., Uhlmann, E.L., & Diermeier, D. (2014). Unlikely allies: Credibility transfer during a corporate crisis. *Journal of Applied Social Psychology*, *44*, 392-397.

Study Materials

NO INVESTIGATION CONDITION:

Chicago, Ill., December 2, 2007 – The Locks Corporation, based in Rockford, Illinois, today was accused that several of their food products contain a substance known as Gloactimate, which may be harmful to people's health. Gloactimate is an additive in processed foods and is used to increase the shelf life of foods. A recent series of studies found that Gloactimate raises "bad" cholesterol, lowers "good" cholesterol, and increases risk for heart disease.

Corporate Response:

The Locks Corporation announced that it is confident in its adherence to government standards regarding Gloactimate.

INDEPENDENT INVESTIGATION ANNOUNCED CONDITION

Chicago, Ill., December 2, 2007 – The Locks Corporation, based in Rockford, Illinois, today was accused that several of their food products contain a substance known as Gloactimate, which may be harmful to people's health. Gloactimate is an additive in processed foods and is used to increase the shelf life of foods. A recent series of studies found that Gloactimate raises "bad" cholesterol, lowers "good" cholesterol, and increases risk for heart disease.

Corporate Response: The Company Allows an Independent Investigation

The Locks Corporation announced that it is confident in its adherence to government standards regarding Gloactimate and would allow independent investigators into any of their nationwide locations to test their products. The company emphasized that with food products in stores and warehouses throughout the country, there would be no feasible way the Gloactimate would go undetected.

An independent group of scientists from the Advanced Science Institute (ASI) has offered to conduct an independent investigation. ASI has formed a team of investigators that includes physicians, nutritionists, chemists, health inspectors and several senior members of ASI. The Locks Corporation has agreed to allow ASI access to any of its facilities.

COMPANY FOUND INNOCENT CONDITION

Chicago, Ill., December 2, 2007 – The Locks Corporation, based in Rockford, Illinois, today was accused that several of their food products contain a substance known as Gloactimate, which may be harmful to people's health. Gloactimate is an additive in processed foods and is used to increase the shelf life of foods. A recent series of studies found that Gloactimate raises "bad" cholesterol, lowers "good" cholesterol, and increases risk for heart disease.

Corporate Response: The Company Allows an Independent Investigation

The Locks Corporation announced that it is confident in its adherence to government standards regarding Gloactimate and would allow independent investigators into any of their nationwide locations to test their products. The company emphasized that with food products in stores and warehouses throughout the country, there would be no feasible way the Gloactimate would go undetected.

An independent group of scientists from the Advanced Science Institute (ASI) has conducted an independent investigation. ASI formed a team of investigators that included physicians, nutritionists, chemists, health inspectors and several senior members of ASI. The Locks Corporation agreed to allow ASI access into any of its facilities. This group of scientists has concluded that the food from the Locks Corporation **does not** contain Gloactimate.

COMPANY FOUND GUILTY CONDITION

Chicago, Ill., December 2, 2007 – The Locks Corporation, based in Rockford, Illinois, today was accused that several of their food products contain a substance known as Gloactimate, which may be harmful to people's health. Gloactimate is an additive in processed foods and is used to increase the shelf life of foods. A recent series of studies found that Gloactimate raises "bad" cholesterol, lowers "good" cholesterol, and increases risk for heart disease.

Corporate Response: The Company Allows an Independent Investigation

The Locks Corporation announced that it is confident in its adherence to government standards regarding Gloactimate and would allow independent investigators into any of their nationwide locations to test their products. The company emphasized that with food products in stores and warehouses throughout the country, there would be no feasible way the Gloactimate would go undetected.

An independent group of scientists from the Advanced Science Institute (ASI) has conducted an independent investigation. ASI formed a team of investigators that included physicians, nutritionists, chemists, health inspectors and several senior members of ASI. The Locks Corporation agreed to allow ASI access into any of its facilities. This group of scientists has concluded that the food from the Locks Corporation **does** contain Gloactimate.

DEPENDENT MEASURES

Now, please use the following questions to rate the Locks Corporation: *(Circle only one number for each rating)*:

Bad **Good**
1 --------- 2 --------- 3 --------- 4 ---------5 --------- 6 ---------7 --------- 8 --------- 9

Unethical **Ethical**
1 --------- 2 --------- 3 --------- 4 ---------5 --------- 6 ---------7 --------- 8 --------- 9

Immoral **Moral**
1 --------- 2 --------- 3 --------- 4 ---------5 --------- 6 ---------7 --------- 8 --------- 9

Irresponsible **Responsible**
1 --------- 2 --------- 3 --------- 4 ---------5 --------- 6 ---------7 --------- 8 --------- 9

Deceitful **Honest**
1 --------- 2 --------- 3 --------- 4 ---------5 --------- 6 ---------7 --------- 8 --------- 9

Guilty **Innocent**
1 --------- 2 --------- 3 --------- 4 ---------5 --------- 6 ---------7 --------- 8 --------- 9

When it comes to detecting Gloactimate, **an independent group of scientists** from the Advanced Science Institute would be:

Untrustworthy **Trustworthy**
1 --------- 2 --------- 3 --------- 4 ---------5 --------- 6 ---------7 --------- 8 --------- 9

Incompetent **Competent**
1 --------- 2 --------- 3 --------- 4 ---------5 --------- 6 ---------7 --------- 8 --------- 9

Dishonest **Honest**
1 --------- 2 --------- 3 --------- 4 ---------5 --------- 6 ---------7 --------- 8 --------- 9

Unskilled **Skilled**
1 --------- 2 --------- 3 --------- 4 ---------5 --------- 6 ---------7 --------- 8 --------- 9

Unethical **Ethical**
1 --------- 2 --------- 3 --------- 4 ---------5 --------- 6 ---------7 --------- 8 --------- 9

Incapable **Capable**
1 --------- 2 --------- 3 --------- 4 ---------5 --------- 6 ---------7 --------- 8 --------- 9

***Using the scale below, please indicate your agreement with the following statements*:**

1 --------- 2 --------- 3 --------- 4 ---------5 --------- 6 ---------7 --------- 8 --------- 9		
completely disagree	neither agree nor disagree	completely agree

____ I would trust an investigation done by an independent group of scientists from the Advanced Science Institute.

____An independent group of scientists from the Advanced Science Institute would have the skills and knowledge necessary to conduct a competent investigation.

____An independent group of scientists from the Advanced Science Institute would have the public interest at heart when investigating the Locks Corporation.

____An independent group of scientists from the Advanced Science Institute would be corrupted by the Locks Corporation.

____The Locks Corporation would be able to hide evidence of Gloactimate in its products if a group of scientists conducted an independent investigation.

Without looking back, what was the result of the investigation? *(PLEASE CIRCLE ONE)*

Company found innocent
Company found guilty
Independent investigation was announced but not yet executed
There were accusations but there had not yet been an independent investigation

Politically, I am *(PLEASE CIRCLE ONE)*

Very Liberal
Liberal
Somewhat Liberal
Moderate
Somewhat Conservative
Conservative
Very Conservative

My gender is (please circle one): Male Female

What is your nation of origin? ____________________

Moral Inversion Study

(Uhlmann, Tannenbaum, & Diermeier)

In 1999 Philip Morris donated $115 million to charities such as battered women's shelters and homeless shelters. That same year the tobacco company spent $150 million on its "Working to Make a Difference" advertising campaign to promote its charitable contributions. In one of the ads, a woman named Laura tells viewers "When I was 9 months pregnant, my husband beat me. But thanks to Philip Morris, one of the largest supporters of battered women's shelters, women (like me) and children are starting new lives." After the ratio of dollars spent on actual contributions to that spent on touting the contributions became known, Philip Morris was widely attacked by mainstream media outlets. Likewise, representatives in the U.S. Congress denounced the company's "tremendous deceit" (Philip Morris's Charitable Giving, 2001, p. 1808). This cautionary tale shows that it is possible to spend a quarter of a billion dollars trying to improve your image, genuinely help numerous battered women, homeless families, and others in need, and be no better off than when you started. In fact, you could even be worse off.

The "Working to Make a Difference" advertising campaign highlights the destructive effects of perceived ulterior motives for prosocial acts on one's social reputation. However, it is unclear how people would react to a less disreputable company broadcasting its charitable acts. It also remains an empirical question whether Philip Morris would have been better off not donating to charity at all. True, the company engaged in a self-congratulatory advertising

campaign, but $115 million helped a great many needy people and perhaps the company received some credit for that.

This study tested the *moral inversion* hypothesis that charitable acts are nullified when companies spend more money promoting their donation activities than on the actual donation amount. The weak version of the moral inversion hypothesis predicts that self-promotion cancels out charitable acts; the strong version predicts that exploiting charitable acts is perceived even more negatively than making no charitable contribution at all.

Methods

One hundred thirty participants (64% female; $M_{age} = 34$) (REPLICATION: 3133 participants, 53.8% female, $M_{age} = 26.51$, $SD = 11.05$) were recruited from Amazon.com's Mechanical Turk (MTurk) service in return for a small cash payment. Participants were randomly assigned to one of four between-subjects conditions: *charity only*, *publicized charity*, *charity + furniture advertising*, or *no contribution*. Data were not analyzed until after data collection had terminated, no participants were excluded for any reason, and all conditions and dependent measures are described below in full.

Participants in the *charity only* condition read that Farrell Incorporated, a large home furnishing company, recently donated $200,000 to support research on cancer. In the *publicized charity* condition, Farrell Incorporated donated $200,000 to cancer research and subsequently spent $2 million publicizing its charitable contribution. In the *charity + furniture advertising* condition, the company donated $200,000 for cancer research and subsequently spent $2 million to advertise its furniture. In the *no contribution* condition, the company did not donate any money to charity (thus serving as a baseline/control condition).

After reading the scenario, participants reported on 9-point scales whether they viewed the company as untrustworthy–trustworthy and manipulative-not manipulative ($\alpha = .86$) (REPLICATION: $\alpha = .81$). They further provided their moral evaluations of Farrell Incorporated on nine-point scales on the dimensions immoral-moral and bad-good ($\alpha = .95$) (REPLICATION: $\alpha = .90$).

Comprehension check items asked "Did the company donate money to cancer research?" (*1 = Yes, 2= No*) and "Did the company also spend money on an advertising campaign about its donation for cancer research?" (*1 = Yes, 2= No*). However no participants were removed from analyses based on their responses to these items (REPLICATION: did not include these items).

Finally, we asked participants to report their age, political orientation (*1= very liberal, 7 = very conservative*), gender, and nationality.

These scenarios and questionnaire items are provided at the end of this study report. The original data collection occurred in 2009, and in 2014 we noticed three items of unclear origin in the datafile (labeled "friends" "sweater" and "taxes") that used a different scale (-3 to +3) from the moral evaluations and trust DVs, and more importantly were not in the word version of the materials we had on file. These items appear to have been added in at the last minute and then forgotten entirely.

Results and Discussion

Company evaluations. Evaluations of Farrell Incorporated differently significantly by experimental condition, $F(3, 125) = 22.91$, $p < .001$ (REPLICATION: $F(3, 3126) = 249.95$, $p <.001$). Participants evaluated the company more negatively in the publicized charity condition ($M = 3.31$, $SD = 1.54$) (REPLICATION: $M = 3.59$; $SD = 1.85$) than in the charity only condition

(*M* = 5.60, *SD* = 1.22) (REPLICATION: *M* = 5.75; *SD* = 1.66), $t(66) = 6.81$, $p < .001$ (REPLICATION: $t(3126) = 25.16$, $p < .001$, charity + furniture advertising condition (*M* = 5.34, *SD* = 1.26) (REPLICATION: *M* = 5.73; *SD* = 1.76), $t(69) = 6.09$, $p < .001$ (REPLICATION: $t(3126) = 21.86$, $p < .001$), and even the no charity condition (*M* = 4.33, *SD* = .90) (REPLICATION: *M* 5.23; *SD* = 1.35), $t(56) = 2.92$, $p = .005$ (REPLICATION: $t(3126) = 10.34$, $p < .001$). Furthermore, the company was evaluated similarly in the charity only and charity + furniture advertising conditions, $t < 1$. The latter finding rules out the explanation that people dislike the company spending proportionally more money on something other than charitable contributions, since participants evaluated the charitable company positively even when it heavily advertised its furniture.

Trust in company. Feelings of trust in the company followed a similar pattern, $F(3, 124) = 27.08$, $p < .001$ (REPLICATION: $F(3, 3117) = 201.55$). The company was viewed as less trustworthy in the publicized charity condition (*M* = 2.76, *SD* = 1.36) (REPLICATION: *M* = 4.35; *SD* = 1.92) than in the charity only condition (*M* = 5.15, *SD* = 1.20) (REPLICATION: *M* = 6.35; *SD* = 1.59), $t(65) = 7.65$, $p < .001$ (REPLICATION: $t(3117) = 23.79$, $p < .001$), charity + furniture advertising condition (*M* = 5.11, *SD* = 1.42) (REPLICATION: *M* = 5.73; *SD* = 1.76), $t(68) = 7.04$, $p < .001$ (REPLICATION: $t(3117) = 16.32$, $p < .001$), as well as the no charity condition (*M* = 4.15, *SD* = .81) (REPLICATION: *M* = 5.23; *SD* = 1.35), $t(55) = 4.45$, $p < .001$ (REPLICATION: $t(3117) = 10.34$, $p < .001$).

In sum, a company that aggressively advertised its charitable acts not only squandered the good will it might have earned, but was judged even more harshly than a company that made no

charitable contribution at all. These findings therefore support the strong version of the moral inversion hypothesis.

Study Materials

NO CONTRIBUTION CONDITION

Farrell Incorporated is a multi-billion dollar home furnishing company.

CHARITY ONLY CONDITION

Farrell Incorporated is a multi-billion dollar home furnishing company.

Recently the company donated 200,000 dollars to a charity for cancer research.

PUBLICIZED CHARITY CONDITION

Farrell Incorporated is a multi-billion dollar home furnishing company.

Recently the company donated $200,000 dollars to a charity for cancer research.

The company then spent 2 million dollars on an advertising campaign about its donation for cancer research.

CHARITY + FURNITURE ADVERTISING CONDITION

Farrell Incorporated is a multi-billion dollar home furnishing company.

Recently the company donated 200,000 dollars to a charity for cancer research.

The company also spent 2 million dollars on an advertising campaign about its home furnishings.

DEPENDENT MEASURES

Farrell Incorporated is:

Manipulative NOT manipulative
1 --------- 2 --------- 3 --------- 4 ---------5 --------- 6 ---------7 --------- 8 --------- 9

Untrustworthy Trustworthy
1 --------- 2 --------- 3 --------- 4 ---------5 --------- 6 ---------7 --------- 8 --------- 9

Bad Good
1 --------- 2 --------- 3 --------- 4 ---------5 --------- 6 ---------7 --------- 8 --------- 9

Immoral Moral
1 --------- 2 --------- 3 --------- 4 ---------5 --------- 6 ---------7 --------- 8 --------- 9

Did the company donate money to cancer research?

Yes No

Did the company also spend money on an advertising campaign about its donation for cancer research?

Yes No

DEMOGRAPHICS

My age is: _______________

When it comes to politics I am *(please circle one):*

Very Liberal	Somewhat Conservative
Liberal	Conservative
Somewhat Liberal	Very Conservative
Moderate	

My gender is *(please circle one)*: Male Female

If not the USA, what country are you from? ____________

The Moral Cliff:

Understanding Leniency Towards Almost-Forbidden Behaviors

(Zhu & Uhlmann)

"The scandal isn't what's illegal, the scandal is what's legal"

-- Michael Kinsley

Consider the case of a scientist who runs a study, then deletes the 95% of the sample that failed to support the research hypothesis. Clearly this is scientific fraud. But what about the case of a scientist who runs 20 very similar studies, then reports only the one that worked? Not only is this is not legally fraud, it is not necessarily even grounds for a correction to the publication. Yet, the actual truth value of the published work would seem to be equally nil in the two cases.

The difference, it seems, lies not in objective truth value, but in the underlying intentions of the agent. The former agent knowingly acted nefariously; the latter could have engaged in psychological rationalizations but acted with legitimate scientific goals in mind (e.g., fine-tuning the experimental paradigm). The present research explored whether there is a "moral cliff" of unambiguously bad intentions beyond which agents are seen to condemn themselves irrevocably. Perhaps even more interestingly, just short of the cliff's edge behaviors that are in many respects just as objectively damaging can be treated with paradoxical leniency.

This initial study examined whether a moral cliff exists in the domain of false advertising. We tested the hypothesis that a cosmetics company that Photoshopped the model in its advertisement would be judged much more harshly than a company that simply hired a more

attractive model (eliminating the need to digitally enhance her appearance). The effectiveness of the cosmetics would seem to be equally misportrayed in the Photoshopped and non-Photoshopped advertisement. Yet only the digitally manipulated ad, we argue, stumbles across the moral cliff.

Methods

Participants and Design

One hundred and fourteen participants (REPLICATION: 3592, 55.1% female, M_{age} = 24.99, *SD* = 9.62) were recruited from Amazon.com's Mechanical Turk (MTurk) service and took part in the study in return for a small cash payment. The study employed a 2 (Photoshop vs. control) x 2 (counterbalancing order of the two scenarios) design, with the first factor manipulated within-subjects and the second factor between-subjects. Data were not analyzed until after data collection had terminated, no participants were excluded for any reason, and all conditions and dependent measures are described below in full.

Material and Procedures

Scenarios. All participants respond to the two target scenarios in counterbalanced order. In the *Photoshop scenario*, a cosmetics company hired a model to appear an advertisement for their skin cream. The model was one in a thousand in terms of the beauty of her skin. An artist who worked for the cosmetics company then used Photoshop to make her skin appear “one in a million.” In the *control scenario*, the company hired a model who already looked one in a million in terms of the beauty of her skin.

Accuracy. Participants were asked how accurately the company's advertisement portrayed the effectiveness of their skin cream (*1= extremely inaccurately 7= extremely accurately*) and

whether the ad created a correct impression regarding the product (*1= extremely incorrect 7= extremely correct*). These items formed a reliable index in both the control and Photoshop conditions ($\alpha_{Control}$ = .87 and $\alpha_{Photoshop}$ = .78) (REPLICATION: $\alpha_{Control}$ = .86 and $\alpha_{Photoshop}$ = .76).

Dishonesty. Three items asked whether the ad was dishonest (*1= not at all dishonest, 7 = extremely dishonest*), fraudulent (*1= not at all fraudulent, 7 = extremely fraudulent*), and a case of false advertising (*1= definitely false advertising, 7 = definitely truthful advertising*) (reverse scored), ($\alpha_{Control}$ = .30 and $\alpha_{Photoshop}$ = .67) (REPLICATION: $\alpha_{Control}$ = .64 and $\alpha_{Photoshop}$ = .52). Due to the low reliability of this measure in the control condition, results for the dishonesty composite should be interpreted with some caution.

Punitiveness. Participants indicated whether the advertisement should be banned (*1= definitely not, 7 = definitely yes*) and if the company should be fined for running the ad (*1= definitely not, 7 = definitely yes*) ($\alpha_{Control}$ = .92 and $\alpha_{Photoshop}$ = .93) (REPLICATION: $\alpha_{Control}$ = .87 and $\alpha_{Photoshop}$ = .88).

Intentionality. An item asked if the company had intentionally misrepresented their product (*1= definitely not, 7 = definitely yes*).

Rationalizability. A further item assessed how easy it was for the company to justify their behavior to themselves as legitimate (*1 = extremely difficult, 7 = extremely easy*). We had hoped this would form a reliable "bad faith" index with the intentionality item, but as responses to the two items were practically uncorrelated ($r_{Control}$ = -.04 and $r_{Photoshop}$ = -.11) (REPLICATION: $r_{Control}$ = -.38 $\alpha_{Control}$ = -.16 and $r_{Photoshop}$ = -.24 $\alpha_{Photoshop}$ = -.49), they were analyzed separately.

Comprehension check. For each scenario, participants were asked whether the company used Photoshop to make the model's skin look more beautiful (*Yes/No*). However, no participants were removed from the analyses based on their responses to this item.

Perceived base rates. For exploratory purposes, participants were asked what percentage of cosmetics companies they believed digitally manipulated the appearance of the models in their advertisements.

Demographic measures. Finally, participants reported their political orientation (*1 = very liberal, 7 = very conservative*), age, gender, ethnicity, country of birth, education level, occupation, and yearly income. The complete study measures are provided at the end of this report.

Results and Discussion

Given the design of the study, we conducted a two-way repeated measures ANOVA, with the first factor (Photoshop vs. control) within-subjects and the second factor (counterbalancing order of the two scenarios) between-subjects. We report results for each of our five dependent measures in turn.

Accuracy. Results indicated an unexpected significant difference between the Photoshop condition and the control condition in terms of the perceived accuracy of the advertisement, $F(1, 110) = 30.79$, $p < .001$, $\eta^2 = .22$ (REPLICATION: $F(1, 3535) = 163.82$, $p < .001$), such that participants evaluated the Photoshopped advertisement ($M_{\text{Photoshop}} = 2.32$, $SD = 1.37$) (REPLICATION: $M_{\text{Photoshop}} = 1.99$, $SD = 1.19$) as less accurate than the advertisement with an equally beautiful but non-Photoshopped model ($M_{\text{Control}} = 3.21$, $SD = 1.69$) (REPLICATION: $M_{\text{Photoshop}} = 2.86$, $SD = 1.55$). This was contrary to our expectation that participants would

acknowledge the equally low informational value of the two advertisements. Also unexpectedly, this effect was qualified by a significant interaction between Photoshop condition and the order in which the scenarios were presented, $F(1, 110) = 10.50, p = .008, \eta^2 = .06$ (REPLICATION: $F(1, 3535) = 198.60, p < .001$). Participants judged the advertisement in the control condition as significantly more accurate than its counterpart regardless of counterbalancing order. However, the effect was comparatively stronger when the Photoshop scenario preceded the control scenario ($M_{\text{PhotoshopFirst}} = 2.48, SD = 1.35$, vs. $M_{\text{ControlSecond}} = 3.80, SD = 1.64$) (REPLICATION: $M_{\text{PhotoshopFirst}} = 2.07, SD = 1.11$, vs. $M_{\text{ControlSecond}} = 3.28, SD = 1.63$), $t(55) = 5.00, p < .001$ (REPLICATION: $t(1764) = 31.74, p < .001$), as opposed to coming after it ($M_{\text{PhotoshopSecond}} = 2.16, SD = 1.39$ vs. $M_{\text{ControlFirst}} = 2.62, SD = 1.53$) (REPLICATION: $M_{\text{PhotoshopSecond}} = 1.92, SD = 1.26$ vs. $M_{\text{ControlFirst}} = 2.45, SD = 1.35$), $t(55) = 2.52, p = .015$ (REPLICATION: $t(1771) = 17.98, p < .001$).

Dishonesty. The expected significant difference emerged between the Photoshop and control condition with regards to the perceived honesty of the ad, $F(1, 105) = 49.01, p < .001, \eta^2 = .32$ (REPLICATION: $F(1, 3467) = 135.65, p < .001$). Using Photoshop led participants to evaluate the advertisement as more dishonest ($M_{\text{Photoshop}} = 5.07, SD = 1.36$) (REPLICATION: = 5.35, $SD = 1.22$) than the control ad ($M_{\text{Control}} = 4.14, SD = 1.26$) (REPLICATION: $M_{\text{Control}} = 4.44$, $SD = 1.32$), an effect that was not qualified by scenario order, $F(1, 105) = 2.41, p = .12$ (REPLICATION: effect that was qualified by scenario order: $F(1, 3467) = 83.43, p < .001$).

Punishment. As hypothesized, participants were more punitive toward the skin cream company if their advertisement used Photoshop ($M_{\text{Photoshop}} = 4.28, SD = 1.90$; $M_{\text{Control}} = 3.18, SD = 1.89$) (REPLICATION: $M_{\text{Photoshop}} = 4.42, SD = 1.78$; $M_{\text{Control}} = 3.26, SD = 1.65$), $F(1, 104) = 53.14, p < .001, \eta^2 = .34$ (REPLICATION: $F(1, 3461) = 1848.33, p < .001$). A marginally

significant interaction between Photoshop condition and counterbalancing order further emerged, $F(1, 104) = 3.40$, $p = .07$, $\eta^2 = .03$ (REPLICATION: $F(1, 3461) = 6.03$, $p < .001$). The effect was marginally stronger when the Photoshop condition came first ($M_{PhotoshopFirst} = 4.00$, $SD = 1.93$ vs. $M_{ControlSecond} = 2.63$, $SD = 1.73$ (REPLICATION: $M_{PhotoshopFirst} = 4.57$, $SD = 1.83$ vs. $M_{ControlSecond} = 3.04$, $SD = 1.64$), $t(53) = 6.16$, $p < .001$ (REPLICATION: $t(1724) = -30.38$, $p < .001$), rather than second ($M_{PhotoshopSecond} = 4.58$, $SD = 1.84$ vs. $M_{ControlFirst} = 3.76$, $SD = 1.89$), $t(51) = 4.08$, $p < .001$ (REPLICATION: $M_{PhotoshopSecond} = 4.57$, $SD = 1.83$ vs. $M_{ControlFirst} = 3.47$, $SD = 1.63$), $t(1724) = 30.49$, $p < .001$.

Intention to misrepresent. Participants perceived greater intent to misrepresent the product if the company used Photoshop ($M_{Photoshop} = 5.59$, $SD = 1.59$ vs. $M_{Control} = 4.42$, $SD = 1.92$) (REPLICATION: $M_{Photoshop} = 5.88$, $SD = 1.39$ vs. $M_{Control} = 4.80$, $SD = 1.78$), $F\ (1, 103) = 50.99$, $p < .001$, $\eta^2 = .33$ (REPLICATION: $F(1, 3525) = 1349.90$, $p < .001$). This was qualified by a significant interaction between Photoshop condition and counterbalancing order, $F(1, 103) = 9.90$, $p = .002$, $\eta^2 = .09$ (REPLICATION: $F(1, 1348.90) = 32.52$, $p < .001$). Again, a significant effect of Photoshop condition was observed regardless of counterbalancing order, but the effect was much stronger when the Photoshop scenario came first ($M_{PhotoshopFirst} = 5.28$, $SD = 1.60$ vs. $M_{ControlSecond} = 3.61$, $SD = 1.73$) (REPLICATION: $M_{PhotoshopFirst} = 5.74$, $SD = 1.40$ vs. $M_{ControlSecond} = 4.49$, $SD = 1.83$), $t(53) = 6.80$, $p < .001$ (REPLICATION: $t(1756) = 28.12$, $p < .001$), rather than second ($M_{PhotoshopSecond} = 5.92$, $SD = 1.52$ vs. $M_{ControlFirst} = 5.27$, $SD = 1.74$) (REPLICATION: $M_{PhotoshopSecond} = 6.01$, $SD = 1.37$ vs. $M_{ControlFirst} = 5.11$, $SD = 1.67$), $t(50) = 3.09$, $p = .003$ (REPLICATION: $t(1770) = 23.59$, $p < .001$). Although admittedly a post-hoc interpretation, the unanticipated interaction with scenario order across several outcome measures

could be a contrast effect, such that first being exposed to the Photoshop scenario makes the non-Photoshop scenario look better by comparison.

Rationalizability. Finally, participants perceived greater difficulty of rationalizing its behavior if the company used Photoshop ($M_{Photoshop}$ = 4.10, *SD* = 1.92 vs. $M_{Control}$ = 4.73, *SD* = 1.78) (REPLICATION: $M_{Photoshop}$ = 4.06, *SD* = 1.86 vs. $M_{Control}$ = 4.83, *SD* = 1.65), $F(1, 109) = 14.33$, $p < .001$, $\eta^2 = .12$ (REPLICATION: $F(1, 3545) = 806.22$, $p < .001$), an effect that was not qualified by scenario order, $F(1, 109) = .26$, $p = .61$ (REPLICATION: $F(1, 3545) =.60$, $p = .44$).

In sum, a company that digitally manipulated its advertisement was judged more harshly than a company that simply hired a more beautiful model. The Photoshopped ad was perceived as guided by a deliberate intent to deceive, as fraudulent, and grounds for punishing the company through fines and a ban on its advertisement. Contrary to predictions, participants did not even acknowledge that hiring a model who already had perfect skin portrayed the effectiveness of the skin cream just as inaccurately as digitally manipulating a model to appear to have perfect skin. Although speculative, this could be a case of belief overkill (Baron, 2009; Jervis, 1976) or moral coherence (Liu & Ditto, 2012), in which moral condemnation of the deceptive company distorted perceptions of their advertisement's objective truth value. Future studies will examine this possibility empirically, and test the moral cliff hypothesis in domains such as academic misconduct and accounting fraud.

References

Baron, J. (2009). Belief overkill in political judgments. *Informal Logic, 29*, 368-378.

Liu, B., & Ditto, P. H. (2012). What dilemma? Moral evaluation shapes factual belief. *Social Psychological and Personality Science, 4,* 316-323.

Jervis, R. (1976). *Perception and misperception in international politics*. Princeton: Princeton University Press.

Study Materials

NOTE: Participants respond to both scenarios in counterbalanced order, completing the same dependent measures twice.

PHOTOSHOP CONDITION

A cosmetics company hires a model to appear in an advertisement for their skin cream. She is one in a thousand in terms of the beauty of her skin. An artist who works for the cosmetics company then uses Photoshop to make her skin appear one in a million in terms of beauty. The skin cream advertisement with the model appears in magazines and on billboards all over the world.

CONTROL CONDITION

A cosmetics company hires a model to appear in an advertisement for their skin cream. She is one in a million in terms of the beauty of her skin. The skin cream advertisement with the model appears in magazines and on billboards all over the world.

DEPENDENT MEASURES

How accurately or inaccurately does the company's advertisement portray the effectiveness of their skin cream?

extremely inaccurately	1	2	3	4	5	6	7	extremely accurately

Does the company's advertisement create a correct impression of how well their skin cream works?

extremely incorrect	1	2	3	4	5	6	7	extremely correct

Is this advertisement dishonest?

not at all dishonest	1	2	3	4	5	6	7	extremely dishonest

Is this advertisement fraudulent?

not at all fraudulent	1	2	3	4	5	6	7	extremely fraudulent

Is this a case of false advertising?

Definitely false advertising	1	2	3	4	5	6	7	Definitely truthful advertising

Should this advertisement be banned?

Definitely not	1	2	3	4	5	6	7	Definitely yes

Should the company be fined money for running this ad?

Definitely not	1	2	3	4	5	6	7	Definitely yes

Did the company intentionally misrepresent their product to consumers?

Definitely not	1	2	3	4	5	6	7	Definitely yes

How easy or difficult is it for the company to justify their behavior to themselves as legitimate?

Extremely difficult	1	2	3	4	5	6	7	Extremely easy

In the scenario, did the company use Photoshop to make the model's skin look more beautiful?

Yes
No

What percentage of cosmetics companies do you think digitally manipulate their advertisements to make the models look better? __________%

DEMOGRAPHICS

My gender is *(please circle one)*: Male Female

My age is: _________

What country were you born in? ________________

My ethnicity is (please circle one): White Asian Latino Black
Other: _____________

My level of education is:

No high school degree
High school degree
Some college
College degree
Master's degree
Doctoral degree

My occupation is: _______________

My yearly income is: ____________

Politically, I am:

Very Liberal
Liberal
Somewhat Liberal
Moderate
Somewhat Conservative
Conservative
Very Conservative

Intuitive Economics Study

(Uhlmann & Diermeier)

This study examined whether concerns about unfairness predict the perceived material consequences of economic variables. Such a correlation would raise the possibility that people perceive certain economic variables as bad for the economy *because* they are unfair— in other words, that moral concerns distort logically unrelated perceptions of economic processes. Such a distortion effect with regards to economic beliefs would constitute an interesting case of the moral general phenomenon of moral coherence, in which factual beliefs shift to fall in line with moral evaluations (Liu & Ditto, 2012).

Notably, the Survey of Americans and Economists on the Economy (SAEE) reveals some interesting differences between laypeople and economists when it comes to perceived economic effects (Blendon et al., 1997; Caplan, 2001, 2002). For example, laypeople view high corporate salaries as a major source of economic problems, while economists do not. The perceived unfairness of corporate salaries and other economic variables was not assessed in the SAEE. However, this does raise the possibility that a belief that corporate salaries are unfair predicts the tendency to view them as bad for the economy. The present study measured both the perceived fairness and economic consequences of the variables from the SAEE to test for such correlations across a number of economic variables.

Methods

Participants and Design

226 students at Northwestern University (REPLICATION: 3192 participants) participated in the study. The study featured a correlation design with one between-subjects counterbalancing factor. Analyses were conducted only after all the data had been collected, no participants or conditions were excluded from analyses, and all measures are described below in full.

Materials and Procedure

Violations of fairness and economic consequences. Participants evaluated the 21 economic variables from the SAEE along two dimensions. Specifically, they indicated whether they viewed the economic variable as fair or unfair (*1 = very fair, 7 = very unfair*), and as good or bad for the economy (*1 = very bad for the economy, 7 = very good for the economy*). To control for potential response biases, for half of participants the unfairness item ranged from 1 (very fair) to 7 (very unfair) and for the other half of participants from 1 (very unfair) to 7 (very fair). Responses were recoded prior to analyses such that higher numbers reflected greater perceived fairness.

The 21 variables evaluated were: high taxes, the federal deficit, foreign aid, illegal immigration, tax breaks for business, welfare, affirmative action, people not valuing hard work, government regulation of business, people not saving their money, high business profits, the salaries of top corporate executives, a lack of business productivity, technology displacing workers, companies sending jobs overseas, companies downsizing, companies not investing in

education and job training, tax cuts, the entrance of women into the workforce, the increased use of technology in the workplace, and trade agreements between the U.S. and other countries.

Fairness independent of economic effects. We further attempted to address the fact that some participants may view an economic variable as unfair *because* of its negative effects on the economy. For example, a participant might reason that foreign aid saps resources and damages the overall U.S. economy, causing some Americans to unfairly lose their jobs. Perceiving a variable as unfair because it is bad for the economy is perfectly rational, but relatively uninteresting from a theoretical standpoint. Of greater interest is the possibility that some economic variables (e.g., high executive salaries) are perceived as bad for the economy *because they are unfair*. In other words, perceived violations of fairness may distort judgments of economic consequences. Therefore, for all 21 variables, participants were asked if their judgments of fairness were based on economic consequences, or a matter of principle and independent of any economic consequences (*1 = strongly disagree, 7 = strong agree*) (REPLICATION: these measures not included).

Demographics. Participants further reported demographic characteristics including political orientation (*1 = very liberal, 7 = very conservative*), gender, nationality, and the number of economics classes they had taken. The complete study materials are provided at the end of this report.

Results and Discussion

As expected, participants viewed variables that violated common sense notions of fairness (e.g., high corporate salaries) as bad for the economy. Indeed, as seen in column two of

the Table, the zero order correlation between perceived fairness and economic effects was significant for all 21 variables taken from the SAEE (REPLICATION: same result).

The causal influence could of course go in either direction— i.e., from perceived economic effects to fairness, or from perceived fairness to economic effects. Because our theoretical interest is in the latter possibility, in subsequent analyses for each variable all participants who indicated that their judgments of fairness were based on economic effects were removed from the sample. Only participants who indicated a 5, 6, or 7 on the relevant “in principle” item remained in the analysis (REPLICATION: this measure not included, so this analysis was not done). For these remaining participants, it is comparatively more likely that assessments of fairness distort perceived economic effects. Notably, even participants who met this criterion exhibited positive correlations between their assessments of fairness and economic effects (see Table, column 3).

Table 1

Economic Variable	Fairness-Economic Effects Correlation (All Participants)	Correlation ("independent of economic effects")
High taxes	.39** (N = 225)	.49** (N = 93)
The federal deficit	.39** (N = 224)	.26* (N = 59)
Foreign aid	.36** (N = 224)	.32** (N = 128)
Illegal immigration	.48** (N = 218)	.60** (N = 112)
Tax breaks for business	.56** (N = 223)	.62** (N = 83)
Welfare	.55** (N = 223)	.66** (N = 143)
Affirmative action	.60** (N = 223)	.58** (N = 128)
People not valuing hard work	.48** (N = 223)	.65** (N = 108)
Government regulation of business	.48** (N = 223)	.54** (N = 121)
People not saving their money	.18* (N = 222)	.23 (N = 71)
High business profits	.47** (N = 223)	.65** (N = 114)
The salaries of top corporate executives	.58** (N = 223)	.58** (N = 120)
A lack of business productivity	.36** (N = 221)	.48** (N = 58)
Technology displacing workers	.31** (N = 222)	.32* (N = 102)
Companies sending jobs overseas	.32** (N = 221)	.25* (N = 99)
Companies downsizing	.37** (N = 222)	.32** (N = 72)
Companies not investing in education and job training	.52** (N = 223)	.55** (N = 106)
Tax cuts	.61** (N = 223)	.70** (N = 114)
The entrance of women into the workforce	.39** (N = 221)	.32** (N = 181)
The increased use of technology in the workplace	.42** (N = 221)	.35** (N = 141)
Trade agreements between the U.S. and other countries	.45** (N = 222)	.57** (N = 125)

** $p < .001$, * $p < .05$

(REPLICATION:)

Economic Variable	Fairness-Economic Effects Correlation (All Participants)
1 High taxes	.49** (*N* = 3192)
2 The federal deficit	.48** (*N* = 3156)
3 Foreign aid	.43** (*N* = 3139)
4 The entrance of women into the workforce	.45** (*N* = 3139)
5 The increased use of technology in the workplace	.46** (*N* = 3134)
6 Trade agreements between the U.S. and other countries	.56** (*N* = 3142)
7 Companies downsizing	.34** (*N* = 3143)
8 Companies not investing in education and job training	.53** (*N* = 3130)
9 Tax cuts	.54** (*N* =3144)
10 A lack of business productivity	.35** (*N* = 3127)
11 Technology displacing workers	.37** (*N* = 3138)
12 Companies sending jobs overseas	.52** (*N* = 3133)
13 People not saving their money	.24** (*N* = 3143)
14 High business profits	.43** (*N* = 3134)
15 The salaries of top corporate executives	.63** (*N* = 3154)
16 Affirmative action	.70** (*N* = 3147)
17 People not valuing hard work	.43** (*N* = 3146)
18 Government regulation of business	.67** (*N* = 3134)
19 Illegal immigration	.65** (*N* = 3149)
20 Tax breaks for business	.62** (*N* = 3141)
21 Welfare	.58** (*N* = 3159)

One can also examine the link between assessments of unfairness and economic effects at the level of economic variable. In other words, one can correlate the extent to which each of the 21 economic variables was perceived as unfair on the one hand, and destructive to the economy on the other. This correlation was both statistically significant and high in absolute terms, $r(20) = .87$, $p < .001$ (REPLICATION: $r(20) = .90$, $p < .001$).

In sum, participants clearly viewed economic variables that violate common sense notions of fairness as also bad for the economy. This is consistent with the idea that perceived unfairness shapes assessments of economic effects, and more generally with the phenomenon of moral coherence (Liu & Ditto, 2012). However, the evidence from the present study is correlational and therefore cannot identify causal relationships.

References

Blendon, R.J., Benson, J.M., Brodie, M., Morin, R., Altman, D.E., Gitterman, G., Brossard, M., & James, M. (1997). Bridging the gap between the public's and economists' views of the economy. *Journal of Economic Perspectives, 11,* 105-118.

Caplan, B. (2001). What makes people think like economists? Evidence on economic cognition from the 'Survey of Americans and Economists on the Economy'. *Journal of Law and Economics, 43*, 395-426.

Caplan, B. (2002). Systematically biased beliefs about economics: Robust evidence of judgmental anomalies from the 'Survey of Americans and Economists on the Economy'. *Economic Journal, 112*, 433-458.

Liu, B., & Ditto, P. H. (2012). What dilemma? Moral evaluation shapes factual belief. *Social Psychological and Personality Science, 4,* 316-323.

Study Materials

Are high taxes fair or unfair?

Very FAIR			Neutral			Very UNFAIR
1	2	3	4	5	6	7

Are high taxes good or bad for the economy?

Very bad			Neither			Very good
1	2	3	4	5	6	7

High taxes are fair/unfair as a matter of principle (i.e, regardless of its effects on the overall economy).

Strongly Disagree			Neutral			Strongly Agree
1	2	3	4	5	6	7

Is the federal deficit fair or unfair?

Very FAIR			Neutral			Very UNFAIR
1	2	3	4	5	6	7

Is the federal deficit good or bad for the economy?

Very bad			Neither			Very good
1	2	3	4	5	6	7

The federal deficit is fair/unfair as a matter of principle (i.e, regardless of its effects on the overall economy).

Strongly Disagree			Neutral			Strongly Agree
1	2	3	4	5	6	7

Is foreign aid fair or unfair?

Very FAIR			Neutral			Very UNFAIR
1	2	3	4	5	6	7

Is foreign aid good or bad for the economy?

Very bad			Neither			Very good
1	2	3	4	5	6	7

Foreign aid is fair/unfair as a matter of principle (i.e, regardless of its effects on the overall economy).

Strongly Disagree			Neutral			Strongly Agree
1	2	3	4	5	6	7

Is the entrance of women into the workforce fair or unfair?

Very FAIR			Neutral			Very UNFAIR
1	2	3	4	5	6	7

Is the entrance of women into the workforce good or bad for the economy?

Very bad Neither Very good

1 2 3 4 5 6 7

The entrance of women into the workforce is fair/unfair as a matter of principle (i.e, regardless of its effects on the overall economy).

Strongly Disagree Neutral Strongly Agree

1 2 3 4 5 6 7

Is the increased use of technology in the workplace fair or unfair?

Very FAIR Neutral Very UNFAIR

1 2 3 4 5 6 7

Is the increased use of technology in the workplace good or bad for the economy?

Very bad Neither Very good

1 2 3 4 5 6 7

The increased use of technology in the workplace is fair/unfair as a matter of principle (i.e, regardless of its effects on the overall economy).

Strongly Disagree Neutral Strongly Agree

1 2 3 4 5 6 7

Are trade agreements between the U.S. and other countries fair or unfair?

Very FAIR Neutral Very UNFAIR

1 2 3 4 5 6 7

Are trade agreements between the U.S. and other countries good or bad for the economy?

Very bad Neither Very good

1 2 3 4 5 6 7

Trade agreements between the U.S. and other countries are fair/unfair as a matter of principle (i.e, regardless of its effects on the overall economy).

Strongly Disagree Neutral Strongly Agree

1 2 3 4 5 6 7

Is companies downsizing fair or unfair?

Very FAIR Neutral Very UNFAIR

1 2 3 4 5 6 7

Is companies downsizing good or bad for the economy?

Very bad Neither Very good

1 2 3 4 5 6 7

Companies downsizing is fair/unfair as a matter of principle (i.e, regardless of its effects on the overall economy).

Strongly Disagree Neutral Strongly Agree

1 2 3 4 5 6 7

Is companies not investing in education and job training fair or unfair?

Very FAIR Neutral Very UNFAIR

1 2 3 4 5 6 7

Is companies not investing in education and job training good or bad for the economy?

Very bad Neither Very good

1 2 3 4 5 6 7

Companies not investing in education and job training is fair/unfair as a matter of principle (i.e, regardless of its effects on the overall economy).

Strongly Disagree Neutral Strongly Agree

1 2 3 4 5 6 7

Are tax cuts fair or unfair?

Very FAIR Neutral Very UNFAIR

1 2 3 4 5 6 7

Are tax cuts good or bad for the economy?

Very bad Neither Very good

1 2 3 4 5 6 7

Tax cuts are fair/unfair as a matter of principle (i.e, regardless of its effects on the overall economy).

Strongly Disagree Neutral Strongly Agree

1 2 3 4 5 6 7

Is a lack of business productivity fair or unfair?

Very FAIR Neutral Very UNFAIR

1 2 3 4 5 6 7

Is a lack of business productivity good or bad for the economy?

Very bad Neither Very good

1 2 3 4 5 6 7

A lack of business productivity is fair/unfair as a matter of principle (i.e, regardless of its effects on the overall economy).

Strongly Disagree Neutral Strongly Agree

1 2 3 4 5 6 7

Is technology displacing workers fair or unfair?
Very FAIR Neutral Very UNFAIR
1 2 3 4 5 6 7

Is technology displacing workers good or bad for the economy?
Very bad Neither Very good
1 2 3 4 5 6 7

Technology displacing workers is fair/unfair as a matter of principle (i.e, regardless of its effects on the overall economy).
Strongly Disagree Neutral Strongly Agree
1 2 3 4 5 6 7

Is companies sending jobs overseas fair or unfair?
Very FAIR Neutral Very UNFAIR
1 2 3 4 5 6 7

Is companies sending jobs overseas good or bad for the economy?
Very bad Neither Very good
1 2 3 4 5 6 7

Companies sending jobs overseas is fair/unfair as a matter of principle (i.e, regardless of its effects on the overall economy).
Strongly Disagree Neutral Strongly Agree
1 2 3 4 5 6 7

Is people not saving their money fair or unfair?
Very FAIR Neutral Very UNFAIR
1 2 3 4 5 6 7

Is people not saving their money good or bad for the economy?
Very bad Neither Very good
1 2 3 4 5 6 7

People not saving their money is fair/unfair as a matter of principle (i.e, regardless of its effects on the overall economy).
Strongly Disagree Neutral Strongly Agree
1 2 3 4 5 6 7

Are high business profits fair or unfair?
Very FAIR Neutral Very UNFAIR
1 2 3 4 5 6 7

Are high business profits good or bad for the economy?
Very bad Neither Very good
1 2 3 4 5 6 7

High business profits are fair/unfair as a matter of principle (i.e, regardless of its effects on the overall economy).
Strongly Disagree Neutral Strongly Agree
1 2 3 4 5 6 7

Are the salaries of top (corporate) executives fair or unfair?
Very FAIR Neutral Very UNFAIR
1 2 3 4 5 6 7

Are the salaries of top (corporate) executives good or bad for the economy?
Very bad Neither Very good
1 2 3 4 5 6 7

The salaries of top (corporate) executives are fair/unfair as a matter of principle (i.e, regardless of its effects on the overall economy).
Strongly Disagree Neutral Strongly Agree
1 2 3 4 5 6 7

Is affirmative action fair or unfair?
Very FAIR Neutral Very UNFAIR
1 2 3 4 5 6 7

Is affirmative action good or bad for the economy?
Very bad Neither Very good
1 2 3 4 5 6 7

Affirmative action is fair/unfair as a matter of principle (i.e, regardless of its effects on the overall economy).
Strongly Disagree Neutral Strongly Agree
1 2 3 4 5 6 7

Is people not valuing hard work fair or unfair?
Very FAIR Neutral Very UNFAIR
1 2 3 4 5 6 7

Is people not valuing hard work good or bad for the economy?
Very bad Neither Very good
1 2 3 4 5 6 7

People not valuing hard work is fair/unfair as a matter of principle (i.e, regardless of its effects on the overall economy).
Strongly Disagree Neutral Strongly Agree
1 2 3 4 5 6 7

Is government regulation of business fair or unfair?
Very FAIR Neutral Very UNFAIR
1 2 3 4 5 6 7

Is government regulation of business good or bad for the economy?
Very bad Neither Very good
1 2 3 4 5 6 7

Government regulation of business is fair/unfair as a matter of principle (i.e, regardless of its effects on the overall economy).
Strongly Disagree Neutral Strongly Agree
1 2 3 4 5 6 7

Are illegal immigrants fair or unfair?
Very FAIR Neutral Very UNFAIR
1 2 3 4 5 6 7

Are illegal immigrants good or bad for the economy?
Very bad Neither Very good
1 2 3 4 5 6 7

Illegal immigrants are fair/unfair as a matter of principle (i.e, regardless of its effects on the overall economy).
Strongly Disagree Neutral Strongly Agree
1 2 3 4 5 6 7

Are tax breaks for business fair or unfair?
Very FAIR Neutral Very UNFAIR
1 2 3 4 5 6 7

Are tax breaks for business good or bad for the economy?
Very bad Neither Very good
1 2 3 4 5 6 7

Tax breaks for business are fair/unfair as a matter of principle (i.e, regardless of its effects on the overall economy).
Strongly Disagree Neutral Strongly Agree
1 2 3 4 5 6 7

Is welfare fair or unfair?

Very FAIR			Neutral			Very UNFAIR
1	2	3	4	5	6	7

Is welfare good or bad for the economy?

Very bad			Neither			Very good
1	2	3	4	5	6	7

Welfare is fair/unfair as a matter of principle (i.e, regardless of its effects on the overall economy).

Strongly Disagree			Neutral			Strongly Agree
1	2	3	4	5	6	7

Politically, I am *(please circle one):*

1 Very Liberal
2 Liberal
3 Somewhat Liberal
4 Moderate
5 Somewhat Conservative
6 Conservative
7 Very Conservative

My gender is *(please circle one)*: **1 Male** **2 Female**

What country are you from? ______________

Please list the approximate number of economics classes you have taken: ____________

Higher Standard Effect

(Srinivasan, Uhlmann, & Diermeier)

This study examined whether a positive reputation and laudable goals can cause an organization and its leader to be held to a higher standard, leading to more severe censure for moral transgressions. Specifically, even minor inappropriate expenses by the leader of a charity may be morally condemned and viewed as a violation of trust (Diermeier, 2011). Trust violations undermine the conviction the world is a just and orderly place and thus represent both a threat to the social order and a psychological threat (Koehler & Gershoff, 2003). We therefore investigated whether frivolous perks accorded to the leader of a charity would lead participants to feel the world is unstable, chaotic, and unfair.

Methods

Two hundred sixty five participants were recruited from Amazon.com's Mechanical Turk (MTurk) (REPLICATION: 2888 participants) service in return for a small cash payment. The study utilized a 2 (type of organization: charity or company) × 3 (requested compensation: small perk, large perk, or cash only) between subjects design. Data were not analyzed until after data collection had terminated, no participants were excluded from the analyses, and all conditions and dependent measures are described below in full.

Scenario. Participants read that an organization was deciding between two job candidates for a top management position. The two candidates, henceforth referred to the *target candidate* and *control candidate*, had comparable backgrounds and employment histories, and this information was counterbalanced across participants. The names of the candidates ("Lisa" and

"Karen"; two names equated for a number of connotations by Kasof, 1993) were also counterbalanced.

All candidates in all conditions requested compensation packages of the same total financial value. The only difference was that in some conditions, the target candidate requested a perk of a certain value as opposed making an equivalent salary request. In the *large perk* condition, the target candidate requested a chauffeured limousine on weekends. In the *small perk* condition, the target candidate requested expensive mineral water. We further manipulated the type of organization in question. In the *company condition*, the organization was called "The Jens Shoes Corporation." In the *charity condition,* the organization was called "Somalian Hunger Relief."

Candidate evaluations. After reading the scenario, participants were asked whether a series of characteristics was more true of Lisa or Karen on a scale ranging from 1 (*definitely Lisa*) to 7 (*definitely Karen*). Participants rated the candidates in terms of their responsibility, moral character, selfishness, and willingness to act in the best interests of the organization. In the company condition they further indicated who they would invest money with, and in the charity condition who they would donate money with. In all conditions they reported who they would prefer to see hired. These items were adapted from Tannenbaum, Uhlmann, & Diermeier (2011). Candidate evaluations along these dimensions were highly correlated and (after reverse scoring the selfishness item) were averaged into a reliable composite ($\alpha = .91$) (REPLICATION: $\alpha = .92$).

Informational value. Two items assessed the perceived informational value of each candidate's request (see also Tannenbaum et al., 2011). These items asked how much each

person's requested compensation "tell you about who she *really* is and what she is *really* like" (*1 = nothing, 7 = a great deal*) (REPLICATION: not included).

Evaluations of organization. Next participants were told to imagine that the organization had decided to hire the target candidate. They then evaluated the organization on seven-point scales on the dimensions bad-good, unfavorable-favorable, and negative-positive (α = .94) (REPLICATION: not included).

Trust in organization. On similar seven-point scales, participants further reported whether they felt the company was trustworthy, dependable, and reliable (α = .86) (REPLICATION: not included).

Betrayal. A further item read "I feel betrayed by the organization's choice for President" (*1 = strongly disagree, 7 =strongly agree*). We had originally intended for this betrayal item to be part of the trust in organization index, but it only correlated weakly (r = -.33) with the other items and was therefore analyzed separately. It is unclear whether the weak correlation is due to the betrayal item being more strongly worded that the other trust items, or negatively worded (REPLICATION: not included).

Petition item. A stand-alone item read "I would sign an online petition to display my support for the organization" (*1 = strongly disagree, 7 = strongly agree*) (REPLICATION: not included).

Social threat. Items adapted from Koehler and Gershoff's (2003) social threat measure asked participants whether each candidate being chosen would lead them to feel the world is an unfair, disorderly, and uncertain place (*1 = strongly disagree, 7 = strongly agree*). These

measures proved reliable for both the control candidate (α = .95) and target candidate (α = .94) (REPLICATION: not included).

Attention checks. Follow-up items asked participants if they had engaged in other activities during the survey and if they had read the instructions. However no participants were removed from the analyses based on their responses to the attention check items.

Demographics. Participants reported demographic characteristics including their age, political orientation, gender, and nationality.

Comprehension checks. Finally, participants were asked to recall whether the organization was a company or charity and whether a candidate had requested a perk. However no participants were removed from the analyses based on their responses.

The full study materials are provided at the end of this report.

Results and Discussion

Candidate evaluations. For ease of analysis and presentation, all candidate evaluation items were recoded such that positive scores reflected positive evaluations (and negative scores reflected negative evaluations) of the target candidate relative to the control candidate. An ANOVA revealed the hypothesized interaction between the type of organization (company vs. charity) and the target's compensation (cash only, large perk, or small perk) with regard to candidate evaluations $F(2, 255) = 3.50$, $p = .03$ (REPLICATION: did not reveal the hypothesized interaction, $F(2, 2748) = .65$, $p = .53$.)

When the candidates were contending for the leadership of the Jens Shoes Corporation, there was a significant effect of the target's requested compensation, $F(2, 134) = 9.07$, $p < .001$ (REPLICATION: $F(2, 1372) = 134.00$, $p < .001$). The target candidate was evaluated

significantly less positively when she requested a large perk ($M = 2.85$, $SD = 1.11$) (REPLICATION: $M = 3.14$; $SD = 1.05$) then when she requested only monetary compensation ($M = 3.94$, $SD = 1.25$) (REPLICATION: $M = 4.04$; $SD = .92$), $t(96) = 4.56$, $p <.001$ (REPLICATION: $t(917) = 13.71$, $p < .001$). However, the target was *not* evaluated significantly more negatively when she requested a small perk ($M = 3.47$, $SD = 1.47$) (REPLICATION: was evaluated more negatively $M = 3.03$; $SD = 1.08$) as opposed to monetary compensation $t(88) = 1.64$, $p = .11$ (REPLICATION: $t(912) = -15.72$, $p <.001$). The target candidate was also perceived significantly more positively in the small perk than in the large perk condition, $t(84) = 2.24$, $p = .03$ (REPLICATION: was not evaluated differently, $t(915) = -1.62$, $p =.11$).

There was also a significant effect of requested compensation when the candidates were contending for the leadership of Somalian Hunger Relief, $F(2, 121) = 7.29$, $p = .001$ (REPLICATION: $F(2, 1376) = 118.62$, $p <.001$). The target candidate was seen significantly less positively when she requested a perk rather than monetary compensation ($M = 4.25$, $SD = 1.29$) (REPLICATION: $M = 3.99$; $SD = .90$). In the case of the charity, this was true not only for the large perk condition ($M = 3.46$, $SD = 1.47$) (REPLICATION: $M = 3.03$; $SD = 1.09$), $t(82) = 2.54$, $p = .01$ (REPLICATION: $t(921) = 14.351$, $p < .001$), but even for the small perk condition ($M = 3.03$, $SD = 1.36$) (REPLICATION: $M = 3.03$; $SD = 1.26$), $t(73) = 3.95$, $p < .001$ (REPLICATION: $t(923) = 13.31$, $p < .001$). Moreover, when the candidates were competing for the leadership of Somalian Hunger Relief, there was no significant difference in candidate evaluations between the two perks conditions, $t(87) =1.40$, $p = .16$ (REPLICATION: $t(908) = .03$, $p = .98$).

Informational value. Since the control candidate's compensation did not vary by condition, our theoretical predictions were directed only at the perceived informational value of the target candidate's compensation. A 2 (company vs. charity) x 3 (cash only, large perk, or small perk) ANOVA revealed no significant organization type by compensation interaction with regard to the rated informativeness of the target candidate's pay request, $F(2, 258) = 1.26$, $p = .29$ (REPLICATION: not included). Only a significant main effect of compensation emerged, $F(2, 258) = 5.67$, $p = .004$ (REPLICATION: not included). The target candidate's pay request was seen as higher in informational value when she asked for a large perk ($M = 4.86$, $SD = 1.61$), $t(181) = 2.03$, $p = .044$ (REPLICATION: not included), or small perk ($M = 4.95$, $SD = 1.50$), $t(166) = 2.38$, $p = .018$ (REPLICATION: not included), relative to monetary compensation ($M = 4.39$, $SD = 1.54$) (REPLICATION: not included). Although as noted the hypothesized organization type by compensation interaction did not emerge, out of theoretical interest we examined the effects of the candidate's requested pay separately for the company and charity. However the main effect of pay did not reach significance separately for either the company, $F(2, 136) = 2.00$, $p = .14$, or the charity, $F(2, 122) = 2.56$, $p = .08$ (REPLICATION: not included).

Evaluations of organization. No interaction between organization type and compensation emerged with regards to evaluations of the company, $F(2, 254) = .40$, $p = .67$ (REPLICATION: not included). Despite the lack of a significant interaction, we examined the effects of candidate compensation separately for the company and charity out of theoretical interest. However, the same basic pattern emerged for both the Jens Shore Corporation and Somalian Hunger Relief. There was a significant effect of the compensation awarded by both the company, $F(2, 133) =$

4.83, $p = .009$ (REPLICATION: not included), and the charity, $F(2, 121) = 4.63$, $p = .01$ (REPLICATION: not included). The company was evaluated more negatively when it awarded a large perk ($M = 3.98$, $SD = 1.20$), $t(95) = 2.93$, $p = .004$ (REPLICATION: not included), or small perk ($M = 4.09$, $SD = 1.35$), $t(88) = 2.28$, $p = .025$ (REPLICATION: not included), relative to cash only ($M = 4.73$, $SD = 1.32$) (REPLICATION: not included). The charity was likewise assessed more negatively when it awarded a large perk ($M = 4.05$, $SD = 1.52$), $t(82) = 2.41$, $p = .018$ (REPLICATION: not included), or small perk ($M = 3.83$, $SD = 1.53$), $t(73) = 3.00$, $p = .004$ (REPLICATION: not included), relative to cash ($M = 4.81$, $SD = 1.28$) (REPLICATION: not included).

Trust in organization. The hypothesized interaction between type of organization and compensation did not reach statistical significance with regard to perceived trust, $F(2, 251) = 1.40$, $p = .25$ (REPLICATION: not included). However, further analyses revealed a potentially meaningful pattern. The compensation received by the leader of the Jens Shoes Corporation did not significantly affect participants' degree of trust in the organization, $F(2, 132) = 1.18$, $p = .31$ (REPLICATION: not included). Participants trusted the company to a similar degree in the cash only, large perk, and small perk conditions (*M*s = 4.42, 4.15, and 4.09, *SD*s = 1.22, .94, and 1.11, respectively) (REPLICATION: not included).

In contrast, there was a statistically significant effect of the compensation received by its leader on trust in Somalian Hunger Relief, $F(2, 119) = 5.22$, $p = .007$ (REPLICATION: not included). The charity was trusted significantly less in both the large perk ($M = 4.02$, $SD = 1.36$) (REPLICATION: not included), and small perk conditions ($M = 3.73$, $SD = 1.33$) (REPLICATION: not included), than in the cash only condition ($M = 4.68$, $SD = 1.10$), $t(80) =$

2.32, p = .02, and t(72)= 3.31, p = .001 (REPLICATION: not included), respectively. Somalian Hunger Relief was (dis)trusted to a similar degree in the two perk conditions, t(86) = 1.03, p = .31 (REPLICATION: not included).

Betrayal. No significant effects were observed for the betrayal item. There was no organization type by target compensation interaction, F(2, 258) = .41, p = .66 (REPLICATION: not included), although a marginally significant main effect of compensation did emerge, F(2, 258) = 2.63, p = .07 (REPLICATION: not included). The effect of compensation on feelings of betrayal did not reach significance either for the company, F(2, 136) = .77, p = .47 (REPLICATION: not included), or the charity, F(2, 122) = 2.10, p = .13 (REPLICATION: not included). Although speculative, the compensation paid by an unfamiliar organization with which the participant has never had any prior dealings may be insufficient to elicit feelings of betrayal.

Petition. No significant effects were observed for the petition item. There was no interaction between organizational type and target compensation, F(2, 256) = .43, p = .65 (REPLICATION: not included), nor any significant main effects of organization type, F(1, 256) = .07, p = .79 (REPLICATION: not included), or compensation, F(2, 256) = 1.09, p = .34 (REPLICATION: not included). In addition, no significant effect of how the target candidate was paid on willingness to sign the petition emerged for either the company, F(2, 135) = 1.54, p = .22 (REPLICATION: not included), or the charity, F(2, 121) = .14, p = .87 (REPLICATION: not included).

Social threat. As the control candidate's compensation did not vary by condition, our theoretical hypotheses related only to feelings of threat elicited by the target candidate's

compensation. The expected interaction between type of organization and compensation did not reach significance when it came to feelings of social threat caused by the target candidate, $F(2, 258) = 1.32$, $p = .27$ (REPLICATION: not included). However, further analyses revealed a potentially informative pattern of results. Specifically, whether the Jens Shoes Corporation chose a candidate who requested frivolous perks did not appear to affect whether participants saw the world as a chaotic, unstable, and threatening place, $F(1, 136) = 1.01$, $p = .37$ (REPLICATION: not included). Endorsement of the social threat items was similar in the cash only, large perk, and small perk conditions (*M*s = 2.91, 3.16, and 3.36, *SD*s = 1.63, 1.48, and 1.44, respectively) (REPLICATION: not included).

In contrast, whether Somalian Hunger Relief chose the candidate who requested a perk *did* impact social threat, $F(2, 122) = 5.33$, $p = .006$ (REPLICATION: not included). Contrary to our hypothesis, there was no significant difference in social threat between the cash only ($M = 2.83$, $SD = 1.58$) (REPLICATION: not included) and large perk conditions ($M = 3.22$, $SD = 1.60$) (REPLICATION: not included), $t(83) = 1.11$, $p = .27$ (REPLICATION: not included), although the means were in the expected direction. More consistent with our hypotheses, social threat was significantly greater in the small perk condition ($M = 4.03$, $SD = 1.76$) (REPLICATION: not included) than the cash only condition, $t(74) = 3.11$, $p = .003$ (REPLICATION: not included).

In sum, some noteworthy differences emerged in the reputational consequences of frivolous perks when it came to the leader of a company versus a charity. Participants tolerated a comparatively small perk (i.e., expensive mineral water) in the case of a corporate leader, but balked at a large one (i.e., a chauffeured limousine). In contrast, for the head of a charity, even a

small perk was regarded very negatively: the expensive mineral water elicited perceptions of a charitable organization's leader that were just as negative as a chauffeured limousine. Moreover, granting a top leader a frivolous perk was seen as a trust violation only for the charity. Reading that a charity had agreed to provide its leader with expensive mineral water further elicited feelings of social threat (Koehler & Gershoff, 2003).

References

Diermeier, D. (2011). *Reputation rules: Strategies for building your company's most valuable asset*. McGraw-Hill.

Kasof, J. (1993). Sex bias in the naming of stimulus persons. *Psychological Bulletin, 113,* 140–163.

Koehler, J. J., & Gershoff, A. D. (2003). Betrayal aversion: When agents of protection become agents of harm. *Organizational Behavior and Human Decision Processes 90*, 244–261.

Tannenbaum, D., Uhlmann, E.L., & Diermeier, D. (2011). Moral signals, public outrage, and immaterial harms. *Journal of Experimental Social Psychology, 47,* 1249-1254.

Study Materials

COMPANY + CASH CONDITION

Instructions: Please read the hiring scenario below and then answer the questions.

The Jens Shoes Corporation is deciding between two candidates for President.

Lisa has an MBA from Harvard Business School and eight years of managerial experience at a sneakers company. She was promoted after developing successful partnerships with several shoe companies that cut overhead and administrative costs substantially. As part of her contract, Lisa is requesting a salary of $400,000 a year.

Karen has an MBA from Ross Business School at the University of Michigan and eleven years of managerial experience at an online shoe company. She was promoted after designing a new capital campaign that raised significantly more investments than her predecessor. As part of her proposed contract, Karen is asking for a salary of $400,000.

COMPANY + LARGE PERK CONDITION

Instructions: Please read the hiring scenario below and then answer the questions.

The Jens Shoes Corporation is deciding between two candidates for President.

Lisa has an MBA from Harvard Business School and eight years of managerial experience at a sneakers company. She was promoted after developing successful partnerships with several shoe companies that cut overhead and administrative costs substantially. As part of her contract, Lisa is requesting a salary of $400,000 a year.

Karen has an MBA from Ross Business School at the University of Michigan and eleven years of managerial experience at an online shoe company. She was promoted after designing a new capital campaign that raised significantly more investments than her predecessor. As part of her proposed contract, Karen is asking for a salary of $350,000 plus $50,000 per year for rental of a chauffeur-driven limo on the weekends.

COMPANY + SMALL PERK CONDITION

Instructions: Please read the hiring scenario below and then answer the questions.

The Jens Shoes Corporation is deciding between two candidates for President.

Lisa has an MBA from Harvard Business School and eight years of managerial experience at a sneakers company. She was promoted after developing successful partnerships with several shoe companies that cut overhead and administrative costs substantially. As part of her contract, Lisa is requesting a salary of $400,000 a year.

Karen has an MBA from Ross Business School at the University of Michigan and eleven years of managerial experience at an online shoe company. She was promoted after designing a new capital campaign that raised significantly more investments than her predecessor. As part of her proposed contract, Karen is asking for a salary of $395,000 plus $5,000 per year for luxury water flown from Sweden.

CHARITY + CASH CONDITION

Instructions: Please read the hiring scenario below and then answer the questions.

The Somalia Hunger Relief Charity is deciding between two candidates for President.

Lisa has an MBA from Harvard Business School and eight years of managerial experience at a children's non-profit. She was promoted after developing successful partnerships with several international charity agencies that cut overhead and administrative costs substantially. As part of her contract, Lisa is requesting a salary of $400,000 a year.

Karen has an MBA from Ross Business School at the University of Michigan and eleven years of managerial experience at an advocacy non-profit. She was promoted after designing a new fundraising campaign that raised significantly more donations than her predecessor. As part of her proposed contract, Karen is asking for a salary of $400,000.

CHARITY + LARGE PERK CONDITION

Instructions: Please read the hiring scenario below and then answer the questions.

The Somalia Hunger Relief Charity is deciding between two candidates for President.

Lisa has an MBA from Harvard Business School and eight years of managerial experience at a children's non-profit. She was promoted after developing successful partnerships with several international charity agencies that cut overhead and administrative costs substantially. As part of her contract, Lisa is requesting a salary of $400,000 a year.

Karen has an MBA from Ross Business School at the University of Michigan and eleven years of managerial experience at an advocacy non-profit. She was promoted after designing a new fundraising campaign that raised significantly more donations than her predecessor. As part of her proposed contract, Karen is asking for a salary of $350,000 plus $50,000 per year for rental of a chauffeur-driven limo on the weekends.

CHARITY + SMALL PERK CONDITION

Instructions: Please read the hiring scenario below and then answer the questions.

The Somalia Hunger Relief Charity is deciding between two candidates for President.

Lisa has an MBA from Harvard Business School and eight years of managerial experience at a children's non-profit. She was promoted after developing successful partnerships with several international charity agencies that cut overhead and administrative costs substantially. As part of her contract, Lisa is requesting a salary of $400,000 a year.

Karen has an MBA from Ross Business School at the University of Michigan and eleven years of managerial experience at an advocacy non-profit. She was promoted after designing a new fundraising campaign that raised significantly more donations than her predecessor. As part of her proposed contract, Karen is asking for a salary of $395,000 plus $5,000 per year for luxury water flown from Sweden.

DEPENDENT MEASURES

Please use the scale below to indicate whether the following characteristics are more true of Lisa or Karen.

Definitely Lisa *Definitely Karen*
1 2 3 4 5 6 7

___Who is a more responsible person?

___Who is probably a more morally upstanding human being?

___Who do you predict will make more responsible decisions as leader?

___Who do you predict will act in the best interests of the organization?

___Who is a more selfish person?

[NOTE: This next item is worded differently between the company and charity conditions]
___Who would you invest money with? [IN COMPANY CONDITION]
___Who would you donate money with? [IN CHARITY CONDITION]

___Who would you hire as President?

How much does Lisa's requested compensation tell you about who she *really* is and what she is *really* like?

Nothing A great deal
1 --------- 2 --------- 3 --------- 4 ---------5 --------- 6 ---------7

How much does Karen's requested compensation tell you about who she *really* is and what she is *really* like?

Nothing A great deal
1 --------- 2 --------- 3 --------- 4 ---------5 --------- 6 ---------7

Please rate your agreement with the following statements

If Somalia Hunger Relief Charity [CHARITY CONDITION]/ Jens Shoes Corporation [COMPANY CONDITION] picked Karen as its President …

Please use the following questions to rate the organization:

Bad Good
1 --------- 2 --------- 3 --------- 4 ---------5 --------- 6 ---------7

Unfavorable Favorable
1 --------- 2 --------- 3 --------- 4 ---------5 --------- 6 ---------7

Negative Positive
1 --------- 2 --------- 3 --------- 4 ---------5 --------- 6 ---------7

NOT at all dependable Very Dependable
1 --------- 2 --------- 3 --------- 4 ---------5 --------- 6 ---------7

NOT at all trustworthy Very Trustworthy
1 --------- 2 --------- 3 --------- 4 ---------5 --------- 6 ---------7

NOT at all reliable Very Reliable
1 --------- 2 --------- 3 --------- 4 ---------5 --------- 6 ---------7

Please rate your agreement with the following statements using the scale provided below.

Strongly Disagree Strongly Agree
1 --------- 2 --------- 3 --------- 4 ---------5 --------- 6 ---------7

If Somalia Hunger Relief Charity [CHARITY CONDITION]/ Jens Shoes Corporation [COMPANY CONDITION] picked Karen as its President:

______ I feel betrayed by the organization's choice for President

______ I would sign an online petition to display my support for the organization

Please rate your agreement with the following statements using the scale provided below.

Strongly Disagree Strongly Agree
1 --------- 2 --------- 3 --------- 4 ---------5 --------- 6 ---------7

___ If Lisa was selected as President of my company, I would feel that the world is unfair.

___ If Lisa was selected as President of my company, I would feel that the world is a less orderly place.

___ If Lisa was selected as President of my company, I would feel that the world is a less certain place.

___ If Karen was selected as President of my company, I would feel that the world is unfair.

___ If Karen was selected as President of my company, I would feel that the world is a less

orderly place.

___ If Karen was selected as President of my company, I would feel that the world is a less certain place.

DEMOGRAPHIC MEASURES

What other activities did you engage in during the survey? ___________________
[NOTE: Subjects' responses are displayed as a string variable in the dataset]

Did you read the instructions? ________________________
[NOTE: If subject indicated yes, this string variable reads "I read the instructions." If not it is blank.]

My age is: _________

Politically, I am*:*

Very Liberal
Liberal
Somewhat Liberal
Moderate
Somewhat Conservative
Conservative
Very Conservative
Haven't given it much thought
Completely unsure

[NOTE: These options appear in the datafile as a string variable]

My gender is (please circle one): Male Female

If not the USA, what country are you from? ____________

Without looking back, was the organization a charity or company?

Charity Company

Without looking back, did one of the candidates request a perk?

Yes No

If yes, which candidate requested the perk?

Karen Lisa

Cold Hearted Prosociality Study

(Uhlmann, Tannenbaum, & Diermeier)

Even publicly supported behaviors can send negative signals about an agent's moral character (e.g., "It's a dirty job, but someone has to do it"). Perhaps some praiseworthy acts—such as sacrificing innocents in order to save a greater number of lives— require people who are deficient in generally positive moral traits such as empathy (Uhlmann, Zhu, & Tannenbaum, 2013). This study tested for an *act-person dissociation* where people view one act as more praiseworthy than another, but also more revealing of negative character traits.

Methods

Participants and Design

Seventy-nine participants (REPLICATION: 3016 participants) were recruited using Mechanical Turk and took part in the survey in return for a small cash payment. The study featured a joint evaluation design in which participants read about two targets and evaluated them relative to one another. Pairing of names (Karen and Lisa) with the two targets (medical research assistant and pet store assistant) was counterbalanced between-subjects. Data were not analyzed until after data collection had terminated, no conditions or participants were excluded, and all dependent measures are described below in full. This study was run together in a packet with another study, but this particular study was always presented first.

Materials and Procedure

Scenario. Participants read about two target persons, "Karen" and "Lisa," two names identified by Kasof (1993) as similar in intelligence, age, and other connotations. The *medical*

research assistant was described as working in a center for cancer research. Her job was to expose mice to radiation to induce tumors, and then give them injections of experimental cancer drugs. The *pet store assistant* worked in a store for expensive pets. Her job was to give gerbils a grooming shampoo and then tie bows on them. The pairing of the names Karen and Lisa with the target descriptions was counterbalanced across participants.

Moral actions. Participants were asked “Whose actions make a greater moral contribution to the world?”, “Whose actions benefit society more?”, “Whose job is more morally praiseworthy?”, and “Whose job duties make a greater moral contribution to society?” (1 = *definitely Karen,* 7 = *definitely Lisa*). Items were scored and aggregated so that lower numbers reflected viewing the medical research assistant’s actions as more praiseworthy (α = .85) (REPLICATION: α = .87).

Moral traits. Participants also assessed who was more caring, coldhearted, aggressive, and kind-hearted (1 = *definitely Karen*, 7 = *definitely Lisa*). Items were scored and aggregated so that lower numbers reflected more positive trait attributions regarding the medical research assistant (α = .74) (REPLICATION: α .83).

Animal testing. Participants were also asked if testing cancer drugs on mice is morally wrong (1 = *definitely wrong*, 4 = *not sure*, 7 = *definitely OK*).

Comprehension check. To see if participants were paying careful attention to the scenario, we asked them to identify which of the two women worked in the pet store. However no participants were removed from analyses based on their responses to this item.

Demographics. Finally, participants reported their age, gender, ethnicity, and political orientation. The complete study materials are provided at the end of this report.

Results and Discussion

Responses on all outcome measures were tested against the scale midpoint of 4 (on a scale of 1-7) since participants made comparative judgments of Karen and Lisa. As expected, the medical research assistant's actions were seen as more praiseworthy than those of the pet store assistant (*M* = 2.04, *SD* = 1.27), $t(77) = -13.67$, $p < .001$ (REPLICATION: (*M* = 2.21; *SD* = 1.25), $t(2924) = -77.34$, $p < .001$). However, and in support of an act-person dissociation, the medical research assistant was also perceived as possessing less positive moral traits relative to the pet store assistant (*M* = 4.56, *SD* = .93), $t(78) = 5.40$, $p < .001$ (REPLICATION: *M* = 4.45, *SD* = .98, $t(2934) = 24.89$, $p < .001$).

NOTE: A conceptual replication of this effect that used separate as opposed to joint evaluation was reported in a footnote by Uhlmann, Zhu, & Tannenbaum (2013).

References

Kasof, J. (1993). Sex bias in the naming of stimulus persons. *Psychological Bulletin, 113,* 140-163.

Uhlmann, E.L., Zhu, L., & Tannenbaum, D. (2013). When it takes a bad person to do the right thing. *Cognition, 126*, 326-334.

Study Materials

INSTRUCTIONS: Please read the paragraphs about the individuals below and answer the questions that come after.

Karen works as an assistant in a medical center that does cancer research. The laboratory develops drugs that improve survival rates for people stricken with breast cancer. As part of Karen's job, she places mice in a special cage, and then exposes them to radiation in order to give them tumors. Once the mice develop tumors, it is Karen's job to give them injections of experimental cancer drugs.

Lisa works as an assistant at a store for expensive pets. The store sells pet gerbils to wealthy individuals and families. As part of Lisa's job, she places gerbils in a special bathtub, and then exposes them to a grooming shampoo in order to make sure they look nice for the customers. Once the gerbils are groomed, it is Lisa's job to tie a bow on them.

Please use this scale for the following items:

Definitely Karen Definitely Lisa
1 2 3 4 5 6 7

_____ Whose actions benefit society more?
_____ Whose job duties make a more moral contribution to society?
_____ Whose job is more morally praiseworthy?
_____ Whose actions make a greater moral contribution to the world?

Who is more likely to have the following traits?

Definitely Karen Definitely Lisa
1 2 3 4 5 6 7

_____ Caring
_____ Cold-hearted
_____ Aggressive
_____ Kind-hearted

In my opinion, testing cancer drugs on mice is:
Definitely wrong *not sure* *Definitely OK*
1 2 3 4 5 6 7

My age is: _________

If not the U.S., what is your nationality?

[Note: Responses coded as:]
1 = “CA”
2= “Canada”
3= “India”
4= “canada”
5= “england”
6= “india”
7= “na”
8= “netherlands”

My ethnicity is:

[Note: Coded as:]
1 = Asian Indian
2 = Black/African-American
3 = East Asian (Japan, Korea, Chinese)
4 = Hispanic (Mexican, Cuban, Puerto Rican, Dominican...)
5 = Other
6 = White

Politically I am:

[Note: Variable will need to be recoded for any correlational analyses]
1 = Completely unsure
2 = Conservative
3 = Haven't given it much thought
4 = Liberal
5 = Moderate
6 = Somewhat conservative
7 = Somewhat liberal
8 = Very conservative
9 = Very liberal

Who worked in a pet store?

Lisa Karen

Burn in Hell Study

(Uhlmann & Diermeier)

This study assessed moral evaluations of corporate executives. Both anecdotal and empirical evidence suggests that top corporate executives are a resented group in the United States (Blendon et al., 1997; Caplan, 2001, 2002). Therefore, participants were asked to indicate the percentage of top corporate executives they believed would burn in hell (given hell exists). Burn-in-hell estimates for corporate executives were compared with those from one positively regarded group (social workers) and an array of groups defined by immoral behaviors (e.g., car thieves, drug dealers, vandals).

Methods

Participants and Design

A hundred and fifty-eight students (REPLICATION: 3430 individuals) participated in the study. Participants were recruited from two dining halls at Yale University (45%) and public campus areas at Northwestern University (55%) and paid $2 for their time. Data were analyzed twice, first between the Yale and Northwestern data collections and then again after data collection was complete. No conditions or participants were excluded from the analyses, and all measures are described below in full.

Materials and Procedure

Who will burn in hell? Participants estimated the percentage of individuals from a variety of social categories who would burn in hell (given that hell exists). The categories were: social workers, drug dealers, shoplifters, non-handicapped people who park in the handicapped spot,

top executives at big corporations, people who sell prescription pain killers to addicts, people who kick their dog when they've had a bad day, car thieves, and vandals who spray graffiti on public property.

Arguments for and against capitalism. As an exploratory measure, participants were further asked to provide free responses indicating the best arguments in favor of and against capitalism. The order in which the arguments and burn-in-hell measures appeared was different between the two samples (capitalism arguments were always first at Northwestern and always second at Yale) (REPLICATION: not included).

Demographic measures. Participants were asked to report their religion, religiosity (*1 = not at all religious, 7 = very religious*), political orientation (*1 = very liberal, 7 = very conservative*), age, gender, ethnicity, education level, and the number of economics classes they had taken. Participants were on average politically liberal ($M = 2.79$, $SD = 1.32$) (REPLICATION: $M = 3.28$, $SD = 1.46$; this was statistically lower than 4, the midpoint of the scale, $t(902) = -14.91$, $p < .001$), and 65% (REPLICATION: not included) had taken at least one economics class. The complete study measures are provided at the end of this report.

Results and Discussion

Participants estimated that 42% ($SD = 30\%$) (REPLICATION: 35%, $SD = 32\%$) of top executives at big corporations would burn in hell—a figure significantly lower than drug dealers ($M = 59\%$, $SD = 32\%$) (REPLICATION: $M = 52\%$, $SD = 34.93$), $t(152) = -5.18$, $p < .001$ (REPLICATION: $t(3337) = -24.74$, $p < .001$), people who kick their dogs when they've had a bad day ($M = 59\%$, $SD = 33\%$) (REPLICATION: $M = 60\%$; $SD = 17\%$), $t(152) = -5.83$, $p < .001$ (REPLICATION: $t(3320) = -7.89$, $p < .001$), people who sell prescription pain killers to addicts

(*M* = 55%, *SD* = 31%) (REPLICATION: *M* = 46%, *SD* = 34%), $t(152) = -4.57$, $p < .001$ (REPLICATION: $t(3409) = -19.19$, $p < .001$), car thieves (*M* = 50%, *SD* = 30%) (REPLICATION: *M* = 48%, *SD* = 37%), $t(152) = -2.49$, $p = .014$ (REPLICATION: $t(3289) = -16.82$, $p < .001$), not significantly different from shoplifters (*M* = 39%, *SD* = 29%) (REPLICATION: *M* = 35%, *SD* = 31%), $t(152) = -1.02$, $p = .31$ (REPLICATION: $t(3364) = 1.29$, $p = .20$), and significantly greater than social workers (*M* = 17%, *SD* = 19%) (REPLICATION: *M* = 14%, *SD* = 20%), $t(152) = 9.53$, $p < .001$ (REPLICATION: $t(3298) = 43.04$, $p < .001$), non-handicapped people who park in the handicapped spot (*M* = 32%, *SD* = 30%) (REPLICATION: 28%, *SD* = 32%), $t(151) = 3.96$, $p < .001$ (REPLICATION: $t(3416) = 13.15$, $p < .001$), and vandals (*M* = 34%, *SD* = 29%) (REPLICATION: *M* = 28%, *SD* = 29%), $t(152) = 2.82$, $p = .005$ (REPLICATION: $t(3211) = 13.66$, $p < .001$).

Political conservatives were significantly less likely than liberals to believe that top corporate executives would burn in hell, $r(151) = -.21$, $p = .009$ (REPLICATION: not included). Having taken classes in economics likewise predicted leniency towards executives, $r(150) = -.23$, $p = .005$ (REPLICATION: not included). In contrast, more years of education in general predicted higher burn-in-hell estimates for corporate executives, $r(152) = .25$, $p = .002$ (REPLICATION: not included). None of the other individual differences measures significantly predicted burn-in-hell estimates for executives.

Because there were more liberal than conservative participants in our sample, we also examined burn-in-hell estimates selecting only participants who scored 5 or higher on our 1-7 point political orientation measure (i.e., true conservatives). While more lenient toward corporate executives than liberals were, conservatives did consider them (REPLICATION: *M* = 31%, *SD* =

28%) morally comparable to non-handicapped people who park in the handicapped spot (*M*s = both 29%, *SD*s = 25% and 22%, respectively) (REPLICATION: *M* = 31%, *SD* = 31%). Conservatives believed that the majority of drug dealers (*M* = 74%, *SD* = 29%) (REPLICATION: *M* = 57%, *SD* = 35%), shoplifters (*M* = 51%, *SD* = 28%) (REPLICATION: *M* = 41%, *SD* = 31%), people who sell prescription pain killers to addicts (*M* = 64%, *SD* = 30%) (REPLICATION: *M* = 50%, *SD* = 34%), people who kick their dogs when they've had a bad day (*M* = 54%, *SD* = 36%) (REPLICATION: *M* = 57%, *SD* = 36%), and car thieves (*M* = 63%, *SD* = 29%) (REPLICATION: *M* = 52%, *SD* = 33%) would burn in hell, and that 44% (*SD* = 31%) (REPLICATION: *M* = 35%, *SD* = 31%) of vandals would join them.

References

Blendon, R.J., Benson, J.M., Brodie, M., Morin, R., Altman, D.E., Gitterman, G., Brossard, M., & James, M. (1997). Bridging the gap between the public's and economists' views of the economy. *Journal of Economic Perspectives, 11,* 105-118.

Caplan, B. (2001). What makes people think like economists? Evidence on economic cognition from the 'Survey of Americans and Economists on the Economy'. *Journal of Law and Economics, 43*, 395-426.

Caplan, B. (2002). Systematically biased beliefs about economics: Robust evidence of judgmental anomalies from the 'Survey of Americans and Economists on the Economy'. *Economic Journal, 112*, 433-458.

Study Materials

Assume for a moment that hell exists. What percentage of people in the following categories would go to hell when they die?

Social Worker
% to hell _____

Drug Dealer
% to hell _____

Shoplifter
% to hell _____

Non-handicapped people who park in the handicapped spot
% to hell _____

Top Executives at big corporations
% to hell _____

People who sell prescription painkillers to addicts
% to hell _____

People who kick their dogs when they have a bad day
% to hell _____

Car Thieves
% to hell _____

Vandals who spray graffiti on public property
% to hell _____

Please list what you consider the top argument IN FAVOR of capitalism

1. ____

Please list what you consider the top argument AGAINST capitalism

1. ____

My religion is *(please circle one):*

1 Protestant

If a particular denomination, please indicate here _________________

2 Catholic **5 Islam**

3 Judaism **6 Buddhism**

4 Atheist **7 Agnostic**

8 Other (please indicate) _________________

I consider myself to be:

Not at all Religious						Very Religious
1	2	3	4	5	6	7

Politically, I am *(please circle one):*

1 Very Liberal **5 Somewhat Conservative**

2 Liberal **6 Conservative**

3 Somewhat Liberal **7 Very Conservative**

4 Moderate

My gender is *(please circle one)*: **1 Male** **2 Female**

My age is: __________

What country are you from? ______________

My ethnicity is (please circle one): **1 White** **2 Asian** **3 Latino** **4 Black**
5 Other: ___________________

My educational level is:

1 High school degree or less
2 Some college
3 Currently an undergraduate student
4 College degree
5 Pursuing an MBA
6 Have been awarded an MBA
7 Graduate degree

My occupation is: ________________

My income level is: _______________

Please list the approximate number of economics classes you have taken: ____________

Bigot-Misanthrope Study

(Uhlmann, Tannenbaum, Zhu, & Diermeier)

Acts of everyday racial bigotry may provoke moral outrage in large part because they are perceived as strong signals of poor character (Uhlmann, Zhu, & Diermeier, 2014; see also Pizarro & Tannenbaum, 2011; Tannenbaum, Uhlmann, & Diermeier, 2011; Uhlmann, Pizarro, & Diermeier, in press). In this study, participants evaluated either a CEO who was selectively rude only to Black employees or a CEO who was indiscriminantly hostile and rude to all of his employees. Our prediction was that participants would view the bigot as a worse person than the misanthrope, despite the fact that the misanthrope mistreated a greater number of people. We further expected that the bigoted CEO's behavior, compared to the misanthrope, would be seen as more informative about his moral character. Finally, we predicted that participants would express greater willingness to affiliate with the misanthrope than the bigot, and also that they would expect the misanthrope to act more prosocially than the bigot in future interactions.

Methods

Participants and Design

Forty-six participants (REPLICATION: 3040 participants) were recruited from Amazon's Mechanical Turk and took part in the study in return for a small cash payment. The study featured a simple joint evaluation design in which participants read about two targets and evaluated them relative to one another. Pairing of names (Robert and John) with the two targets (Bigot and Misanthrope) was counterbalanced between-subjects. Data were not analyzed until

after data collection had terminated, no participants were excluded from the analyses, and all conditions and dependent measures are described below in full.

Materials and Procedures

Scenario. Participants were asked to give their impressions of two CEOs, “Robert” and “John,” who worked at similar but different companies. John did not say "hi" or engage in friendly small talk with any of his employees. Robert always said "hi" and engaged in friendly small talk with his White employees, but not his Black employees. John and Robert were selected as names because they were identified by Kasof (1993) as similar in intelligence, age, and other connotations.

After reading the scenario, participants responded to a series of relative evaluation items on seven-point scales ranging from 1 (*Definitely John*) to 7 (*Definitely Robert*)..

Person judgments. To assess character-based judgments, participants were asked whether John or Robert was the more immoral and blameworthy person ($\alpha = .91$) (REPLICATION: $\alpha = .75$). Responses were coded so that lower numbers reflected relatively greater condemnation of the bigot’s moral character.

Informational value. To assess how informative they found each behavior, participants were asked to determine which person's behavior “tells you more about their moral character” and “tells you more about their personality” ($\alpha = .68$; items adapted from Tannenbaum et al., 2011) (REPLICATION: $\alpha = .43$). Responses were coded so that lower numbers indicated that participants viewed the bigot’s behavior as more informative than the misanthrope’s.

Affiliation. Participants were asked who they would rather have as a close personal friend, date their daughter, have as a co-worker, and whose unlaundered sweater they would

rather wear ($\alpha = .60$) (REPLICATION: not included). Responses were coded so that lower numbers reflected greater willingness to affiliate with the bigot.

Anticipated future behavior. Participants responded to a single item about who they thought was more likely to behave immorally in the future. Responses were coded such that lower numbers reflected more favorable expectations about the bigot's future behaviors (REPLICATION: not included).

Free responses. Participants were told "If you had a preference for either John or Robert, please briefly tell us why" and were provided with space to respond in their own words.

Comprehension check. We asked participants to identify which CEO was selectively rude to his employees, with the options *Robert, John,* and *Neither* provided. However no participants were removed from the analyses based on their answer.

Demographics. Finally, participants reported their age, gender, ethnicity, nationality, and political orientation. The complete study materials are provided at the end of this report.

Results and Discussion

Because all items involved providing relative evaluations of the two targets, average responses to each measure were compared against the scale midpoint of 4 (scales ranged from 1 to 7). Participants judged the bigoted CEO more negatively than the misanthropic CEO (M = 2.66, SD = 1.49), $t(45) = -6.07$, $p < .001$ (REPLICATION: M = 2.38; SD = 1.36, $t(2956) = -64.57$, $p < .001$), and the bigot's behavior was also perceived as more informative about his moral character (M = 3.04, SD = 1.56), $t(45) = 4.17$, $p < .001$ (REPLICATION: M = 2.65; SD = 1.41, $t(2962) = 51.93$, $p < .001$). Participants also expressed greater willingness to affiliate with the misanthrope than the bigot (M = 4.68, SD = 1.25), $t(44) = 3.64$, $p = .001$ (REPLICATION:

not included), but (contrary to our expectations) did not anticipate more ethical future behavior from the misanthrope (M = 3.96, SD = 2.03), $t < 1$ (REPLICATION: not included).

NOTE: An unpublished conceptual replication of this effect that used separate as opposed to joint evaluation of targets is described in an online posting here:

Zhu, L., Uhlmann, E.L., & Diermeier, D. (2014). *Moral evaluations of bigots and misanthropes*. Study report available at: https://osf.io/a4uxn/

References

Kasof, J. (1993). Sex bias in the naming of stimulus persons. *Psychological Bulletin, 113,* 140-163.

Pizarro, D. A., & Tannenbaum, D. (2011). Bringing character back: How the motivation to evaluate character influences judgments of moral blame. In P. Shaver & M. Mikulincer (Eds.), *The social psychology of morality: Exploring the causes of good and evil.* New York: APA books.

Tannenbaum, D., Uhlmann, E.L., & Diermeier, D. (2011). Moral signals, public outrage, and immaterial harms. *Journal of Experimental Social Psychology*, *47,* 1249-1254.

Uhlmann, E.L., Pizarro, D., & Diermeier, D. (in press). A person-centered approach to moral judgment. *Perspectives on Psychological Science.*

Uhlmann, E.L., Zhu, L., & Diermeier, D. (2014). When actions speak volumes: The role of inferences about moral character in outrage over racial bigotry. *European Journal of Social Psychology, 44*, 23-29

Study Materials

NOTE: Pairing of names (Robert and John) with the bigoted vs. misanthropic targets was counterbalanced between-subjects.

Instructions: We would like to get your impressions about two CEOs, Robert and John, who work at similar but different companies.

John is a CEO at Company X. John does not say "hi" or engage in friendly small talk with any of his employees. When an employee says "hi", John never responds.

Robert is a CEO at Company Y. Robert always says "hi" and engages in friendly small talk with his White employees. But when an African American employee says "hi," Robert never responds.

(At both companies, about 80% of co-workers are White, and about 20% are African American)

Who is a more immoral person?
Definitely John Definitely Robert
1 2 3 4 5 6 7

Who is more morally blameworthy as a person?
Definitely John Definitely Robert
1 2 3 4 5 6 7

Which person's action tells you more about their moral character?
Definitely John Definitely Robert
1 2 3 4 5 6 7

Whose behavior towards their co-worker tells you more about their personality?
Definitely John Definitely Robert
1 2 3 4 5 6 7

Who would you rather have as a close personal friend?
Definitely John Definitely Robert
1 2 3 4 5 6 7

Who would you rather have date your daughter?
Definitely John Definitely Robert
1 2 3 4 5 6 7

Who would you rather have as a co-worker?
Definitely John Definitely Robert
1 2 3 4 5 6 7

Who is more likely to behave immorally in the future?

Definitely John Definitely Robert

1 2 3 4 5 6 7

Whose unlaundered sweater would you rather wear?

Definitely John Definitely Robert

1 2 3 4 5 6 7

If you had a preference for either John or Robert, please briefly tell us why: _________________
____

Which one of the CEOs was rude to some of his employees, but nice to others?

Robert
John
Neither

How old are you? _________

If not the USA, please indicate your nationality: _______________

What is your gender?

Male
Female

Your ethnicity is: _____________________
[NOTE: Responses are listed in the data file as a string variable]

When it comes to politics, I am generally: ____________________
[NOTE: Responses are listed in the data file as a string variable]

Bad Tipper Study

(Uhlmann, Tannenbaum, & Diermeier)

Our previous work finds that some acts are seen as strong signals of poor moral character even when the act itself is viewed as relatively benign (Tannenbaum, Uhlmann, & Diermeier, 2011; Uhlmann, Pizarro, & Diermeier, in press). Minor acts of everyday incivility seem like a context in which individuals can communicate negative information about themselves without causing much material harm to others. We therefore expected that leaving a restaurant tip entirely in pennies would be seen as highly informative of poor character, even though the act would not be viewed as morally blameworthy in-and-of itself.

Methods

Participants and Design

We recruited a sample of 79 participants (REPLICATION: 3706 participants) from Mechanical Turk, who each completed the survey in return for a small cash payment. Data were not analyzed until after data collection had terminated, no participants or conditions were excluded for any reason, and all dependent measures are described below in full. The study featured two between-subjects conditions. We administered this study as part of a packet of several studies; participants always completed this particular study after first responding to another study.

Materials and Procedures

Scenario. Participants read about a restaurant patron named Jack who was satisfied with his meals and service. Given the bill, the expected tip would be $15. In the *bills condition,* Jack

left $14 in bills, thus paying less than what was appropriate. In the *pennies condition*, Jack paid the full gratuity of $15 by leaving a bag of pennies.

Person judgments. To assess character-based judgments, participants were asked whether Jack was a disrespectful person, had a good moral conscience, was a good person, and was the type of person they would want as a friend (*1 = Not at all, 7 = Definitely*). For the analyses, these items were coded such that higher scores indicated more negative person judgments (α = .84) (REPLICATION: α = .86).

Act judgment. As a measure of their act-based evaluations, participants were asked how blameworthy Jack's behavior was (*1 = Not at all blameworthy, 7 = Completely blameworthy*).

Informational value. To assess how informative they viewed Jack's behavior, participants were asked "Do you think this behavior tells you a lot or a little about Jack's personality?" (*1= Says nothing about Jack, 7 = Says a lot about Jack*; this item was adapted from Tannenbaum et al., 2011).

Demographics. Finally, participants reported their age, gender, ethnicity, nationality, and political orientation. All study materials are provided below this report.

Results and Discussion

Jack was viewed as a worse person when he left a $15 tip in pennies than when he left a $14 tip in bills (*M*s = 4.41 and 3.57, *SD*s = 1.27 and 1.35), $t(75) = -2.79$, $p = .007$ (REPLICATION: *M* s= 4.13 and 3.33, *SD*s = 1.26 and 1.29, $t(3645) = -18.96$, $p < .001$). Tipping in pennies was also more informative about his character than when Jack tipped with bills (*M*s = 5.41 and 3.45, *SD*s = 1.60 and 1.81 (REPLICATION: *M*s = 4.65 and 3.42, *SD*s = 1.76 and 1.77), $t(76) = -4.98$, $p < .001$ (REPLICATION: $t(3680) = -20.98$, $p < .001$). Contrary to our act-person

dissociation hypothesis, the act of paying in pennies was also rated as more morally blameworthy than paying in bills (*M*s = 4.56 and 3.52, *SD*s = 1.94 and 1.80) (REPLICATION: *M*s = 3.94 and 2.92, *SD*s = 1.85 and 1.81), $t(76) = -2.44$, $p = .017$ (REPLICATION: $t(3676) = -16.81$, $p < .001$). Also, act and person judgments were highly correlated, $r(76) = .75$, $p < .001$ (REPLICATION: $r(3647) = .70$, $p < .001$).

As expected, a person who paid the full tip with a bag of pennies was judged more negatively than a person who tipped less well but in bills. Tipping in pennies was also viewed as relatively more informative about moral character. However, a dissociation between and act and person judgments (Tannenbaum et al., 2011; Uhlmann et al., in press) did not emerge, as the act of tipping in pennies was also seen as more blameworthy than tipping in bills. Although speculative, tipping in pennies might be seen as causing harm because it inconveniences and upsets the waiter or waitress, making the act itself morally wrong. Future research will examine this possibility, and explore moral judgments of everyday incivility in other contexts.

References

Tannenbaum, D., Uhlmann, E.L., & Diermeier, D. (2011). Moral signals, public outrage, and immaterial harms. *Journal of Experimental Social Psychology*, *47,* 1249-1254.

Uhlmann, E.L., Pizarro, D., & Diermeier, D. (in press). A person-centered approach to moral judgment. *Perspectives on Psychological Science.*

Study Materials

BILLS CONDITION:

Instructions: We would now like you to read about a person named Jack.

Jack is eating dinner at a restaurant. The expected gratuity for his bill would be approximately $15. Satisfied with his meal and service, Jack places a few bills on the table (totaling to $14) before he leaves.

PENNIES CONDITION:

Instructions: We would now like you to read about a person named Jack.

Jack is eating dinner at a restaurant. The expected gratuity for his bill would be approximately $15. Satisfied with his meal and service, Jack places a large bag of pennies on the table (totaling to $15) before he leaves.

DEPENDENT MEASURES:

Do you think that Jack is probably a disrespectful person?

Not at all Definitely
1 2 3 4 5 6 7

Do you think that Jack probably has a good moral conscience?

Not at all Definitely
1 2 3 4 5 6 7

Is Jack the type of person that you would want as a close friend?

Not at all Definitely
1 2 3 4 5 6 7

Would you say that in general, Jack is a good person?

Not at all Definitely
1 2 3 4 5 6 7

Strickly speaking, how blameworthy was Jack's behavior?

Not at all blameworthy Completely blameworthy
1 2 3 4 5 6 7

Do you think this behavior tells you a lot or a little about Jack's personality?

Says nothing about Jack					Says a lot about Jack	
1	2	3	4	5	6	7

DEMOGRAPHICS:

My age is: _________

If not the U.S., what is your nationality?

[Note: Responses coded as:]
1 = Canada
2 = Croatia
3 = Germany
4 = Great Britain
5 = India
6 = Philippines
7 = Romania

My ethnicity is:

[Note: Responses coded as:]
1 = American Indian, Alaska native
2 = Asian Indian
3 = Black/African-American
4 = East Asian (Japan, Korea, Chinese)
5 = Hispanic (Mexican, Cuban, Puerto Rican, Dominican...)
6 = Other
7 = Pacific islander
8 = Southeast Asian (Vietnam, Cambodia, Malaysia, Indonesia...)
9 = White

Politically I am:

[Note: This variable will need to be recoded for any correlational analyses given the unusual number scheme]
1 = Completely unsure
2 = Conservative
3 = Haven't given it much thought
4 = Liberal
5 = Moderate
6 = Somewhat conservative
7 = Somewhat liberal
8 = Very conservative
9 = Very liberal

Belief-Act Inconsistency Study

(Uhlmann, Tannenbaum & Diermeier)

Do people disapprove of moral hypocrisy? The answer seems to be a straightforward Yes. Many instances of hypocrisy, however, are conflated with behavior that we find unacceptable even when hypocrisy is absent. Take the example of a politician who prosecutes criminals only to engage in corruption himself, or a religious leader who chastises sexually impropriety from the church pulpit and is later discovered having sex with a prostitute. In such cases our moral reactions may reflect our genuine distaste for hypocrisy, or it may simply reflect distaste for corruption and the solicitation of prostitutes. This study examined whether people have a direct distaste for hypocrisy even when they find the underlying behavior perfectly acceptable.

Methods

Participants and Design

One hundred ninety two Northwestern students (REPLICATION: 3708 participants) took part in the study, and each participant was randomly assigned to one of three conditions (animal rights advocate, doctors without borders advocate, big game hunting advocate). Participants were recruited in a public area on the university's campus and were paid $2 for their time. Data were analyzed after 95 subjects had been collected and after 192 subjects had been collected. No conditions or participants were excluded from the analyses, and all measures are described below in full. An unrelated study examining activation of concepts related to lawsuits after reading about different kinds of car accidents was administered after participants completed the current

study. In the dataset, variables associated with this unrelated study have names with "law" in them.[1]

Material and Procedures

Scenario. In the *animal rights condition*, participants read about Bob Hill, who had worked for 20 years as an animal rights activist and president of the non-profit organization Furry Friends Forever (FFF). FFF's mission was to advocate for the ethical treatment of domestic and wild animals through public education, cruelty investigations, research, animal rescue, legislation, special events, celebrity involvement, and protest campaigns. In the *doctors-without-borders condition*, Bob Hill was instead an advocate for and president of Doctors Without Borders (DWB), which provides medical aid to people in nearly 60 countries. In the *big game hunting condition,* Bob Hill was a hunting advocate and president of the American Big Game Hunters Association (ABGA). In all conditions, the Associated Press news service reported that Hill had recently participated in a wild game hunting safari in South Africa. Included along with the scenario was a picture showing Hill with a slain antelope and Winchester Magnum hunting rifle.

Hitler-Mother Teresa ratings. We included an item intended to mimic the "slider scales" sometimes used in online surveys. This scale featured a horizontal line anchored by a picture of Adolf Hitler on the left and Mother Teresa on the right. Participants were instructed to indicate how morally good or bad a person they found Bob to be by marking an X on the line. Although this seemed straightforward to us, participants may not have fully understood the measure and nearly half (44.8%) left no "X" (REPLICATION: not included). Due to the large amount of missing data, results for this item were not analyzed.

Moral blame. Participants were asked how morally blameworthy or morally praiseworthy they found Bob as a person on a Likert scale ranging from -5 (*Extremely Blameworthy*) to +5 (*Extremely Praiseworthy*).

Warmth. Another item asked participants how warm or cold they felt towards Bob (*-5 = Incredibly cold, +5 = Incredibly warm*).

Trust. Trust in Bob was assessed using responses to an item ranging from -5 (*Incredibly untrustworthy*) to +5 (*Incredibly trustworthy*).

Hypocrisy. A final dependent measure asked whether Bob was a hypocrite (0 = *Not at all, 10 = Definitely).*

Hunting attitudes. To assess individual differences in attitudes towards hunting, participants were asked "How do you feel about the activity of hunting wild (non-endangered) animals?" (*-5 = Very Wrong, +5 = Perfectly Okay*).

Comprehension checks. A free response item asked participants to describe the type of organization Bob belonged to. Participants also filled out two comprehension checks for the unrelated study. No participants were removed from the analyses based on their responses to any of the comprehension checks (REPLICATION: not included).

Protected values. We also included an exploratory measure of whether participants viewed animal rights as a protected value. They were asked to choose whether protecting animals should only be done if it leads to large benefits, should be done no matter how small the benefits, or should not be done if it saves enough money. Selecting the second option indicated a protected value (REPLICATION: not included).

Demographic measures. Finally, participants reported their religion, degree of religiosity (*0= not at all religious, 10 = very religious*) , political orientation (*1 = very liberal, 7 = very conservative*), gender, age, ethnicity, number of years in the U.S., nationality if not from the U.S., education level of their most educated parent, parents' occupations, and family income. The complete study measures are provided at the end of this report.

Results and Discussion

Consistent with a direct aversion to moral hypocrisy, we found a significant effect of experimental condition for moral blame $F(2, 186) = 42.53$, $p < .001$ (REPLICATION: $F(2, 3109) = 423.10$, $p < .001$), warmth, $F(2, 189) = 35.44$, $p < .001$ (REPLICATION: $F(2, 3107) = 259.94$, $p < .001$), trust, $F(2, 189) = 48.22$, $p < .001$ (REPLICATION: $F(2, 3090) = 221.61$, $p < .001$), and perceived hypocrisy $F(2, 189) = 48.67$, $p < .001$ (REPLICATION: $F(2, 3078) = 613.56$, $p < .001$). Individual differences in attitudes towards hunting did not differ by condition, $F(2, 189) = .68$, $p = .51$ (REPLICATION: did differ, $F(2, 3110) = 8.17$, $p < .001$).

Participants viewed the animal rights activist who was caught hunting, compared to the big game hunter who was caught hunting, as more blameworthy (*M*s = -1.58 and -.92, *SD*s = 1.81 and 1.72) (REPLICATION: *M*s = -2.57 and -1.77, *SD*s = 2.44 and 2.38), $t(124) = -2.11$, $p = .037$ (REPLICATION: $t(2065) = -7.57$, $p <.001$ .037), less trustworthy (*M*s = -2.23 and -.05, *SD*s = 1.97 and 1.73) (REPLICATION: *M*s = -2.87 and -.67, *SD*s = 2.40 and 2.20), $t(126) = -6.65$, $p < .001$ (REPLICATION: $t(2061) = -21.73$, $p < .001$), and more hypocritical (*M*s = 6.94 and 2.60, *SD*s = 2.81 and 2.35) (REPLICATION: *M*s = 8.75 and 4.33, *SD*s = 2.80 and 2.94), $t(126) = 9.45$, $p < .001$ (REPLICATION: $t(2044) = 34.82$, $p < .001$). However, both targets were viewed as low in warmth, and we did not find a reliable difference between the two conditions (*M*s = -1.52 and

-1.21, *SD*s = 1.77 and 1.76 (REPLICATION: *M*s = -2.33 and -1.97, *SD*s = 2.32 and 2.34), $t(126) = -1.02$, $p = .31$ (REPLICATION: significant difference, $t(2063) = -3.58$, $p < .001$).

Compared to the hunter who was an advocate for an unrelated charity (doctors without borders), the animal rights activist was seen as more blameworthy (*M*s = -1.58 and 1.41, *SD*s = 1.82 and 2.20) (REPLICATION: *M*s = -2.57 and .58, *SD*s = 2.44 and 2.85), $t(126) = -8.42$, $p < .001$ (REPLICATION: $t(2071) = -27.01$, $p < .001$), less warm (*M*s = -1.52 and 1.06, *SD*s = 1.77 and 2.14 (REPLICATION: *M*s = -2.33 and -.08, *SD*s = 2.32 and 2.58)), $t(127) = -7.49$, $p < .001$ (REPLICATION: $t(2068) = -20.89$, $p < .001$), less trustworthy (*M*s = -2.23 and 1.19, *SD*s = 1.97 and 2.27 (REPLICATION: *M*s = -2.87 and -1.88, *SD*s = 2.40 and 2.52)), $t(127) = -9.14$, $p < .001$ (REPLICATION: $t(2055) = -9.14$, $p < .001$), and more hypocritical (*M*s = 5.94 and 3.36, *SD*s = 2.81 and 2.72) (REPLICATION: *M*s = 8.75 and 5.35, *SD*s = 2.80 and 3.21), $t(127) = 7.35$, $p < .001$ (REPLICATION: $t(2058) = 25.59$, $p < .001$).

These results held selecting only those participants who expressed moral approval of hunting (i.e., who responded above the scale midpoint of zero on our hunting attitudes measure). A significant effect of condition emerged for blame $F(2, 67) = 25.16$, $p < .001$ (REPLICATION: $F(2, 1359) = 284.49$, $p < .001$), warmth, $F(2, 69) = 33.95$, $p < .001$ (REPLICATION: $F(2, 1355) = 166.37$, $p < .001$), trust, $F(2, 69) = 32.22$, $p < .001$ (REPLICATION: $F(2, 1354) = 108.28$, $p < .001$), and hypocrisy $F(2, 69) = 22.39$, $p < .001$ (REPLICATION: $F(2, 1344) = 345.70$, $p < .001$).

Compared to the big game hunter, the animal rights activist who was caught big game hunting was perceived as more blameworthy (*M*s = -.68 and .72, *SD*s = 1.82 and 1.02) (REPLICATION: *M*s = -1.67 and -.06, *SD*s = 2.58 and 2.02), $t(41) = -2.95$, $p = .005$

(REPLICATION: $t(864) = -10.09$, $p < .001$), less warm (*M*s = -1.76 and .45, *SD*s = 1.45 and 1.10) (REPLICATION: *M*s = -1.32 and -.30, *SD*s = 2.43 and 2.10), $t(43) = -3.09$, $p = .004$ (REPLICATION: $t(860) = -6.58$, $p < .001$), less trustworthy (*M*s = -1.52 and 1.05, *SD*s = 1.83 and 1.43 (REPLICATION: *M*s = -2.01 and .41, *SD*s = 2.67 and 1.81)), $t(43) = -5.15$, $p < .001$ (REPLICATION: $t(861) = -15.45$, $p < .001$), and more hypocritical (*M*s = 5.92 and 1.70, *SD*s = 3.04 and 2.08) (REPLICATION: *M*s = 7.79 and 3.43, *SD*s = 3.16 and 2.47), $t(43) = 5.29$, $p < .001$ (REPLICATION: $t(855) = 22.26$, $p < .001$).

Compared to the doctors without borders advocate, the animal rights activist was also seen as more blameworthy (*M*s = -.68 and 2.48, *SD*s= 1.82 and 1.72) (REPLICATION: *M*s = -1.67 and 1.88, *SD*s = 2.58 and 2.24), $t(50) = -6.45$, $p < .001$ (REPLICATION: $t(955) = -22.73$, $p < .001$), less warm (*M*s = -.76 and 2.37, *SD*s = 1.45 and 1.50) (REPLICATION: *M*s = -1.32 and 1.27, *SD*s = 2.43 and 2.09), $t(50) = -7.64$, $p < .001$ (REPLICATION: $t(952) = -17.71$, $p < .001$), less trustworthy (*M*s = -1.52 and 2.19, *SD*s = 1.83 and 1.73) (REPLICATION: *M*s = -2.01 and -1.43, *SD*s = 2.66 and 2.82), $t(50) = -7.50$, $p < .001$ (REPLICATION: $t(952) = -3.03$, $p = .001$), and more hypocritical (*M*s = 5.92 and 1.81, *SD*s = 3.04 and 2.24) (REPLICATION: *M*s = 7.89 and 3.69, *SD*s = 3.16 and 2.67), $t(50) = 5.58$, $p < .001$ (REPLICATION: $t(947) = 21.63$, $p < .001$).

In sum, an animal rights activist who was caught hunting was seen as an untrustworthy and bad person, even by participants who believed that hunting was morally acceptable. This suggests that an inconsistency between a person's moral beliefs and behaviors may be sufficient to elicit moral condemnation, even when the behavior is not actually seen as immoral in-and-of itself. People, it appears, have a direct aversion to moral hypocrisy.

Footnote

[1] In the unrelated study, participants were randomly assigned to read either about an accident caused by a reckless driver, an accident caused by a negligent company, or a control condition in which no accident occurred (see the study materials below this report). They then filled out thirteen word completions designed to measure the automatic accessibility of words related to lawsuits. Coding of the word stem completion measure was discontinued after the first 142 participants due to its poor psychometric properties.

Original Study Materials

ANIMAL RIGHTS ACTIVIST CONDITION

Bob Hill has worked for 20 years as an animal rights activist and president of the non-profit organization Furry Friends Forever (FFF), which advocates for the ethical treatment of domestic and wild animals. FFF works through public education, cruelty investigations, research, animal rescue, legislation, special events, celebrity involvement, and protest campaigns.

Recently, the Associated Press news service reported that Hill had participated in a wild game hunting safari in South Africa. The report indicated that this is the fourth big game hunting safari that Hill has done in the last five years. Below is a picture that accompanied the press release, showing Hill with a Kudu antelope that he shot down with a .338 Winchester Magnum hunting rifle.

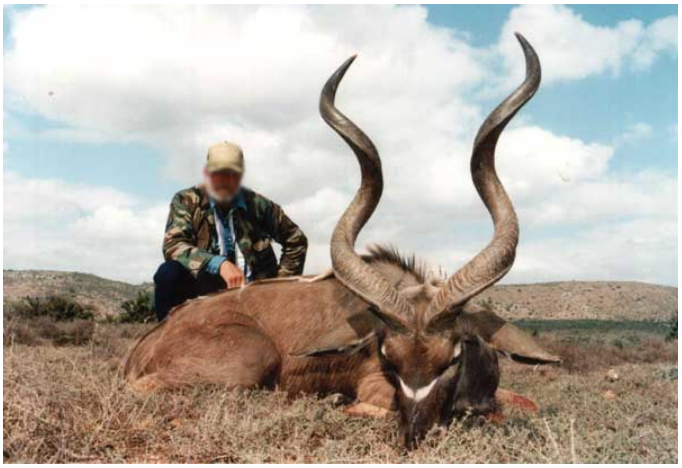

BIG GAME HUNTERS ASSOCIATION CONDITION

Bob Hill has worked for 20 years as an avid hunter and president of the American Big Game Hunters Association (ABGA), which advocates for big game trophy hunting throughout North America and the world. ABGA serves the hunting community through the sharing of experiences, knowledge and technology, promoting the education of youth in securing the future of the hunting tradition, and extending the goodwill of members through community outreach.

Recently, the Associated Press news service reported that Hill had participated in a wild game hunting safari in South Africa. The report indicated that this is the fourth big game hunting safari that Hill has done in the last five years. Below is a picture that accompanied the press release, showing Hill with a Kudu antelope that he shot down with a .338 Winchester Magnum hunting rifle.

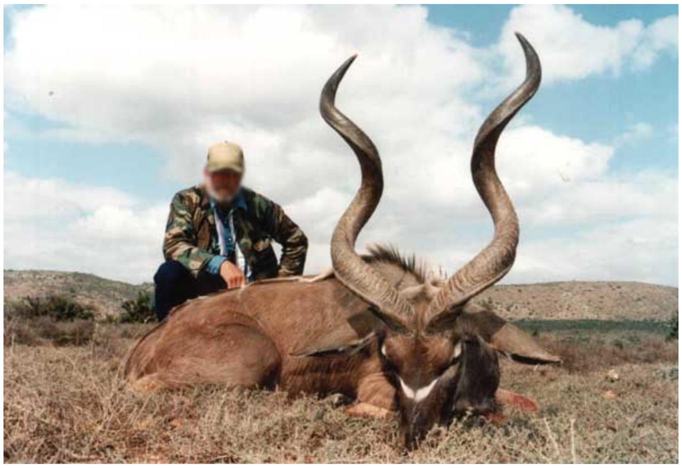

DOCTORS WITHOUT BORDERS CONDITION

Bob Hill has worked for 20 years as a human right activist and president of doctors without borders (DWB), which provides medical aid in nearly 60 countries to people whose survival is threatened by violence, neglect, or catastrophe, primarily due to armed conflict, epidemics, malnutrition, exclusion from health care, or natural disasters. DWB provides independent, impartial assistance to those most in need. DWB is committed to bringing quality medical care to people caught in crisis regardless of race, religion, or political affiliation.

Recently, the Associated Press news service reported that Hill had participated in a wild game hunting safari in South Africa. The report indicated that this is the fourth big game hunting safari that Hill has done in the last five years. Below is a picture that accompanied the press release, showing Hill with a Kudu antelope that he shot down with a .338 Winchester Magnum hunting rifle.

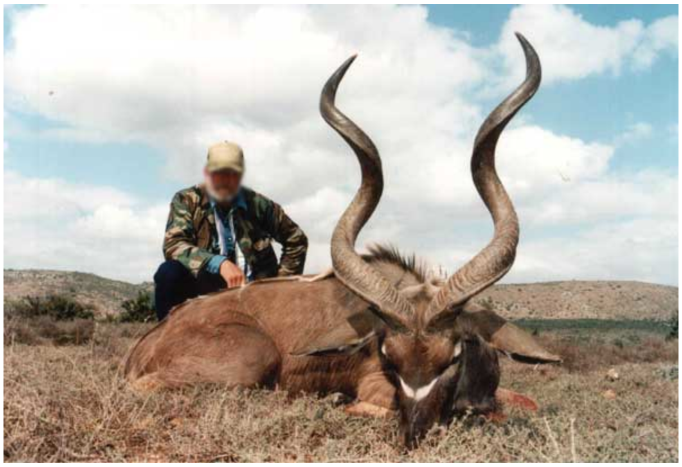

DEPENDENT MEASURES

(1) Please indicate how morally good or bad a person you find Bob to be. To do so, please indicate where you feel Bob falls on the axis below: (*place an X on the line at the point that best represents your answer*)

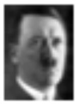

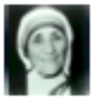

Adolf Hitler *Mother Teresa*

(2) How morally blameworthy or morally praiseworthy do you find Bob as a person?

–5	–4	–3	–2	–1	0	1	2	3	4	5

Extremely Blameworthy *Extremely Praiseworthy*

(3) How much warmth or coldness do you feel personally towards Bob?

–5	–4	–3	–2	–1	0	1	2	3	4	5

Incredibly cold *Incredibly warm*

(4) How trustworthy do you personally find Bob to be?

–5	–4	–3	–2	–1	0	1	2	3	4	5

Incredibly untrustworthy *Incredibly trustworthy*

(5) Do you find Bob to be a hypocrite?

0	1	2	3	4	5	6	7	8	9	10

Not at all *Definitely*

(6) How do you feel about the activity of hunting wild (non-endangered) animals?

–5	–4	–3	–2	–1	0	1	2	3	4	5

Very Wrong *Perfectly Okay*

LITIGIOUSNESS STUDY SCENARIOS

FRIVOLOUS LAWSUIT CONDITION

Instruction: Please read the paragraph below. Later you will be tested on your memory for it.

Tom Patton was recently driving at double the speed limit on the highway, steering his car with his feet and shooting up heroin. On a sharp bend, he failed to turn in time and crashed his car into the highway railing. The railing, manufactured by Highland Road Company, gave way and his car fell down a steep hill. Tom was left with severe neck and back pain and is now unable to keep his job.

LEGITIMATE LAWSUIT CONDITION

Instruction: Please read the paragraph below. Later you will be tested on your memory for it.

Tom Patton was recently driving his car on the highway at the speed limit. He was unable to turn in time on a sharp bend where there are frequent accidents and crashed his car into the highway railing. The railing, manufactured by Highland Road Company, gave way and his car fell down a steep hill. Tom was left with severe neck and back pain and is now unable to keep his job.

NEUTRAL CONDITION

Instruction: Please read the paragraph below. Later you will be tested on your memory for it.

Tom Patton was recently driving his car on the highway at the speed limit. He turned on a sharp bend. The railing on the highway at the sharp bend was manufactured by Highland Road Company.

WORD STEM ACTIVATION DV FOR LITIGIOUSNESS STUDY

Instruction: Below are words that have one or more letters missing. Please add letters to form a complete word.

TRI__ __

___ AW

___AD

___UDGE

___ ITNESS

ANG__ __

S__E

__LEA

R__ __ ING

__ AIL

__IGHT

B__ __ D

__ASE

<u>Without looking back to your previous responses</u>, we would like to ask you some questions about the scenarios you just completed.

In the first scenario you read, please describe the type of organization that Bob belonged to:

____

In the second scenario you read, did Tom crash his car? (*please circle one*)

Yes No

In the second scenario you read, was Tom shooting up heroin while he was driving? (*please circle one*)

Yes No

How do you feel about protecting wild animals (*please check one*)

______ People should only undertake this action if it leads to some benefits that are great enough.

______ People should do this no matter how small the benefits.

______ Not undertaking the action is acceptable if it saves people enough money.

My religion is *(please circle one):*

1 Protestant (*if a particular denomination, please indicate:* ___________)
2 Catholic
3 Judaism
4 Atheist
5 Islam
6 Buddhism
7 Agnostic
8 Other (*please indicate* _________________)

I consider myself to be:

0	1	2	3	4	5	6	7	8	9	10
Not at all religious										*Very religious*

Politically, I am *(please circle one):*

1 Very Liberal
2 Liberal
3 Somewhat Liberal
4 Moderate
5 Somewhat Conservative
6 Conservative
7 Very Conservative

My gender is (*please circle one*): 1 Male 2 Female My age is: _________

How many years have you lived in this country? __________

If you are from a foreign country, please list the country: _____________

My ethnicity is (*please circle one*): 1 White 2 Asian 3 Latino 4 Black
5 Other: _______________

The educational level of my *most highly educated parent* is:

1 High school degree or less 3 College degree
2 Some college 4 Graduate degree

My parents' yearly income level is: _____________

My *parents'* occupations are:________________________________

SUPPLEMENT 3: REPLICATION MATERIALS

This packet includes the following materials:

1. Presumption of guilt study. 4 between-subjects conditions. 1 page long.
2. Moral inversion study. 4 between-subjects conditions. 1 page long.
3. Higher standard study. 6 between-subjects conditions. 1 page long.
4. Belief-act inconsistency study. 3 between-subjects conditions. 1 page long.
5. Moral cliff study. Each subject does both conditions, with scenario order counterbalanced between-subjects. 2 pages long in total.
6. Cold-hearted prosociality study. 2 between subjects conditions. 1 page long.
7. Bad tipper study. 2 between subjects conditions. 1 page long.
8. Bigot misanthrope study. 2 between subjects conditions. 1 page long.
9. Intuitive economics study. 2 between subjects conditions. 4 pages long.
10. Burn in hell study. 1 page study with only 1 condition.
11. Demographics form (same for all studies), 1 page long.

NOTE: This is the "presumption of guilt study", condition *1* of 4. The study uses a between-subjects design with random assignment to one of the four conditions.

Chicago, Ill., December 2, 2013 – The Locks Corporation, based in Rockford, Illinois, today was accused that several of their food products contain a substance known as Gloactimate, which may be harmful to people's health. Gloactimate is an additive in processed foods and is used to increase the shelf life of foods. A recent series of studies found that Gloactimate raises "bad" cholesterol, lowers "good" cholesterol, and increases risk for heart disease.

Corporate Response:

The Locks Corporation announced that it is confident in its adherence to government standards regarding Gloactimate.

Now, please use the following questions to rate the Locks Corporation: *(Circle only one number for each rating)*:

Bad **Good**
1 --------- 2 --------- 3 --------- 4 ---------5 --------- 6 ---------7 --------- 8 --------- 9

Unethical **Ethical**
1 --------- 2 --------- 3 --------- 4 ---------5 --------- 6 ---------7 --------- 8 --------- 9

Immoral **Moral**
1 --------- 2 --------- 3 --------- 4 ---------5 --------- 6 ---------7 --------- 8 --------- 9

Irresponsible **Responsible**
1 --------- 2 --------- 3 --------- 4 ---------5 --------- 6 ---------7 --------- 8 --------- 9

Deceitful **Honest**
1 --------- 2 --------- 3 --------- 4 ---------5 --------- 6 ---------7 --------- 8 --------- 9

Guilty **Innocent**
1 --------- 2 --------- 3 --------- 4 ---------5 --------- 6 ---------7 --------- 8 --------- 9

NOTE: This is the "presumption of guilt study", condition *2* of 4. The study uses a between-subjects design with random assignment to one of the four conditions.

Chicago, Ill., December 2, 2013 – The Locks Corporation, based in Rockford, Illinois, today was accused that several of their food products contain a substance known as Gloactimate, which may be harmful to people's health. Gloactimate is an additive in processed foods and is used to increase the shelf life of foods. A recent series of studies found that Gloactimate raises "bad" cholesterol, lowers "good" cholesterol, and increases risk for heart disease.

Corporate Response: The Company Allows an Independent Investigation

The Locks Corporation announced that it is confident in its adherence to government standards regarding Gloactimate and would allow independent investigators into any of their nationwide locations to test their products. The company emphasized that with food products in stores and warehouses throughout the country, there would be no feasible way the Gloactimate would go undetected.

An independent group of scientists from the Advanced Science Institute (ASI) has offered to conduct an independent investigation. ASI has formed a team of investigators that includes physicians, nutritionists, chemists, health inspectors and several senior members of ASI. The Locks Corporation has agreed to allow ASI access to any of its facilities.

Now, please use the following questions to rate the Locks Corporation: *(Circle only one number for each rating)*:

Bad **Good**
1 --------- 2 --------- 3 --------- 4 ---------5 --------- 6 ---------7 --------- 8 --------- 9

Unethical **Ethical**
1 --------- 2 --------- 3 --------- 4 ---------5 --------- 6 ---------7 --------- 8 --------- 9

Immoral **Moral**
1 --------- 2 --------- 3 --------- 4 ---------5 --------- 6 ---------7 --------- 8 --------- 9

Irresponsible **Responsible**
1 --------- 2 --------- 3 --------- 4 ---------5 --------- 6 ---------7 --------- 8 --------- 9

Deceitful **Honest**
1 --------- 2 --------- 3 --------- 4 ---------5 --------- 6 ---------7 --------- 8 --------- 9

Guilty **Innocent**
1 --------- 2 --------- 3 --------- 4 ---------5 --------- 6 ---------7 --------- 8 --------- 9

NOTE: This is the "presumption of guilt study", condition *3* of 4. The study uses a between-subjects design with random assignment to one of the four conditions.

Chicago, Ill., December 2, 2013 – The Locks Corporation, based in Rockford, Illinois, today was accused that several of their food products contain a substance known as Gloactimate, which may be harmful to people's health. Gloactimate is an additive in processed foods and is used to increase the shelf life of foods. A recent series of studies found that Gloactimate raises "bad" cholesterol, lowers "good" cholesterol, and increases risk for heart disease.

Corporate Response: The Company Allows an Independent Investigation

The Locks Corporation announced that it is confident in its adherence to government standards regarding Gloactimate and would allow independent investigators into any of their nationwide locations to test their products. The company emphasized that with food products in stores and warehouses throughout the country, there would be no feasible way the Gloactimate would go undetected.

An independent group of scientists from the Advanced Science Institute (ASI) has conducted an independent investigation. ASI formed a team of investigators that included physicians, nutritionists, chemists, health inspectors and several senior members of ASI. The Locks Corporation agreed to allow ASI access into any of its facilities. This group of scientists has concluded that the food from the Locks Corporation **does not** contain Gloactimate.

Now, please use the following questions to rate the Locks Corporation: *(Circle only one number for each rating)*:

Bad **Good**
1 --------- 2 --------- 3 --------- 4 ---------5 --------- 6 ---------7 --------- 8 --------- 9

Unethical **Ethical**
1 --------- 2 --------- 3 --------- 4 ---------5 --------- 6 ---------7 --------- 8 --------- 9

Immoral **Moral**
1 --------- 2 --------- 3 --------- 4 ---------5 --------- 6 ---------7 --------- 8 --------- 9

Irresponsible **Responsible**
1 --------- 2 --------- 3 --------- 4 ---------5 --------- 6 ---------7 --------- 8 --------- 9

Deceitful **Honest**
1 --------- 2 --------- 3 --------- 4 ---------5 --------- 6 ---------7 --------- 8 --------- 9

Guilty **Innocent**
1 --------- 2 --------- 3 --------- 4 ---------5 --------- 6 ---------7 --------- 8 --------- 9

NOTE: This is the "presumption of guilt study", condition *4* of 4. The study uses a between-subjects design with random assignment to one of the four conditions.

Chicago, Ill., December 2, 2013 – The Locks Corporation, based in Rockford, Illinois, today was accused that several of their food products contain a substance known as Gloactimate, which may be harmful to people's health. Gloactimate is an additive in processed foods and is used to increase the shelf life of foods. A recent series of studies found that Gloactimate raises "bad" cholesterol, lowers "good" cholesterol, and increases risk for heart disease.

Corporate Response: The Company Allows an Independent Investigation

The Locks Corporation announced that it is confident in its adherence to government standards regarding Gloactimate and would allow independent investigators into any of their nationwide locations to test their products. The company emphasized that with food products in stores and warehouses throughout the country, there would be no feasible way the Gloactimate would go undetected.

An independent group of scientists from the Advanced Science Institute (ASI) has conducted an independent investigation. ASI formed a team of investigators that included physicians, nutritionists, chemists, health inspectors and several senior members of ASI. The Locks Corporation agreed to allow ASI access into any of its facilities. This group of scientists has concluded that the food from the Locks Corporation **does** contain Gloactimate.

Now, please use the following questions to rate the Locks Corporation: *(Circle only one number for each rating)*:

Bad **Good**
1 --------- 2 --------- 3 --------- 4 ---------5 --------- 6 ---------7 --------- 8 --------- 9

Unethical **Ethical**
1 --------- 2 --------- 3 --------- 4 ---------5 --------- 6 ---------7 --------- 8 --------- 9

Immoral **Moral**
1 --------- 2 --------- 3 --------- 4 ---------5 --------- 6 ---------7 --------- 8 --------- 9

Irresponsible **Responsible**
1 --------- 2 --------- 3 --------- 4 ---------5 --------- 6 ---------7 --------- 8 --------- 9

Deceitful **Honest**
1 --------- 2 --------- 3 --------- 4 ---------5 --------- 6 ---------7 --------- 8 --------- 9

Guilty **Innocent**
1 --------- 2 --------- 3 --------- 4 ---------5 --------- 6 ---------7 --------- 8 --------- 9

NOTE: This is the "moral inversion study", condition *1* of 4. The study uses a between-subjects design with random assignment to one of the four conditions.

Farrell Incorporated is a multi-billion dollar home furnishing company.

Farrell Incorporated is:

Manipulative NOT manipulative
1 --------- 2 --------- 3 --------- 4 ---------5 --------- 6 ---------7 --------- 8 --------- 9

Untrustworthy Trustworthy
1 --------- 2 --------- 3 --------- 4 ---------5 --------- 6 ---------7 --------- 8 --------- 9

Bad Good
1 --------- 2 --------- 3 --------- 4 ---------5 --------- 6 ---------7 --------- 8 --------- 9

Immoral Moral
1 --------- 2 --------- 3 --------- 4 ---------5 --------- 6 ---------7 --------- 8 --------- 9

NOTE: This is the "moral inversion study", condition *2* of 4. The study uses a between-subjects design with random assignment to one of the four conditions.

Farrell Incorporated is a multi-billion dollar home furnishing company.

Recently the company donated 200,000 dollars to a charity for cancer research.

Farrell Incorporated is:

Manipulative NOT manipulative
1 --------- 2 --------- 3 --------- 4 ---------5 --------- 6 ---------7 --------- 8 --------- 9

Untrustworthy Trustworthy
1 --------- 2 --------- 3 --------- 4 ---------5 --------- 6 ---------7 --------- 8 --------- 9

Bad Good
1 --------- 2 --------- 3 --------- 4 ---------5 --------- 6 ---------7 --------- 8 --------- 9

Immoral Moral
1 --------- 2 --------- 3 --------- 4 ---------5 --------- 6 ---------7 --------- 8 --------- 9

NOTE: This is the "moral inversion study", condition *3* of 4. The study uses a between-subjects design with random assignment to one of the four conditions.

Farrell Incorporated is a multi-billion dollar home furnishing company.

Recently the company donated $200,000 dollars to a charity for cancer research.

The company then spent 2 million dollars on an advertising campaign about its donation for cancer research.

Farrell Incorporated is:

Manipulative NOT manipulative
1 --------- 2 --------- 3 --------- 4 ---------5 --------- 6 ---------7 --------- 8 --------- 9

Untrustworthy Trustworthy
1 --------- 2 --------- 3 --------- 4 ---------5 --------- 6 ---------7 --------- 8 --------- 9

Bad Good
1 --------- 2 --------- 3 --------- 4 ---------5 --------- 6 ---------7 --------- 8 --------- 9

Immoral Moral
1 --------- 2 --------- 3 --------- 4 ---------5 --------- 6 ---------7 --------- 8 --------- 9

NOTE: This is the "moral inversion study", condition *4* of 4. The study uses a between-subjects design with random assignment to one of the four conditions.

Farrell Incorporated is a multi-billion dollar home furnishing company.

Recently the company donated 200,000 dollars to a charity for cancer research.

The company also spent 2 million dollars on an advertising campaign about its home furnishings.

Farrell Incorporated is:

Manipulative NOT manipulative
1 --------- 2 --------- 3 --------- 4 ---------5 --------- 6 ---------7 --------- 8 --------- 9

Untrustworthy Trustworthy
1 --------- 2 --------- 3 --------- 4 ---------5 --------- 6 ---------7 --------- 8 --------- 9

Bad Good
1 --------- 2 --------- 3 --------- 4 ---------5 --------- 6 ---------7 --------- 8 --------- 9

Immoral Moral
1 --------- 2 --------- 3 --------- 4 ---------5 --------- 6 ---------7 --------- 8 --------- 9

NOTE: This is the "higher standard" study. This is condition *1* of 6 between-subjects conditions. Please further note that the sixth DV item says "invest money" in conditions 1-3 and "donate" in conditions 4-6; thus the DV items are not perfectly identical across conditions.

Instructions: Please read the hiring scenario below and then answer the questions.

The Jens Shoes Corporation is deciding between two candidates for President.

Lisa has an MBA from Harvard Business School and eight years of managerial experience at a sneakers company. She was promoted after developing successful partnerships with several shoe companies that cut overhead and administrative costs substantially. As part of her contract, Lisa is requesting a salary of $400,000 a year.

Karen has an MBA from Ross Business School at the University of Michigan and eleven years of managerial experience at an online shoe company. She was promoted after designing a new capital campaign that raised significantly more investments than her predecessor. As part of her proposed contract, Karen is asking for a salary of $400,000.

Please use the scale below to indicate whether the following characteristics are more true of Lisa or Karen.

Definitely Lisa					*Definitely Karen*	
1	2	3	4	5	6	7

___Who is a more responsible person?

___Who is probably a more morally upstanding human being?

___Who do you predict will make more responsible decisions as leader?

___Who do you predict will act in the best interests of the organization?

___Who is a more selfish person?

___Who would you invest money with?

___Who would you hire as President?

NOTE: This is the "higher standard" study. This is condition *2* of 6 between-subjects conditions. Please further note that the sixth DV item says "invest money" in conditions 1-3 and "donate" in conditions 4-6.

Instructions: Please read the hiring scenario below and then answer the questions.

The Jens Shoes Corporation is deciding between two candidates for President.

Lisa has an MBA from Harvard Business School and eight years of managerial experience at a sneakers company. She was promoted after developing successful partnerships with several shoe companies that cut overhead and administrative costs substantially. As part of her contract, Lisa is requesting a salary of $400,000 a year.

Karen has an MBA from Ross Business School at the University of Michigan and eleven years of managerial experience at an online shoe company. She was promoted after designing a new capital campaign that raised significantly more investments than her predecessor. As part of her proposed contract, Karen is asking for a salary of $350,000 plus $50,000 per year for rental of a chauffeur-driven limo on the weekends.

Please use the scale below to indicate whether the following characteristics are more true of Lisa or Karen.

Definitely Lisa					*Definitely Karen*	
1	2	3	4	5	6	7

___Who is a more responsible person?

___Who is probably a more morally upstanding human being?

___Who do you predict will make more responsible decisions as leader?

___Who do you predict will act in the best interests of the organization?

___Who is a more selfish person?

___Who would you invest money with?

___Who would you hire as President?

NOTE: This is the "higher standard" study. This is condition *3* of 6 between-subjects conditions. Please further note that the sixth DV item says "invest money" in conditions 1-3 and "donate" in conditions 4-6.

Instructions: Please read the hiring scenario below and then answer the questions.

The Jens Shoes Corporation is deciding between two candidates for President.

Lisa has an MBA from Harvard Business School and eight years of managerial experience at a sneakers company. She was promoted after developing successful partnerships with several shoe companies that cut overhead and administrative costs substantially. As part of her contract, Lisa is requesting a salary of $400,000 a year.

Karen has an MBA from Ross Business School at the University of Michigan and eleven years of managerial experience at an online shoe company. She was promoted after designing a new capital campaign that raised significantly more investments than her predecessor. As part of her proposed contract, Karen is asking for a salary of $395,000 plus $5,000 per year for luxury water flown from Sweden.

Please use the scale below to indicate whether the following characteristics are more true of Lisa or Karen.

Definitely Lisa					*Definitely Karen*	
1	2	3	4	5	6	7

___Who is a more responsible person?

___Who is probably a more morally upstanding human being?

___Who do you predict will make more responsible decisions as leader?

___Who do you predict will act in the best interests of the organization?

___Who is a more selfish person?

___Who would you invest money with?

___Who would you hire as President?

NOTE: This is the "higher standard" study. This is condition *4* of 6 between-subjects conditions. Please further note that the sixth DV item says "invest money" in conditions 1-3 and "donate" in conditions 4-6.

Instructions: Please read the hiring scenario below and then answer the questions.

The Somalia Hunger Relief Charity is deciding between two candidates for President.

Lisa has an MBA from Harvard Business School and eight years of managerial experience at a children's non-profit. She was promoted after developing successful partnerships with several international charity agencies that cut overhead and administrative costs substantially. As part of her contract, Lisa is requesting a salary of $400,000 a year.

Karen has an MBA from Ross Business School at the University of Michigan and eleven years of managerial experience at an advocacy non-profit. She was promoted after designing a new fundraising campaign that raised significantly more donations than her predecessor. As part of her proposed contract, Karen is asking for a salary of $400,000.

Please use the scale below to indicate whether the following characteristics are more true of Lisa or Karen.

Definitely Lisa					*Definitely Karen*	
1	2	3	4	5	6	7

___Who is a more responsible person?

___Who is probably a more morally upstanding human being?

___Who do you predict will make more responsible decisions as leader?

___Who do you predict will act in the best interests of the organization?

___Who is a more selfish person?

___Who would you prefer to donate money with?

___Who would you hire as President?

NOTE: This is the "higher standard" study. This is condition *5* of 6 between-subjects conditions. Please further note that the sixth DV item says "invest money" in conditions 1-3 and "donate" in conditions 4-6.

Instructions: Please read the hiring scenario below and then answer the questions.

The Somalia Hunger Relief Charity is deciding between two candidates for President.

Lisa has an MBA from Harvard Business School and eight years of managerial experience at a children's non-profit. She was promoted after developing successful partnerships with several international charity agencies that cut overhead and administrative costs substantially. As part of her contract, Lisa is requesting a salary of $400,000 a year.

Karen has an MBA from Ross Business School at the University of Michigan and eleven years of managerial experience at an advocacy non-profit. She was promoted after designing a new fundraising campaign that raised significantly more donations than her predecessor. As part of her proposed contract, Karen is asking for a salary of $350,000 plus $50,000 per year for rental of a chauffeur-driven limo on the weekends.

Please use the scale below to indicate whether the following characteristics are more true of Lisa or Karen.

Definitely Lisa					*Definitely Karen*	
1	2	3	4	5	6	7

___Who is a more responsible person?

___Who is probably a more morally upstanding human being?

___Who do you predict will make more responsible decisions as leader?

___Who do you predict will act in the best interests of the organization?

___Who is a more selfish person?

___Who would you prefer to donate money with?

___Who would you hire as President?

NOTE: This is the "higher standard" study. This is condition *6* of 6 between-subjects conditions. Please further note that the sixth DV item says "invest money" in conditions 1-3 and "donate" in conditions 4-6.

Instructions: Please read the hiring scenario below and then answer the questions.

The Somalia Hunger Relief Charity is deciding between two candidates for President.

Lisa has an MBA from Harvard Business School and eight years of managerial experience at a children's non-profit. She was promoted after developing successful partnerships with several international charity agencies that cut overhead and administrative costs substantially. As part of her contract, Lisa is requesting a salary of $400,000 a year.

Karen has an MBA from Ross Business School at the University of Michigan and eleven years of managerial experience at an advocacy non-profit. She was promoted after designing a new fundraising campaign that raised significantly more donations than her predecessor. As part of her proposed contract, Karen is asking for a salary of $395,000 plus $5,000 per year for luxury water flown from Sweden.

Please use the scale below to indicate whether the following characteristics are more true of Lisa or Karen.

Definitely Lisa					*Definitely Karen*	
1	2	3	4	5	6	7

___Who is a more responsible person?

___Who is probably a more morally upstanding human being?

___Who do you predict will make more responsible decisions as leader?

___Who do you predict will act in the best interests of the organization?

___Who is a more selfish person?

___Who would you prefer to donate money with?

___Who would you hire as President?

NOTE: This is the "belief-act inconsistency study", condition *1* of 3. The study uses a between-subjects design with random assignment to one of the three conditions.

Bob Hill has worked for 20 years as an animal rights activist and president of the non-profit organization Furry Friends Forever (FFF), which advocates for the ethical treatment of domestic and wild animals. FFF works through public education, cruelty investigations, research, animal rescue, legislation, special events, celebrity involvement, and protest campaigns.

Recently, the Associated Press news service reported that Hill had participated in a wild game hunting safari in South Africa. The report indicated that this is the fourth big game hunting safari that Hill has done in the last five years. Below is a picture that accompanied the press release, showing Hill with a Kudu antelope that he shot down with a .338 Winchester Magnum hunting rifle.

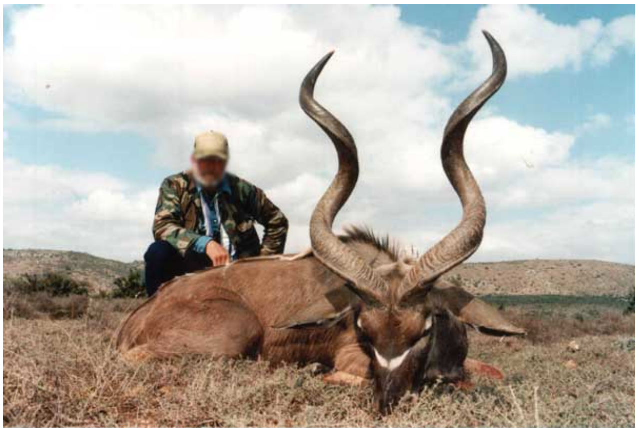

(1) How morally blameworthy or morally praiseworthy do you find Bob as a person?

–5	–4	–3	–2	–1	0	1	2	3	4	5

Extremely Blameworthy *Extremely Praiseworthy*

(2) How much warmth or coldness do you feel personally towards Bob?

–5	–4	–3	–2	–1	0	1	2	3	4	5

Incredibly cold *Incredibly warm*

(3) How trustworthy do you personally find Bob to be?

–5	–4	–3	–2	–1	0	1	2	3	4	5

Incredibly untrustworthy *Incredibly trustworthy*

(4) Do you find Bob to be a hypocrite?

0	1	2	3	4	5	6	7	8	9	10

Not at all *Definitely*

(5) How do you feel about the activity of hunting wild (non-endangered) animals?

–5	–4	–3	–2	–1	0	1	2	3	4	5

Very Wrong *Perfectly Okay*

NOTE: This is the "belief-act inconsistency study", condition *2* of 3. The study uses a between-subjects design with random assignment to one of the three conditions.

Bob Hill has worked for 20 years as an avid hunter and president of the American Big Game Hunters Association (ABGA), which advocates for big game trophy hunting throughout North America and the world. ABGA serves the hunting community through the sharing of experiences, knowledge and technology, promoting the education of youth in securing the future of the hunting tradition, and extending the goodwill of members through community outreach.

Recently, the Associated Press news service reported that Hill had participated in a wild game hunting safari in South Africa. The report indicated that this is the fourth big game hunting safari that Hill has done in the last five years. Below is a picture that accompanied the press release, showing Hill with a Kudu antelope that he shot down with a .338 Winchester Magnum hunting rifle.

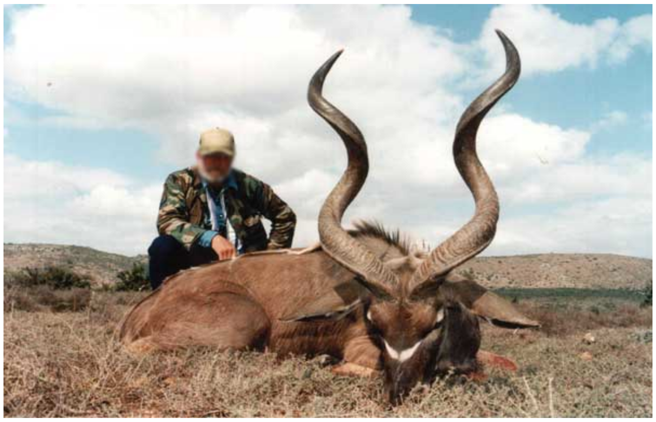

(1) How morally blameworthy or morally praiseworthy do you find Bob as a person?

–5 –4 –3 –2 –1 0 1 2 3 4 5

Extremely Blameworthy *Extremely Praiseworthy*

(2) How much warmth or coldness do you feel personally towards Bob?

–5 –4 –3 –2 –1 0 1 2 3 4 5

Incredibly cold *Incredibly warm*

(3) How trustworthy do you personally find Bob to be?

–5 –4 –3 –2 –1 0 1 2 3 4 5

Incredibly untrustworthy *Incredibly trustworthy*

(4) Do you find Bob to be a hypocrite?

0 1 2 3 4 5 6 7 8 9 10

Not at all *Definitely*

(5) How do you feel about the activity of hunting wild (non-endangered) animals?

–5 –4 –3 –2 –1 0 1 2 3 4 5

Very Wrong *Perfectly Okay*

NOTE: This is the "belief-act inconsistency study", condition *3* of 3. The study uses a between-subjects design with random assignment to one of the three conditions.

Bob Hill has worked for 20 years as a human right activist and president of doctors without borders (DWB), which provides medical aid in nearly 60 countries to people whose survival is threatened by violence, neglect, or catastrophe, primarily due to armed conflict, epidemics, malnutrition, exclusion from health care, or natural disasters. DWB provides independent, impartial assistance to those most in need. DWB is committed to bringing quality medical care to people caught in crisis regardless of race, religion, or political affiliation.

Recently, the Associated Press news service reported that Hill had participated in a wild game hunting safari in South Africa. The report indicated that this is the fourth big game hunting safari that Hill has done in the last five years. Below is a picture that accompanied the press release, showing Hill with a Kudu antelope that he shot down with a .338 Winchester Magnum hunting rifle.

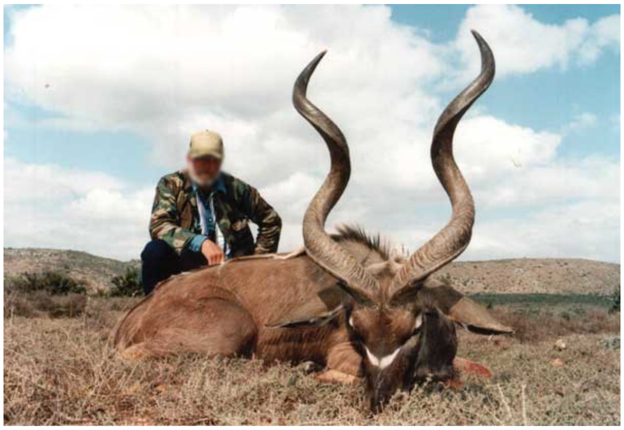

(1) How morally blameworthy or morally praiseworthy do you find Bob as a person?

–5 –4 –3 –2 –1 0 1 2 3 4 5

Extremely Blameworthy *Extremely Praiseworthy*

(2) How much warmth or coldness do you feel personally towards Bob?

–5 –4 –3 –2 –1 0 1 2 3 4 5

Incredibly cold *Incredibly warm*

(3) How trustworthy do you personally find Bob to be?

–5 –4 –3 –2 –1 0 1 2 3 4 5

Incredibly untrustworthy *Incredibly trustworthy*

(4) Do you find Bob to be a hypocrite?

0 1 2 3 4 5 6 7 8 9 10

Not at all *Definitely*

(5) How do you feel about the activity of hunting wild (non-endangered) animals?

–5 –4 –3 –2 –1 0 1 2 3 4 5

Very Wrong *Perfectly Okay*

NOTE: These are the materials for the "moral cliff" study. **Each participant does both of these scenarios+follow-up DVs, with page order counterbalanced between-subjects**.

A cosmetics company hires a model to appear in an advertisement for their skin cream. She is one in a million in terms of the beauty of her skin. The skin cream advertisement with the model appears in magazines and on billboards all over the world.

How accurately or inaccurately does the company's advertisement portray the effectiveness of their skin cream?

extremely inaccurately 1 2 3 4 5 6 7 extremely accurately

Does the company's advertisement create a correct impression of how well their skin cream works?

extremely incorrect 1 2 3 4 5 6 7 extremely correct

Is this advertisement dishonest?

not at all dishonest 1 2 3 4 5 6 7 extremely dishonest

Is this advertisement fraudulent?

not at all fraudulent 1 2 3 4 5 6 7 extremely fraudulent

Is this a case of false advertising?

Definitely false advertising 1 2 3 4 5 6 7 Definitely truthful advertising

Should this advertisement be banned?

Definitely not 1 2 3 4 5 6 7 Definitely yes

Should the company be fined money for running this ad?

Definitely not 1 2 3 4 5 6 7 Definitely yes

Did the company intentionally misrepresent their product to consumers?

Definitely not 1 2 3 4 5 6 7 Definitely yes

How easy or difficult is it for the company to justify their behavior to themselves as legitimate?

Extremely difficult 1 2 3 4 5 6 7 Extremely easy

A cosmetics company hires a model to appear in an advertisement for their skin cream. She is one in a thousand in terms of the beauty of her skin. An artist who works for the cosmetics company then uses Photoshop to make her skin appear one in a million in terms of beauty. The skin cream advertisement with the model appears in magazines and on billboards all over the world.

How accurately or inaccurately does the company's advertisement portray the effectiveness of their skin cream?

extremely inaccurately	1	2	3	4	5	6	7	extremely accurately

Does the company's advertisement create a correct impression of how well their skin cream works?

extremely incorrect	1	2	3	4	5	6	7	extremely correct

Is this advertisement dishonest?

not at all dishonest	1	2	3	4	5	6	7	extremely dishonest

Is this advertisement fraudulent?

not at all fraudulent	1	2	3	4	5	6	7	extremely fraudulent

Is this a case of false advertising?

Definitely false advertising	1	2	3	4	5	6	7	Definitely truthful advertising

Should this advertisement be banned?

Definitely not	1	2	3	4	5	6	7	Definitely yes

Should the company be fined money for running this ad?

Definitely not	1	2	3	4	5	6	7	Definitely yes

Did the company intentionally misrepresent their product to consumers?

Definitely not	1	2	3	4	5	6	7	Definitely yes

How easy or difficult is it for the company to justify their behavior to themselves as legitimate?

Extremely difficult	1	2	3	4	5	6	7	Extremely easy

NOTE: This is the "cold-hearted prosociality study." This is *1* of 2 between subjects conditions.

INSTRUCTIONS: Please read the paragraphs about the individuals below and answer the questions that come after.

Karen works as an assistant in a medical center that does cancer research. The laboratory develops drugs that improve survival rates for people stricken with breast cancer. As part of Karen's job, she places mice in a special cage, and then exposes them to radiation in order to give them tumors. Once the mice develop tumors, it is Karen's job to give them injections of experimental cancer drugs.

Lisa works as an assistant at a store for expensive pets. The store sells pet gerbils to wealthy individuals and families. As part of Lisa's job, she places gerbils in a special bathtub, and then exposes them to a grooming shampoo in order to make sure they look nice for the customers. Once the gerbils are groomed, it is Lisa's job to tie a bow on them.

Please use this scale for the following items:

Definitely Karen					Definitely Lisa	
1	2	3	4	5	6	7

______ Whose actions benefit society more?
______ Whose job duties make a more moral contribution to society?
______ Whose job is more morally praiseworthy?
______ Whose actions make a greater moral contribution to the world?

Who is more likely to have the following traits?

Definitely Karen					Definitely Lisa	
1	2	3	4	5	6	7

______ Caring
______ Cold-hearted
______ Aggressive
______ Kind-hearted

In my opinion, testing cancer drugs on mice is:

Definitely wrong			*not sure*			*Definitely OK*
1	2	3	4	5	6	7

NOTE: This is the "cold-hearted prosociality study." This is *2* of 2 between subjects conditions.

INSTRUCTIONS: Please read the paragraphs about the individuals below and answer the questions that come after.

Lisa works as an assistant in a medical center that does cancer research. The laboratory develops drugs that improve survival rates for people stricken with breast cancer. As part of Lisa's job, she places mice in a special cage, and then exposes them to radiation in order to give them tumors. Once the mice develop tumors, it is Lisa's job to give them injections of experimental cancer drugs.

Karen works as an assistant at a store for expensive pets. The store sells pet gerbils to wealthy individuals and families. As part of Karen's job, she places gerbils in a special bathtub, and then exposes them to a grooming shampoo in order to make sure they look nice for the customers. Once the gerbils are groomed, it is Karen's job to tie a bow on them.

Please use this scale for the following items:

Definitely Karen Definitely Lisa
1 2 3 4 5 6 7

_____ Whose actions benefit society more?
_____ Whose job duties make a more moral contribution to society?
_____ Whose job is more morally praiseworthy?
_____ Whose actions make a greater moral contribution to the world?

Who is more likely to have the following traits?

Definitely Karen Definitely Lisa
1 2 3 4 5 6 7

_____ Caring
_____ Cold-hearted
_____ Aggressive
_____ Kind-hearted

In my opinion, testing cancer drugs on mice is:
Definitely wrong *not sure* *Definitely OK*
1 2 3 4 5 6 7

NOTE: These are the materials for the “Bad Tipper” study. This is *1* of 2 between-subjects conditions.

Instructions: We would now like you to read about a person named Jack.

Jack is eating dinner at a restaurant. The expected gratuity for his bill would be approximately $15. Satisfied with his meal and service, Jack places a few bills on the table (totaling to $14) before he leaves.

Do you think that Jack is probably a disrespectful person?

Not at all Definitely
1 2 3 4 5 6 7

Do you think that Jack probably has a good moral conscience?

Not at all Definitely
1 2 3 4 5 6 7

Is Jack the type of person that you would want as a close friend?

Not at all Definitely
1 2 3 4 5 6 7

Would you say that in general, Jack is a good person?

Not at all Definitely
1 2 3 4 5 6 7

Strictly speaking, how blameworthy was Jack's behavior?

Not at all blameworthy Completely blameworthy
1 2 3 4 5 6 7

Do you think this behavior tells you a lot or a little about Jack's personality?

Says nothing about Jack Says a lot about Jack
1 2 3 4 5 6 7

NOTE: These are the materials for the "Bad Tipper" study. This is *2* of 2 between-subjects conditions.

Instructions: We would now like you to read about a person named Jack.

Jack is eating dinner at a restaurant. The expected gratuity for his bill would be approximately $15. Satisfied with his meal and service, Jack places a large bag of pennies on the table (totaling to $15) before he leaves.

Do you think that Jack is probably a disrespectful person?

Not at all Definitely
1 2 3 4 5 6 7

Do you think that Jack probably has a good moral conscience?

Not at all Definitely
1 2 3 4 5 6 7

Is Jack the type of person that you would want as a close friend?

Not at all Definitely
1 2 3 4 5 6 7

Would you say that in general, Jack is a good person?

Not at all Definitely
1 2 3 4 5 6 7

Strictly speaking, how blameworthy was Jack's behavior?

Not at all blameworthy Completely blameworthy
1 2 3 4 5 6 7

Do you think this behavior tells you a lot or a little about Jack's personality?

Says nothing about Jack Says a lot about Jack
1 2 3 4 5 6 7

NOTE: This is the "bigot misanthrope" study. This is *1* of 2 between subjects conditions.

Instructions: We would like to get your impressions about two CEOs, Robert and John, who work at similar but different companies.

John is a CEO at Company X. John does not say "hi" or engage in friendly small talk with any of his employees. When an employee says "hi", John never responds.

Robert is a CEO at Company Y. Robert always says "hi" and engages in friendly small talk with his White employees. But when an African American employee says "hi," Robert never responds.

(At both companies, about 80% of co-workers are White, and about 20% are African American)

Who is a more immoral person?

Definitely John / Definitely Robert

1 2 3 4 5 6 7

Who is more morally blameworthy as a person?

Definitely John / Definitely Robert

1 2 3 4 5 6 7

Which person's action tells you more about their moral character?

Definitely John / Definitely Robert

1 2 3 4 5 6 7

Whose behavior towards their co-worker tells you more about their personality?

Definitely John / Definitely Robert

1 2 3 4 5 6 7

NOTE: This is the “bigot misanthrope” study. This is *2* of 2 between subjects conditions.

Instructions: We would like to get your impressions about two CEOs, Robert and John, who work at similar but different companies.

Robert is a CEO at Company X. Robert does not say "hi" or engage in friendly small talk with any of his employees. When an employee says "hi", Robert never responds.

John is a CEO at Company Y. John always says "hi" and engages in friendly small talk with his White employees. But when an African American employee says "hi," John never responds.

(At both companies, about 80% of co-workers are White, and about 20% are African American)

Who is a more immoral person?

Definitely John Definitely Robert

1 2 3 4 5 6 7

Who is more morally blameworthy as a person?

Definitely John Definitely Robert

1 2 3 4 5 6 7

Which person's action tells you more about their moral character?

Definitely John Definitely Robert

1 2 3 4 5 6 7

Whose behavior towards their co-worker tells you more about their personality?

Definitely John Definitely Robert

1 2 3 4 5 6 7

NOTE: This is the "intuitive economics study". This is *1* of 2 between-subjects conditions (4 pages of questions).

Are high taxes fair or unfair?
Very FAIR Neutral Very UNFAIR
1 2 3 4 5 6 7

Are high taxes good or bad for the economy?
Very bad Neither Very good
1 2 3 4 5 6 7

Is the federal deficit fair or unfair?
Very FAIR Neutral Very UNFAIR
1 2 3 4 5 6 7

Is the federal deficit good or bad for the economy?
Very bad Neither Very good
1 2 3 4 5 6 7

Is foreign aid fair or unfair?
Very FAIR Neutral Very UNFAIR
1 2 3 4 5 6 7

Is foreign aid good or bad for the economy?
Very bad Neither Very good
1 2 3 4 5 6 7

Is the entrance of women into the workforce fair or unfair?
Very FAIR Neutral Very UNFAIR
1 2 3 4 5 6 7

Is the entrance of women into the workforce good or bad for the economy?
Very bad Neither Very good
1 2 3 4 5 6 7

Is the increased use of technology in the workplace fair or unfair?
Very FAIR Neutral Very UNFAIR
1 2 3 4 5 6 7

Is the increased use of technology in the workplace good or bad for the economy?
Very bad Neither Very good
1 2 3 4 5 6 7

Are trade agreements between the U.S. and other countries fair or unfair?
Very FAIR Neutral Very UNFAIR
1 2 3 4 5 6 7

Are trade agreements between the U.S. and other countries good or bad for the economy?
Very bad Neither Very good
1 2 3 4 5 6 7

Is companies downsizing fair or unfair?
Very FAIR Neutral Very UNFAIR
1 2 3 4 5 6 7

Is companies downsizing good or bad for the economy?
Very bad Neither Very good
1 2 3 4 5 6 7

Is companies not investing in education and job training fair or unfair?
Very FAIR Neutral Very UNFAIR
1 2 3 4 5 6 7

Is companies not investing in education and job training good or bad for the economy?
Very bad Neither Very good
1 2 3 4 5 6 7

Are tax cuts fair or unfair?
Very FAIR Neutral Very UNFAIR
1 2 3 4 5 6 7

Are tax cuts good or bad for the economy?
Very bad Neither Very good
1 2 3 4 5 6 7

Is a lack of business productivity fair or unfair?
Very FAIR Neutral Very UNFAIR
1 2 3 4 5 6 7

Is a lack of business productivity good or bad for the economy?
Very bad Neither Very good
1 2 3 4 5 6 7

Is technology displacing workers fair or unfair?
Very FAIR Neutral Very UNFAIR
1 2 3 4 5 6 7

Is technology displacing workers good or bad for the economy?
Very bad Neither Very good
1 2 3 4 5 6 7

Is companies sending jobs overseas fair or unfair?
Very FAIR Neutral Very UNFAIR
1 2 3 4 5 6 7

Is companies sending jobs overseas good or bad for the economy?
Very bad Neither Very good
1 2 3 4 5 6 7

Is people not saving their money fair or unfair?
Very FAIR Neutral Very UNFAIR
1 2 3 4 5 6 7

Is people not saving their money good or bad for the economy?
Very bad Neither Very good
1 2 3 4 5 6 7

Are high business profits fair or unfair?
Very FAIR Neutral Very UNFAIR
1 2 3 4 5 6 7

Are high business profits good or bad for the economy?
Very bad Neither Very good
1 2 3 4 5 6 7

Are the salaries of top (corporate) executives fair or unfair?
Very FAIR Neutral Very UNFAIR
1 2 3 4 5 6 7

Are the salaries of top (corporate) executives good or bad for the economy?
Very bad Neither Very good
1 2 3 4 5 6 7

Is affirmative action fair or unfair?
Very FAIR Neutral Very UNFAIR
1 2 3 4 5 6 7

Is affirmative action good or bad for the economy?
Very bad Neither Very good
1 2 3 4 5 6 7

Is people not valuing hard work fair or unfair?

Very FAIR Neutral Very UNFAIR

1 2 3 4 5 6 7

Is people not valuing hard work good or bad for the economy?

Very bad Neither Very good

1 2 3 4 5 6 7

Is government regulation of business fair or unfair?

Very FAIR Neutral Very UNFAIR

1 2 3 4 5 6 7

Is government regulation of business good or bad for the economy?

Very bad Neither Very good

1 2 3 4 5 6 7

Are illegal immigrants fair or unfair?

Very FAIR Neutral Very UNFAIR

1 2 3 4 5 6 7

Are illegal immigrants good or bad for the economy?

Very bad Neither Very good

1 2 3 4 5 6 7

Are tax breaks for business fair or unfair?

Very FAIR Neutral Very UNFAIR

1 2 3 4 5 6 7

Are tax breaks for business good or bad for the economy?

Very bad Neither Very good

1 2 3 4 5 6 7

Is welfare fair or unfair?

Very FAIR Neutral Very UNFAIR

1 2 3 4 5 6 7

Is welfare good or bad for the economy?

Very bad Neither Very good

1 2 3 4 5 6 7

NOTE: This is the "intuitive economics study". This is *2* of 2 between-subjects conditions (4 pages of questions).

Are high taxes fair or unfair?

Very UNFAIR Neutral Very FAIR

1 2 3 4 5 6 7

Are high taxes good or bad for the economy?

Very bad Neither Very good

1 2 3 4 5 6 7

Is the federal deficit fair or unfair?

Very UNFAIR Neutral Very FAIR

1 2 3 4 5 6 7

Is the federal deficit good or bad for the economy?

Very bad Neither Very good

1 2 3 4 5 6 7

Is foreign aid fair or unfair?

Very UNFAIR Neutral Very FAIR

1 2 3 4 5 6 7

Is foreign aid good or bad for the economy?

Very bad Neither Very good

1 2 3 4 5 6 7

Is the entrance of women into the workforce fair or unfair?

Very UNFAIR Neutral Very FAIR

1 2 3 4 5 6 7

Is the entrance of women into the workforce good or bad for the economy?

Very bad Neither Very good

1 2 3 4 5 6 7

Is the increased use of technology in the workplace fair or unfair?

Very UNFAIR Neutral Very FAIR

1 2 3 4 5 6 7

Is the increased use of technology in the workplace good or bad for the economy?

Very bad Neither Very good

1 2 3 4 5 6 7

Are trade agreements between the U.S. and other countries fair or unfair?

Very UNFAIR Neutral Very FAIR

1 2 3 4 5 6 7

Are trade agreements between the U.S. and other countries good or bad for the economy?

Very bad Neither Very good

1 2 3 4 5 6 7

Is companies downsizing fair or unfair?

Very UNFAIR Neutral Very FAIR

1 2 3 4 5 6 7

Is companies downsizing good or bad for the economy?

Very bad Neither Very good

1 2 3 4 5 6 7

Is companies not investing in education and job training fair or unfair?

Very UNFAIR Neutral Very FAIR

1 2 3 4 5 6 7

Is companies not investing in education and job training good or bad for the economy?

Very bad Neither Very good

1 2 3 4 5 6 7

Are tax cuts fair or unfair?

Very UNFAIR Neutral Very FAIR

1 2 3 4 5 6 7

Are tax cuts good or bad for the economy?

Very bad Neither Very good

1 2 3 4 5 6 7

Is a lack of business productivity fair or unfair?

Very UNFAIR Neutral Very FAIR

1 2 3 4 5 6 7

Is a lack of business productivity good or bad for the economy?

Very bad Neither Very good

1 2 3 4 5 6 7

Is technology displacing workers fair or unfair?

Very UNFAIR Neutral Very FAIR

1 2 3 4 5 6 7

Is technology displacing workers good or bad for the economy?

Very bad			Neither			Very good
1	2	3	4	5	6	7

Is companies sending jobs overseas fair or unfair?

Very UNFAIR			Neutral			Very FAIR
1	2	3	4	5	6	7

Is companies sending jobs overseas good or bad for the economy?

Very bad			Neither			Very good
1	2	3	4	5	6	7

Is people not saving their money fair or unfair?

Very UNFAIR			Neutral			Very FAIR
1	2	3	4	5	6	7

Is people not saving their money good or bad for the economy?

Very bad			Neither			Very good
1	2	3	4	5	6	7

Are high business profits fair or unfair?

Very UNFAIR			Neutral			Very FAIR
1	2	3	4	5	6	7

Are high business profits good or bad for the economy?

Very bad			Neither			Very good
1	2	3	4	5	6	7

Are the salaries of top (corporate) executives fair or unfair?

Very UNFAIR			Neutral			Very FAIR
1	2	3	4	5	6	7

Are the salaries of top (corporate) executives good or bad for the economy?

Very bad			Neither			Very good
1	2	3	4	5	6	7

Is affirmative action fair or unfair?

Very UNFAIR			Neutral			Very FAIR
1	2	3	4	5	6	7

Is affirmative action good or bad for the economy?

Very bad			Neither			Very good
1	2	3	4	5	6	7

Is people not valuing hard work fair or unfair?

Very UNFAIR			Neutral			Very FAIR
1	2	3	4	5	6	7

Is people not valuing hard work good or bad for the economy?

Very bad			Neither			Very good
1	2	3	4	5	6	7

Is government regulation of business fair or unfair?

Very UNFAIR			Neutral			Very FAIR
1	2	3	4	5	6	7

Is government regulation of business good or bad for the economy?

Very bad			Neither			Very good
1	2	3	4	5	6	7

Are illegal immigrants fair or unfair?

Very UNFAIR			Neutral			Very FAIR
1	2	3	4	5	6	7

Are illegal immigrants good or bad for the economy?

Very bad			Neither			Very good
1	2	3	4	5	6	7

Are tax breaks for business fair or unfair?

Very UNFAIR			Neutral			Very FAIR
1	2	3	4	5	6	7

Are tax breaks for business good or bad for the economy?

Very bad			Neither			Very good
1	2	3	4	5	6	7

Is welfare fair or unfair?

Very UNFAIR			Neutral			Very FAIR
1	2	3	4	5	6	7

Is welfare good or bad for the economy?

Very bad			Neither			Very good
1	2	3	4	5	6	7

NOTE: This is the "burn in hell" study. A descriptive one-page study, no conditions

Instructions:

Assume for a moment that hell exists. What percentage of people in the following categories would go to hell when they die?

Social Worker

% to hell _____

Drug Dealer

% to hell _____

Shoplifter

% to hell _____

Non-handicapped people who park in the handicapped spot

% to hell _____

Top Executives at big corporations

% to hell _____

People who sell prescription painkillers to addicts

% to hell _____

People who kick their dogs when they have a bad day

% to hell _____

Car Thieves

% to hell _____

Vandals who spray graffiti on public property

% to hell _____

NOTE: This is the demographic page to be administered with all studies

DEMOGRAPHICS

Please rate your political ideology on the following scale *(please circle one):*

- strongly left-wing
- moderately left-wing
- slightly left-wing
- moderate
- slightly right-wing,
- moderately right-wing
- strongly right-wing

My gender is *(please circle one)*: Male Female

What year were you born in? _______

What country were you born in? _________________

How many years of experience do you have with English? ______________

My ethnicity is (please circle one): White Asian Latino Black Indian
Other: ______________

The educational level of *your most highly educated parent* is:

- No formal education
- Completed primary/elementary school
- Completed secondary school/high school
- Some university/college
- Completed university/college degree
- Completed advanced degree.

My family's yearly income in U.S. dollars is about: $__________________

BEFORE TODAY, how many research studies had you participated in? _______________

Have you participating in any of these studies before? Yes No

If yes, please describe the study: _____________________________________

What city/town do you live in? ________________________________

What postal code do you live in? __________

Did you read the study materials carefully? Please be honest, you will be compensated for your time either way.

Yes No

Are you currently studying for a degree in business? Yes No

SUPPLEMENT 4: PRE-REGISTERED ANALYSIS PLAN

Pre-Registration Document 1:

Analytic approach

There is currently no single, fixed standard to evaluating replication results, and we will therefore apply a number of criteria to determine whether the replications successfully reproduced the original findings or not (see Brandt et al., 2014). These will include:

1. Whether the original and replication effects are in the same direction
2. Whether the replication effect was statistically significant
3. Whether meta-analyzing the original and replication effect results in a significant effect
4. Whether the replication effect size is significantly smaller than the original effect
5. Whether the replication effect size is too small to have been reliably detected in the original study (Simonsohn, 2013).

We will further employ Verhagen and Wagenmakers's (2014) suite of Bayesian tests for evaluating replications. These Bayesian tests parallel criteria 2, 3, and 4, and further test 6) whether the replication results suggest the original effect size or the null is more likely to be true.

In order to provide some additional assessments of the strength of evidence in the original studies, we will:

- Test for likelihood of Type M (Magnitude) and Type S (Sign) errors in the original studies (Gelman & Carlin, 2014).
- Use the V statistic to see if the inferences drawn from the original studies were better than guessing (Davis-Stober & Dana, 2014).

The final project report will feature a summary figure displaying the effect sizes observed in the original and replication labs (e.g., see Klein et al., 2014, Figure 1).

We will also conduct additional, more fine-grained comparisons of effect sizes based on the type of subject population in the replication. Specifically, we will compare original and replication effect sizes separately by:

- Whether the study came first vs. did not (to address the participant fatigue issue, and potential interference effects from running multiple studies together)
- Online data collections (MTurk, Moral Sense website, Your Morals Website) vs. university participants (undergraduate students, MBAs)
- Student population: psychology undergraduates vs. business undergraduates vs. MBAs
- Computer vs. paper-pencil administration of materials
- USA sample vs. non-USA sample
- Whether the original location vs. a different location was used for the replication. (For the "Presumption of guilt study," "Belief-act inconsistency study," "Intuitive economics

study," and "Burn in hell study" the original location was Northwestern University. For the other original studies it was Mechanical Turk)

We will be inclusive and test for all effects in each original study in the relevant replications.

Data collection

There will be a total of three survey packets containing a total of 10 original studies to be replicated.

We will conduct self-replications on Amazon's Mechanical Turk using each of the three packets. We will collect 1000 participants in each packet for a total of 3000 participants. Data will be checked at an early stage to make sure it is collecting properly, but data collection will continue until 1000 subjects have been run in each packet.

Each replication team will be asked to collect at least 100 participants in at least one survey packet (containing 3 to 4 brief studies each). Replication teams will have until March 1 to collect data.

Replication teams using paper-pencil administration (e.g., for on-campus surveys) will receive a packet with either 3 short studies or 1 longer study and be asked to collect at least 100 participants using their packet.

This process will be flexible, however, based on the resources of individual labs, and some replication teams may collect fewer (or more) subjects or replicate fewer (or more) studies.

If replication teams have difficulties in collecting enough data by the original March 1st deadline, or it appears there will be too much data to analyze and write it up by the original manuscript deadline of April 1st, we may extend the deadline for data collection to June 15th (i.e., the end of the semester at most participating universities) and analyze the data and write up the paper over the summer.

NOTE: A replication of six of the original studies at HEC Paris conducted by Anne-Laure Sellier took place prior to the creation of this document, and those data were also analyzed prior to the pre-registration. However we simply repeated all of the analyses from the original study in the HEC Paris replication dataset, as we will do for all replications.

Pre-Registration Document 2: Key effects to be tested from each study

Below, the dependent measure is always in quotes. All names are the same as in the Pipeline Project proposal. The key test is a between-subjects t-test unless otherwise indicated.

1. Bad tipper study: *"Person Judgments"* were worse in penny condition than in bills condition.
2. Belief act inconsistency study: *"Moral blameworthy-praiseworthy"* evaluations for Bob Hill were worse in the animal rights condition than in the big game hunting condition.
3. Burn in hell study: In the percentile estimates, *Corporate Executives* were rated as more likely to burn in hell than *Vandals*.
4. Cold hearted prosociality study: Medical researcher was rated worse on *"moral traits"* but better on *"moral actions"* than pet store assistant.
5. Presumption of guilt study: *"Company Evaluations"* in no-investigation-condition was the same as in company-found-guilty condition.
6. Bigot-misanthrope study: "*Person judgments*" for 'Bigot' were worse than for 'Misanthrope'.
7. Intuitive economics study: There was a positive correlation between *"Are high taxes good or bad for the economy?"* ratings and *"Are high taxes fair or unfair?"* ratings.
8. Moral inversion study: *"Company Evaluations"* were worse in the publicized-charity-condition than in the no-charity-condition.
9. Higher standard study: In the "Jen's Corporation" condition, *"Candidate Evaluations"* for the target candidate were NOT worse in the small perk condition than in the monetary-salary-only condition.
   In the "Somalia hunger relief" condition, *"Candidate Evaluations"* for the target candidate WERE worse in the small perk condition than in the monetary-salary-only condition.
10. Moral cliff study: Photoshop scenario was rated more *"Dishonest"* than the control scenario. This will be a within-subject comparison.

The final project report will feature a summary figure displaying the effect sizes observed in the original and replication labs (e.g., see Klein et al., 2014, Figure 1).

Addendum: Departures from preregistered analysis plan

We did not report the V statistic (Davis-Stober & Dana, 2014) for each of the original effects because Professors Davis-Stober and Dana determined the designs of the original studies were poorly suited to this statistical test.

We did not carry out the planned Type M and Type S error analyses (Gelman & Carlin, 2014) because both Professor Gelman and the Pipeline Projects' statistical experts expressed doubts about their suitability to the original studies targeted for replication.

Subject population (general population, MBA students, or undergraduates) turned out to be confounded with mode of study administration. All of the replications that recruited subjects from the general population collected the data online rather than in the laboratory, and paper-pencil questionnaires were only used with one undergraduate sample. We therefore analyzed only subject population as a potential moderator of replication results, not the method by which the study materials were administered to subjects. Due to the limited number of samples available, we also collapsed across student populations in our analyses, and simply compared results in the general population vs. student samples.

As stipulated in the pre-registration document, we exercised the option to continue data collection until June 15 to increase the sample sizes and statistical power of the replications. In a departure from the original plan, we further extended the deadline to July 15th to give a graduate student project coordinator more time to prepare for second year exams.

SUPPLEMENT 5: SMALL TELESCOPES FIGURE

Figure S5. Small telescopes results. The figure includes each original effect size, the corresponding aggregated replication effect size, and the d33% line indicating the smallest effect size that would be reasonably detectable with the original study design. Note that the original "Higher Standard" study reported one significant effect and one nonsignificant one, and that the "Presumption of Guilt" effect was originally a null finding.

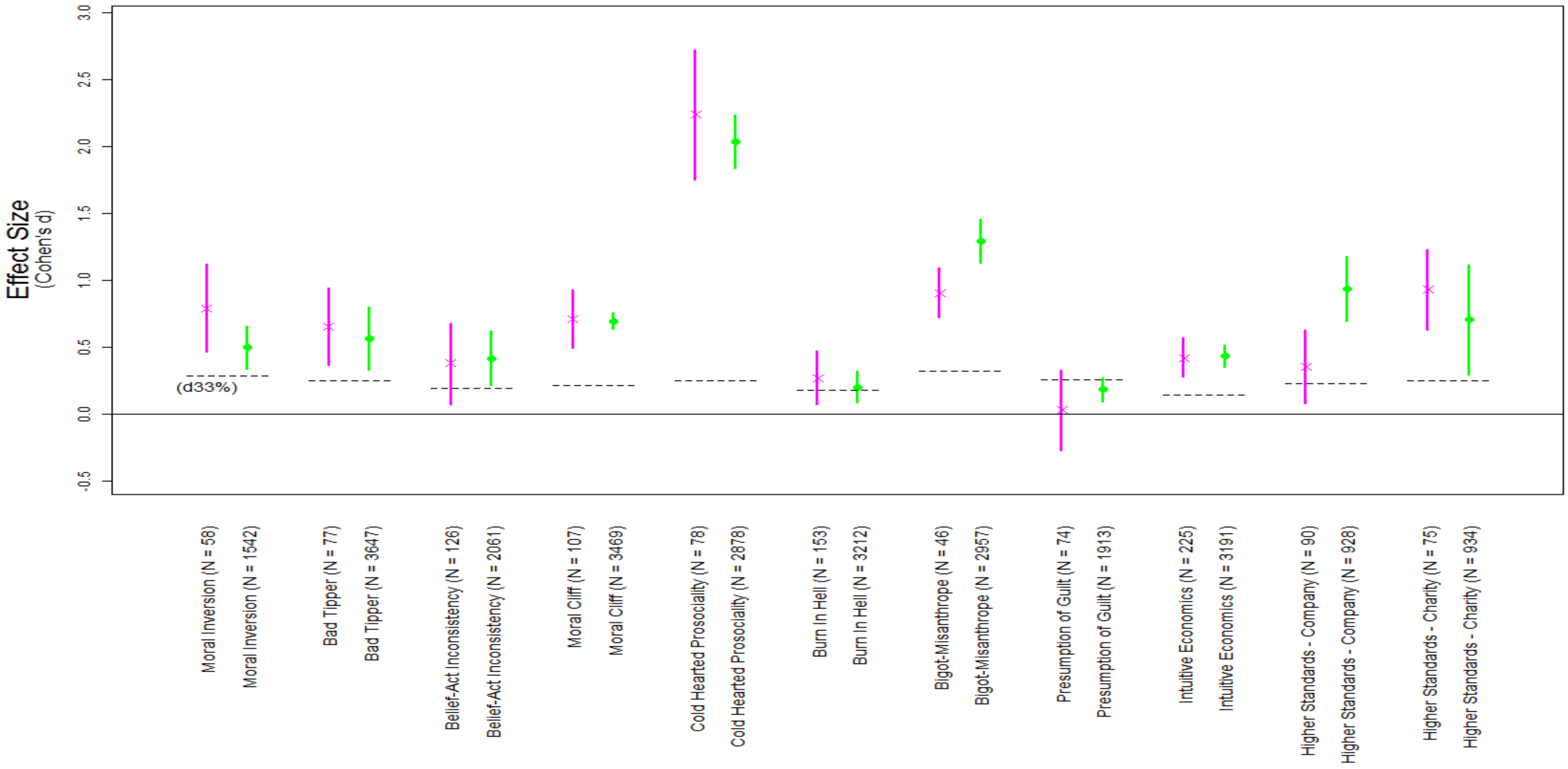

SUPPLEMENT 6: MODERATOR ANALYSES

Moral Inversion Effect

IV: *mi_condition*
DV: *MI_moralgood*

Original analysis: ANOVA
Moderator analyses: Ran ANOVAs/regression analyses to examine how the various moderators might interact with the main effect.

Moderator 1: USA vs. non-USA replication location

	USA (1)	Non-USA (0)
No Contribution (1)	5.18_a (1.07)	5.24_a (1.41)
Charity (3)	4.29_b (1.92)	4.59_c (1.90)

Condition: $F(1,1538) = 51.28, p < .001, \eta_p^2 = .03$
USA: $F(1,1538) = 1.23, p = .27, \eta_p^2 = .001$
Cond*USA: $F(1,1538) = 2.86, p = .09, \eta_p^2 = .002$

There is a main effect of condition, no main effect of USA, and a marginally-significant interaction. There is a difference between the *no contribution* and *charity condition* for both the USA, $t(1538) = -10.08, p < .001$, and the non-USA samples, $t(1538) = -3.04, p = .002$.

Moderator 2: Student sample vs. general population

	Student (1)	General (0)
No Contribution (1)	5.28 (1.33)	5.19 (1.36)
Charity (3)	4.46 (1.88)	4.25 (1.95)

Condition: $F(1,1538) = 106.78, p < .001, \eta_p^2 = .07$
Student: $F(1,1538) = 3.17, p = .08, \eta_p^2 = .002$
Cond*Student: $F(1,1538) = 0.41, p = .52, \eta_p^2 < .001$

There is a main effect of condition, a main effect of student versus general population sample, and no interaction.

Moderator 3: Same vs. different location

	Same (1)	Different (0)
No Contribution (1)	5.27_a (1.36)	5.21_a (1.35)
Charity (3)	4.13_b (2.03)	4.46_c (1.85)

Condition: $F(1,1538) = 111.11$, $p < .001$, $\eta_p^2 = .07$
Same: $F(1,1538) = 2.26$, $p = .13$, $\eta_p^2 = .001$
Cond*Same: $F(1,1538) = 4.86$, $p = .03$, $\eta_p^2 = .003$

There is a main effect of condition, no main effect of same versus different study location, and a significant interaction. There is a difference between the *charity* vs. *no contribution conditions* when done in the same location, $t(1538) = -7.78$, $p < .001$, and when done in a different location, $t(1538) = -7.28$, $p < .001$.

Moderator 4: Study order

	1st study in packet	2nd study in packet	3rd study in packet
No Contribution (1)	5.34 (1.33)	5.28 (1.36)	5.11 (1.36)
Charity (3)	4.49 (1.91)	4.18 (1.86)	4.38 (1.96)

Condition: $F(1,1535) = 109.62$, $p < .001$, $\eta_p^2 = .07$
Order: $F(2,1535) = 1.93$, $p = .15$, $\eta_p^2 = .003$
Cond*Order: $F(2,1535) = 1.60$, $p = .20$, $\eta_p^2 = .002$

There is a only a main effect of condition.

Intuitive Economics

Variables: *ie12com_htxfair* and *ie12comb_htxgood*

Original Analysis: a correlation between *ie12com_htxfair* and *ie12comb_htxgood*
Moderator analyses: Selected cases by moderator variable, recorded the *r*, and performed t-tests on the *r*s.

To test the differences between these correlations, we used the Hausman Test to test the z-score:

z-value = $(r_1 - r_2)$/[sqrt((SE_r_1)^2 - (SE_r_2)^2)]

where
z-value = critical value (1.96 means p < .05; 1.28 means p < .10).
r_1 = correlation 1
r_2 = correlation 2
sqrt = square root
SE = standard error
^2 = quantity squared

And SE_r is calculated via:

sqrt((1-r^2)/n-2)

Moderator 1: USA vs. non-USA sample

USA: $r = .52$, $p < .001$, n = 2615
Non-USA: $r = .25$, $p < .001$, n = 574

Same directionality, such that economic variables perceived as unfair are seen as especially bad for the economy. But the correlation is double in magnitude for the USA sample. With a Hausman *z* of 7.32, this difference is highly significant.

Moderator 2: Student sample vs. general population

Students: $r = .39$, $p < .001$, n = 1541
General: $r = .54$, $p < .001$, n = 1648

Same directionality, but with a higher correlation in the general population than in student samples. With a Hausman *z* of -13.66, this difference is highly significant.

Moderator 3: Same vs. different location

Same: $r = .51$, $p < .001$, n = 93
Different: $r = .48$, $p < .001$, n = 3096

Almost identical correlations. With a Hausman z of .34, the difference between these correlations is not significant.

Moderator 4: Study order

1st position in packet: $r = .48$, $p < .001$, n = 885
2nd position in packet: $r = .48$, $p < .001$, n = 1317
3rd position in packet: $r = .49$, $p < .001$, n = 894

Almost identical correlations. With a Hausman z of -.28, the difference between these correlations is not significant.

Burn in Hell

Variables: *BIH_executives* and *BIH_vandals*

Original Analysis: t-test comparing ratings of *BIH_executives* with ratings of *BIH_vandals*
Moderator analyses: As it was a paired, within subjects t-test, we ran a repeated measures ANOVA with the various moderator variables.

Moderator 1: USA vs. Non-USA sample

USA (n = 2522)
Executives - M: 37.91, SD: 32.30
Vandals - M: 28.42, SD: 29.01

Non-USA (n = 690)
Executives - M: 34.71, SD: 27.37
Vandals - M: 29.87, SD: 28.97

Exec_Vandal: $F(1, 3210) = 89.95$, $p < .001$, $\eta_p^2 = 0.03$
Exec_Vandal * USA: $F(1, 3210) = 9.44$, $p = .002$, $\eta_p^2 = 0.002$

The main effect of Exec_Vandal Remains. There is also an interaction such that the difference in the USA sample is larger than the difference in the Non-USA sample.

Moderator 2: Student sample vs. general population

Students (n = 1724)
Executives - M: 33.32, SD: 28.14
Vandals - M: 29.43, SD: 29.17

General (n = 1488)
Executives - M: 41.74, SD: 34.12
Vandals - M: 27.92, SD: 28.79

Exec_Vandal: $F(1, 3210) = 205.94$, $p < .001$, $\eta_p^2 = 0.06$
Exec_Vandal * Student: $F(1, 3210) = 64.80$, $p < .001$, $\eta_p^2 = 0.02$

Main effect of Exec_Vandal remains. There is also an interaction such that the difference in the general population sample is larger than the difference in the student sample.

Moderator 3: Same vs. different location

Same (n = 180)
Executives - M: 31.06, SD: 26.99
Vandals - M: 24.69, SD: 23.86

Different (n = 3032)
Executives - M: 37.59, SD: 31.54
Vandals - M: 28.97, SD: 29.26

Exec_Vandal: $F(1, 3210) = 30.76$, $p < .001$, $\eta_p^2 = 0.01$
Exec_Vandal * Location: $F(1, 3210) = 0.69$, $p = .41$, $\eta_p^2 < 0.001$

Main effect of Exec_Vandal Remains. There is also an interaction such that the size of the effect is greater in the Different locations than in the Same location.

Moderator 4: Study order

	1st study in packet	2nd study in packet	3rd study in packet
Executives	39.02 (30.06)	36.96 (30.87)	36.21 (34.95)
Vandals	29.33 (29.10)	27.70 (28.79)	28.82 (29.97)

Condition: $F(1,2926) = 169.37$, $p < .001$, $\eta_p^2 = .06$
Order: $F(2,2926) = 1.82$, $p = .16$, $\eta_p^2 = .001$
Cond*Order: $F(2,2926) = 1.08$, $p = .34$, $\eta_p^2 = .001$

There is a only a main effect of condition.

Presumption of Guilt

IV: *presumption_condition* (only Conditions 1 (*no investigation*) and 4 (*guilty)*)
DV: *PG_companyevaluation*

Original Analysis: T-test between Conditions 1 and 4
Moderator analyses: Rn ANOVAs/regressions to see if the main effect is moderated by the moderator variables.

Moderator 1: USA vs. non-USA sample

	Non-USA (0)	USA (1)
Do nothing (1)	3.41 (1.57)	3.42 (1.53)
Guilty (4)	3.61 (1.65)	3.75 (1.87)

Condition: $F(1,1909) = 10.34, p = .001, \eta_p^2 = .01$
USA: $F(1,1909) = 0.82, p = .37, \eta_p^2 < .001$
Cond*USA: $F(1,1909) = 0.66, p = .42, \eta_p^2 < .001$

Contrary to the original study, there is a significant main effect of condition, such that doing nothing actually leads to significantly *worse* reputation ratings than being found guilty (the original study found no difference between the two conditions). No interaction with USA vs. non-USA sample.

Moderator 2: Student sample vs. general population

	General (0)	Student (1)
Do Nothing (1)	3.43 (1.56)	3.41 (1.53)
Guilty (4)	3.68 (1.78)	3.72 (1.81)

Condition: $F(1,1909) = 12.20, p = .001, \eta_p^2 = .01$
Student: $F(1,1909) = 0.27, p = .87, \eta_p^2 < .001$
Cond*Student: $F(1,1909) = 0.14, p = .71, \eta_p^2 < .001$

Contrary to the original study, there is a significant main effect of condition, such that doing nothing actually leads to significantly *worse* reputation ratings than being found guilty. This does not vary by student samples vs. the general population.

Moderator 3: Same vs. different location

	Same (1)	Different (0)
Do Nothing (1)	3.99 (1.29)	3.39 (1.55)
Guilty (4)	4.35 (1.83)	3.67 (1.79)

Condition: $F(1,1909) = 3.029$, $p = .082$, $\eta_p^2 = .02$
Location: $F(1,1909) = 12.346$, $p < .001$, $\eta_p^2 = .006$
Cond*Location: $F(1,1909) = .046$, $p = .83$, $\eta_p^2 < .001$

Contrary to the original study, there is a significant main effect of condition, such that doing nothing actually leads to significantly *worse* reputation ratings than being found guilty. This does not vary systematically by study location (same vs. different).

Moderator 4: Study order

	1st study in packet	2nd study in packet	3rd study in packet
Do Nothing (1)	3.59 (1.61)	3.23 (1.44)	3.28 (1.56)
Guilty (4)	3.73 (1.83)	3.55 (1.90)	3.67 (1.63)

Condition: $F(1, 1766) = 12.71$, $p < .001$, $\eta_p^2 = .07$
Order: $F(2, 1766) = 3.98$, $p = .02$, $\eta_p^2 = .004$
Cond*Order: $F(2, 1766) = .80$, $p = .45$, $\eta_p^2 = .001$

There is a main effect for condition and a main effect of order such that ratings for both dependent measures are higher when the study appears earlier in the study packet.

Moral Cliff

Variables: *mc_ps_dishonesty* and *mc_dishonesty*

Original Analysis: t-test to see if ratings of *mc_ps_dishonesty* were higher than ratings of *mc_dishonesty.*
Moderator analyses: As the original analysis was a paired, within subjects t-test, ran a repeated measures ANOVA with moderator variables.

Moderator 1: USA vs. non-USA sample

USA (n = 2326)
Photoshop - M: 5.37, SD: 1.23
Control - M: 4.40, SD: 1.33

Non-USA (n = 1143)
Photoshop - M: 5.30, SD: 1.22
Control - M: 4.53, SD: 1.29

Photo_Ctrl: $F(1, 3467) = 1218.17, p < .001, \eta_p^2 = 0.26$
Photo_Ctrl * USA: $F(1, 3467) = 14.26, p < .001, \eta_p^2 = 0.004$

The original difference between *Photoshop* and *Control* replicates. But there is also significant moderation effect, such that this “Moral Cliff” effect is smaller in the non-USA samples than in the USA samples.

Moderator 2: Student sample vs. general population

General population (n = 1398)
Photoshop - M: 5.46, SD: 1.21
Control - M: 4.51, SD: 1.36

Student sample (n = 2071)
Photoshop - M: 5.27, SD: 1.22
Control - M: 4.40, SD: 1.29

Photo_Ctrl: $F(1, 3467) = 1445.99, p < .001, \eta_p^2 = 0.29$
Photo_Ctrl * Student: $F(1, 3467) = 2.75, p = .01, \eta_p^2 = 0.001$

The original difference between Photoshop and Control replicates. But there is also a moderation effect, such that this “Moral Cliff” effect is larger in the general population than it is for the student samples.

Moderator 3: Same vs. different location

Different location (n = 2485)
Photoshop - M: 5.31, SD: 1.22
Control - M: 4.46, SD: 1.31

Same location (n = 984)
Photoshop - M: 5.42, SD: 1.22
Control - M: 4.40, SD: 1.33

Photo_Ctrl: $F(1, 3467) = 1299.41$, $p < .001$, $\eta_p^2 = 0.27$
Photo_Ctrl * Location: $F(1, 3467) = 9.90$, $p = .002$, $\eta_p^2 = 0.003$

The original difference between *Photoshop* and *Control* replicates. But there is also a moderation effect, such that the difference between the two conditions is smaller when the study was done in a different location than when it was done in the same location as the original study.

Moderator 4: Study order

	1st study in packet	2nd study in packet	3rd study in packet
Photoshop	5.26 (1.19)	5.40 (1.23)	5.38 (1.24)
Control	4.40 (1.28)	4.46 (1.34)	4.48 (1.34)

Condition: $F(1, 3463) = 1473.13$, $p < .001$, $\eta_p^2 = .30$
Order: $F(2, 3463) = 3.26$, $p = .04$, $\eta_p^2 = .002$
Cond*Order: $F(2, 3463) = .95$, $p = .39$, $\eta_p^2 = .001$

There was a main effect for condition and a main effect of order such that ratings for both dependent measures are higher when the study appears later in the study packet.

Bad Tipper

IV: *tipper_condition* (1 (*penny*) vs. 2 (*less tip*))
DV: *tipper_personjudge*

Original Analysis: T-test between Conditions 1 and 2
Moderator analyses: Ran ANOVAs/regressions to see if the main effect is moderated by the moderator variables.

Moderator 1: USA vs. non-USA sample

	Non-USA (0)	USA (1)
Pennies (1)	3.87_a (1.18)	4.27_b (1.28)
Less Tip (2)	3.51_c (1.34)	3.23_d (1.25)

Condition: $F(1,3643) = 252.04, p < .001, \eta_p^2 = .07$
US: $F(1,3643) = 1.92, p = .17, \eta_p^2 = .001$
Cond*US: $F(1,3643) = 59.87, p = .01, \eta_p^2 = .02$

The original main effect of pennies vs. less tip replicates. But there is also an interaction with USA versus non-USA sample. The difference between the Pennies and Less Tip condition is significant for both the non-USA samples, $t(3643) = -5.04, p < .001$, and USA samples, $t(3643) = -19.99, p < .001$, but the difference is larger for the USA samples.

Moderator 2: General vs. Student

	General (0)	Student (1)
Pennies (1)	4.27_a (1.29)	4.04_b (1.24)
Less Tip (2)	3.07_c (1.19)	3.50_d (1.32)

Condition: $F(1,3643) = 412.55, p < .001, \eta_p^2 = .10$
Student: $F(1,3643) = 5.08, p = .02, \eta_p^2 = .001$
Cond*Student: $F(1,3643) = 57.60, p < .001, \eta_p^2 = .02$

The original main effect of pennies versus less tip replicates. There is also an interaction with student sample vs. general population. The difference between the *Pennies* and *Less Tip* condition is significant for both the general population samples, $t(3643) = -17.86, p < .001$, and student samples, $t(3643) = -10.19, p < .001$, but the difference is larger in the general population.

Moderator 3: Same vs. different location

	Different (0)	Same (1)
Pennies (1)	4.03_a (1.22)	4.41_c (1.32)
Less Tip (2)	3.42_b (1.28)	3.09_d (1.27)

Condition: $F(1,3643) = 417.86, p < .001, \eta_p^2 = .10$
Student: $F(1,3643) = 0.32, p = .57, \eta_p^2 < .001$
Cond*Student: $F(1,3643) = 56.75, p < .001, \eta_p^2 = .02$

The original main effect of pennies versus less tip holds. But there is also an interaction with different population vs. same population. The difference between the *Pennies* and *Less Tip* conditions is significant for both the different locations, $t(3643) = -12.34, p < .01$, and same location, $t(3643) = -16.41, p < .001$, samples. However, the magnitude of difference is larger in the same subject population than in the other populations.

Moderator 4: Study order

	1st study in packet	2nd study in packet	3rd study in packet
Pennies (1)	4.18 (1.19)	4.20 (1.32)	4.02 (1.30)
Less Tip (2)	3.27 (1.23)	3.33 (1.29)	3.33 (1.34)

Condition: $F(1, 3538) = 366.50, p < .001, \eta_p^2 = .09$
Order: $F(2, 3538) = 1.35, p = .26, \eta_p^2 = .001$
Cond*Order: $F(2, 3538) = 2.34, p = .10, \eta_p^2 = .001$

There is only a main effect of condition.

Higher Standards: Company Conditions

IV: standard_condition
DV: standard_eval_7items

Original Analysis: T-test between Conditions 3 (*small perk*) and 1 (*monetary-salary only*)
Moderator analyses: Ran ANOVAs/regressions to see if the main effect was moderated by the various moderator variables.

Moderator 1: USA vs. non-USA sample

	Non-USA (0)	USA (1)
No Perk (1)	3.97 (0.87)	4.05 (0.93)
Small Perk (3)	3.32 (1.04)	2.97 (1.08)

Condition: $F(1,910) = 88.29, p < .001, \eta_p^2 = .09$
USA: $F(1,910) = 2.09, p = .15, \eta_p^2 = .002$
Cond*USA: $F(1,918) = 5.43, p = .02, \eta_p^2 = .006$

Contrary to the findings of the original study, there is a significant main effect of no perk versus small perk for a company. There is also an interaction between USA vs. non-USA samples. The difference between the *No Perk* and *Small Perk* conditions holds for both the non-USA sample, $t(910) = -3.84, p < .001$, and USA sample, $t(910) = -14.94, p < .001$. However, the magnitude of the difference is larger in the USA sample.

Moderator 2: Student sample vs. general population

	General (0)	Student (1)
No Perk (1)	4.04 (0.95)	4.03 (0.88)
Small Perk (3)	3.01 (1.11)	3.06 (1.04)

Condition: $F(1,910) = 219.20, p < .001, \eta_p^2 = .19$
Student: $F(1,910) = 0.13, p = .72, \eta_p^2 < .001$
Cond*Student: $F(1,910) =.17, p = .68, \eta_p^2 < .001$

Contrary to the original findings, there is a significant main effect of no perk versus small perk for a company. There is no interaction with type of sample (student vs. general population).

Moderator 3: Same vs. different location

	Different (0)	Same (1)
No Perk (1)	3.96_a (0.85)	4.16_b (1.02)
Small Perk (3)	3.20_c (1.02)	2.72_d (1.13)

Condition: $F(1{,}910) = 261.21$, $p < .001$, $\eta_p^2 = .22$
Location: $F(1{,}910) = 4.04$, $p = .05$, $\eta_p^2 = .004$
Cond*Location: $F(1{,}910) = 24.37$, $p < .001$, $\eta_p^2 = .03$

Contrary to the findings of the original study, there is a significant main effect of no versus small perk for a company. There is also an interaction between same versus different location. The difference between the *No Perk* and *Small Perk* conditions holds for both the different location, $t(910) = -9.38$, $p < .001$, and same location, $t(910) = -13.16$, $p < .001$, samples. However, the magnitude of the difference is larger in the same location sample.

Moderator 4: Study order

	1st study in packet	2nd study in packet	3rd study in packet	4th study in packet
No Perk (1)	3.92 (.93)	4.06 (.78)	4.07 (.98)	4.10 (.98)
Small Perk (3)	2.92 (1.10)	3.05 (1.11)	3.17 (1.08)	2.97 (1.02)

Condition: $F(1, 906) = 231.50$, $p < .001$, $\eta_p^2 = .20$
Order: $F(3, 906) = 1.58$, $p = .19$, $\eta_p^2 = .005$
Cond*Order: $F(3, 906) = .53$, $p = .66$, $\eta_p^2 = .002$

There is only a main effect of condition.

Higher Standard: Charity Conditions

Original Analysis: T-test between Conditions 4 (*monetary-salary only*) and 6 (*small perk*)
Moderator analyses: Ran ANOVAs/regressions to see if the main effect was moderated by the various moderator variables.

Moderator 1: USA vs. non-USA sample

	Non-USA (0)	USA (1)
No Perk (4)	4.03 (0.76)	3.98 (0.93)
Small Perk (6)	3.04 (1.32)	3.03 (1.25)

Condition: $F(1,921) = 98.72$, $p < .001$, $\eta_p^2 = .10$
USA: $F(1,921) =.07$, $p = .79$, $\eta_p^2 < .001$
Cond*USA: $F(1,921) = 0.04$, $p = .85$, $\eta_p^2 < .001$

Only the original main effect of no perk versus small perk holds.

Moderator 2: Student sample vs. general population

	General (0)	Student (1)
No Perk (4)	3.96 (0.94)	4.03 (0.84)
Small Perk (6)	2.98 (1.30)	3.10 (1.21)

Condition: $F(1,921) = 168.01$, $p < .001$, $\eta_p^2 = .15$
Student: $F(1,921) = 1.52$, $p = .22$, $\eta_p^2 = .002$
Cond*Student: $F(1,921) = 0.13$, $p = .72$, $\eta_p^2 < .001$

Only the original main effect of no versus small perk holds.

Moderator 3: Same vs. different location

	Different (0)	Same (1)
No Perk (4)	4.03 (0.85)	3.91 (0.98)
Small Perk (6)	3.03 (1.22)	3.04 (1.33)

Condition: $F(1,921) = 156.77$, $p < .001$, $\eta_p^2 = .15$
Location: $F(1,921) = 0.49$, $p = .48$, $\eta_p^2 = .001$
Cond*Location: $F(1,921) = 0.75$, $p = .39$, $\eta_p^2 < .001$

Only the original main effect of no versus small perk holds.

Moderator 4: Study order

	1st study in packet	2nd study in packet	3rd study in packet	4th study in packet
No Perk (4)	4.00 (.82)	4.00 (.84)	4.12 (.99)	3.83 (.94)
Small Perk (6)	2.91 (1.42)	3.12 (1.31)	3.15 (1.22)	2.93 (1.08)

Condition: $F(1, 917) = 177.12$, $p < .001$, $\eta_p^2 = .16$
Order: $F(3, 917) = 2.48$, $p = .06$, $\eta_p^2 = .008$
Cond*Order: $F(3, 917) = .42$, $p = .74$, $\eta_p^2 = .001$

There is only a main effect of condition.

Cold-Hearted Prosociality

Variables: *cold_moral* & *cold_traits*

Original Analysis: t-test comparing ratings of *cold_moral* with ratings of *cold_traits*
Moderator analyses: As the original study used a paired, within subjects t-test, to test moderators we used a repeated measures ANOVA with various moderator variables.

Moderator 1: USA vs. non-USA samples

Non-USA (n = 539)
Moral - M: 2.31, SD: 1.22
Traits - M: 4.38, SD: 0.85

USA (n = 2371)
Moral - M: 2.19, SD: 1.26
Traits - M: 4.47, SD: 1.01

Moral_Traits: $F(1, 2908) = 4171.76$, $p < .001$, $\eta_p^2 = 0.58$
USA: $F(1, 2908) = .091$, $p = .76$, $\eta_p^2 < .001$
Moral_Traits * USA: $F(1, 2908) = 9.06$, $p < .003$, $\eta_p^2 = 0.03$

The original difference between Moral Acts and Traits replicates. But there is also a moderation effect, such that the effect is smaller in the non-USA samples than in the USA samples.

Moderator 2: General vs. Students

General (n = 1657)
Moral - M: 2.22, SD: 1.29
Traits - M: 4.52, SD: 1.05

Students (n = 1253)
Moral - M: 2.21, SD: 1.20
Traits - M: 4.36, SD: 0.90

Moral_Traits: $F(1, 2908) = 7113.12$, $p < .001$, $\eta_p^2 = 0.71$
Student: $F(1, 2908) = 6.21$, $p < .001$, $\eta_p^2 = 0.002$
Moral_Traits * Student: $F(1, 2908) = 7.37$, $p = .007$, $\eta_p^2 = 0.003$

The original difference between Moral Acts and Traits replicates. But there is also a moderation effect, such that the difference between the two conditions is larger in the general population than in the student samples.

Moderator 3: Same vs. different location

Different (n = 1917)
Moral - M: 2.16, SD: 1.17
Traits - M: 4.37, SD: 0.88

Same (n = 993)
Moral - M: 2.31, SD: 1.39
Traits - M: 4.61, SD: 1.14

Moral_Traits: $F(1, 2908) = 6660.85$, $p < .001$, $\eta_p^2 = 0.70$
Location: $F(1, 2908) = 33.62$, $p < .001$, $\eta_p^2 = 0.001$
Moral_Traits * Location: $F(1, 2908) = 3.07$, $p = .08$, $\eta_p^2 = 0.001$

The original difference between Moral Acts and Traits replicates.

Moderator 4: Study order

	1st study in packet	2nd study in packet	3rd study in packet	4th study in packet
Moral	2.17 (1.23)	2.16 (1.23)	2.28 (1.28)	2.21 (1.27)
Traits	4.48 (.99)	4.47 (.94)	4.46 (1.00)	4.42 (.99)

Condition: $F(1, 2809) = 7243.05$, $p < .001$, $\eta_p^2 = .72$
Order: $F(3, 2809) = .61$, $p = .61$, $\eta_p^2 = .001$
Cond*Order: $F(3, 2809) = 1.66$, $p = .18$, $\eta_p^2 = .002$

There is only a main effect of condition.

Bigot Misanthrope

Variables: *bigot_personjudge*

Original Analysis: t-test comparing ratings of *bigot_personjudge* with the scale midpoint of 4. Moderator analyses: One-sample t-tests against the midpoint of the scale for each level of the moderators to examine whether effect holds at each level of the moderator. Between subjects t-test with moderator as the independent variable to examine whether the effect is moderated.

Moderator 1: USA vs. non-USA samples

Non-USA (n = 579)
PersonJudge - M: 2.05, SD: 1.16
One-sample t-test against the midpoint of the scale: $t(578) = -40.247$, $p < .001$; 95% Confidence interval of the difference: [-2.05, -1.86]

USA (n = 2378)
PersonJudge - M: 2.47, SD: 1.39
One-sample t-test against the midpoint of the scale: $t(2377) = -53.74$, $p < .001$; 95% Confidence interval of the difference: [-1.59, -1.48]

The effect replicates in both samples, but the non-overlapping 95% confidence intervals also suggest a moderation effect, such that the bigot-misanthrope effect is weaker in the USA sample than in the non-USA sample.

Moderator 2: Student samples vs. general population

General (n = 1682)
PersonJudge - M: 2.51, SD: 1.39
One-sample t-test against the midpoint of the scale: $t(1682) = -43.93$, $p < .001$; 95% Confidence interval of the difference: [-1.56, -1.43]

Students (n = 1275)
PersonJudge - M: 2.22, SD: 1.30
One-sample t-test against the midpoint of the scale: $t(1274) = -48.88$, $p < .001$; 95% Confidence interval of the difference: [-1.85, -1.71]

Between-subjects t-test with student samples vs. general samples as independent variable: $t(2834.08) = 5.70$, $p < .001$.

The effect replicates in both samples, but the non-overlapping 95% confidence intervals also suggest a moderation effect, such that the bigot-misanthrope effect is weaker in the general population than the student sample.

Moderator 3: Same vs. different location

Different (n = 1957)
PersonJudge - M: 2.29, SD: 1.30
One-sample t-test against the midpoint of the scale: $t(1956) = -58.32$, $p < .001$; 95% Confidence interval of the difference: [-1.77, -1.65]

Same (n = 1000)
PersonJudge - M: 2.57, SD: 1.46
One-sample t-test against the midpoint of the scale: $t(999) = -30.98$, $p < .001$; 95% Confidence interval of the difference: [-1.52, -1.34]

Between-subjects t-test with same vs. different location as independent variable: $t(1821.32) = -5.21$, $p < .001$.

The effect replicates in both samples, but the non-overlapping 95% confidence intervals also suggest a moderation effect such that the bigot-misanthrope effect is weaker in the same location than in a different location.

Moderator 4: Study order

1st study in packet (n = 682)
PersonJudge - M: 2.49, SD: 1.36
One-sample t-test against the midpoint of the scale: $t(681) = -29.04$, $p < .001$; 95% Confidence interval of the difference: [-1.61, -1.41]

2nd study in packet (n =645)
PersonJudge - M: 2.43, SD: 1.36
One-sample t-test against the midpoint of the scale: $t(644) = -29.50$, $p < .001$; 95% Confidence interval of the difference: [-1.68, -1.47]

3rd study in packet (n = 638)
PersonJudge - M: 2.35, SD: 1.39
One-sample t-test against the midpoint of the scale: $t(637) = -30.00$, $p < .001$; 95% Confidence interval of the difference: [-1.76, -1.54]

4th study in packet (n = 641)
PersonJudge - M: 2.50, SD: 1.39
One-sample t-test against the midpoint of the scale: $t(640) = -27.26$, $p < .001$; 95% Confidence interval of the difference: [-1.61, -1.39]

Oneway ANOVA with study order as independent variable: $F(3, 2602) = 1.68$, $p < .17$. There is no moderating effect of study order.

Belief-Act Inconsistency

IV: *belief-_condition* (3 (big game hunting) vs. 1 (animal rights))
DV: *beliefact_mrlblmw_rec*

Original Analysis: T-test between conditions 3 and 1.
Moderator analyses: Run ANOVAs/regressions to see if the main effect is moderated by our various moderator variables.

Moderator 1: USA vs. non-USA

	Non-US (0)	US (1)
Animal Rights (1)	-3.21 (2.19)	-2.43 (2.49)
Big Game Hunting (3)	-2.76 (2.21)	-1.64 (2.38)

Condition: $F(1,1978) = 19.94$, $p < .001$, $\eta_p^2 = .01$
US: $F(1,1978) = 46.42$, $p < .001$, $\eta_p^2 = .02$
Cond*US: $F(1,1978) = 1.46$, $p = .22$, $\eta_p^2 = .001$

Main effect of condition still stands. Also a main effect of location such that USA samples provide lower ratings than non-USA samples. No interaction effect.

Moderator 2: Student samples vs. general population

	General (0)	Students (1)
Animal Rights (1)	-2.54 (2.45)	-2.63 (2.46)
Big Game Hunting (3)	-1.81 (2.40)	-1.88 (2.38)

Condition: $F(1,1978) = 44.55$, $p < .001$, $\eta_p^2 = .02$
Population: $F(1,1978) = 0.57$, $p = .45$, $\eta_p^2 < .001$
Cond*Population: $F(1,1978) = 0.01$, $p = .91$, $\eta_p^2 < .001$

Original main effect still holds. No main effect of population. No interaction.

Moderator 3: Same vs. different location

	Different (0)	Same (1)
Animal Rights (1)	-2.57 (2.46)	-2.48 (2.14)
Big Game Hunting (3)	-1.79 (2.40)	-1.46 (1.89)

Condition: $F(1,2063) = 16.73$, $p < .001$, $\eta_p^2 < .008$
Location: $F(1,2063) = .88$, $p = .35$, $\eta_p^2 < .001$
Cond*Location: $F(1,2063) = .28$, $p = .596$, $\eta_p^2 < .001$

Original main effect still holds. No main effect of location. No interaction.

Moderator 4: Study order

	1st study in packet	2nd study in packet	3rd study in packet	4th study in packet
Animal Rights (1)	-2.51 (2.51)	-2.34 (2.64)	-2.85 (2.26)	-2.55 (2.41)
Big Game Hunting (3)	-1.76 (2.52)	-2.21 (2.19)	-1.41 (2.28)	-1.69 (2.55)

Condition: $F(1, 1866) = 50.44$, $p < .001$, $\eta_p^2 = .03$
Order: $F(3, 1866) = .43$, $p = .74$, $\eta_p^2 = .001$
Cond*Order: $F(3, 1866) = 5.68$, $p = .001$, $\eta_p^2 = .009$

There is a main effect of condition and an interaction effect such that the hypothesized effect is stronger when the study appears later in the packet rather than earlier.
